# Two New Species and First Stage Associations for Two Other Species of the *Cincticostella nigra* (Uéno, 1928) Complex (Ephemeroptera, Ephemerellidae) from Yunnan, China, with Discussion About *Cincticostella* Allen, 1971 Species Complexes Based on Winged Stages †

**DOI:** 10.3390/insects16121221

**Published:** 2025-11-29

**Authors:** Yi-Fei Feng, Yan-Chang Zi, Cheng-Fa Zhao, Yuan Mu, Xian-Fu Li, Luke M. Jacobus

**Affiliations:** 1 Institute of Eastern-Himalaya Biodiversity Research, Dali University, Dali 671003, China; fyf525458@163.com (Y.-F.F.); yanchang.zi@outlook.com (Y.-C.Z.); zhaocf@eastern-himalaya.cn (C.-F.Z.); muy@eastern-himalaya.cn (Y.M.); 2 Center for Interdisciplinary Sciences, Dali University, Dali 671003, China; 3 Collaborative Innovation Center for Biodiversity and Conservation in the Three Parallel Rivers Region of China, Dali University, Dali 671003, China; 4 Research Center of Ecology and Governance for Er’hai Lake Streams, Dali 671003, China; 5 Division of Science, Indiana University Columbus, Columbus, IN 47203, USA; 6 Department of Entomology, Purdue University, West Lafayette, IN 47907, USA

**Keywords:** spiny crawler mayflies, key, phylogeny, imago, Hengduan Mountains, Himalaya

## Abstract

Four species of the *Cincticostella nigra* (Uéno, 1928) complex (Ephemeroptera, Ephemerellidae) from Yunnan, China are described based on egg chorionic structure and nymph and winged stages, including *C. wangi* Selvakumar Martynov & Subramanian, 2021, *C. funki* Martynov, Selvakumar, Palatov & Vasanth, 2021, and two new species, viz., *Cincticostella xiazhi* Zi, Li & Jacobus, **sp. nov.** and *Cincticostella yushui* Zi, Li & Jacobus, **sp. nov.** The egg and winged stages of *C. wangi* and *C. funki* are described for the first time. Eggs of all four species have macroreliefs generally similar to one another and to other species in the genus. Male imagoes have preliminary species-level differences in the median convex lobes of the styliger plate and the shapes of the penis lobes themselves. The current delimitation of four complexes of *Cincticostella* is briefly discussed based on the nymph and winged stages. A partial phylogenetic reconstruction of *Cincticostella* based on the COI gene illustrates relations of representatives of the four complexes on the one hand and distinct delimitation of *C. wangi*, *C. funki*, *C. xiazhi*, **sp. nov.**, and *C. yushui*, **sp. nov.** on the other. An updated list of species of *Cincticostella* is provided, along with a new key for the identification of nymphs of the *C. nigra* complex, which are distinguished primarily based on features of the abdominal terga, mesothoracic projections, tarsal claws, labrum emargination, setation of femora, and body color.

## Simple Summary

The winged stages are very important for the classification of the genus *Cincticostella* Allen, 1971. Unfortunately, winged stages of most *Cincticostella* species are poorly known or unknown. Moreover, at least in China, there are a significant number of undescribed new species. The present study focuses on the winged stages of *Cincticostella* species from Yunnan, China. Two new species of *Cincticostella xiazhi* Zi, Li & Jacobus, **sp. nov.** and *C. yushui* Zi, Li & Jacobus, **sp. nov.** are described based on egg chorionic structure and nymph and winged stages, and the eggs and winged stages of *C. wangi* Selvakumar Martynov & Subramanian, 2021 and *C. funki* Martynov, Selvakumar, Palatov & Vasanth, 2021 are described for the first time. The delimitation of four species complexes in *Cincticostella* is discussed based on the winged stages. Additionally, a partial phylogenetic reconstruction of *Cincticostella* based on the COI gene is proposed and discussed. Finally, a key to the nymphs of the *C. nigra* complex is included.

## 1. Introduction

Jacobus and McCafferty [1] were the last to revise the genera of the mayfly family Ephemerellidae (Ephemeroptera), providing our current working definition of the genus *Cincticostella* Allen, 1971 [2] (Ephemerellinae: Ephemerellini). According to the statistics of Martynov et al. [3], and the subsequent description of three species [4,5,6] (*C. ebura* Auychinda, Sartori & Boonsoong, 2022, *C. jianchuan* Sun, Tan, Li & Jacobus, 2024, and *C. parvula* Auychinda, Buchawongpiwat, Sartori & Boonsoong, 2025), the genus now includes twenty-five species (one of which is a *nomen dubium*) from throughout the eastern Palearctic and Indomalayan regions. The distinctive nymphs have the anterolateral angles of the prothorax projecting anteriorly and have a pair of large, wide, mesothoracic anterolateral processes, that differ from all other described Ephemerellidae species [1]. Allen effectively divided the genus into two species complexes consisting of the *insolta* and *nigra* complexes [7]; Martynov et al. [3] proposed the *gosei* complex; and Sun et al. [5] proposed the *jianchuan* complex. Therefore, four species complexes are currently considered at present: the *nigra*, *insolta*, *gosei*, and *jianchuan* complexes. Martynov et al. [3] presented distinguishing nymphal characters of *Cincticostella* species complexes. However, knowledge of the distinguishing characters of the winged stages of these complexes has been lacking.

The *C. insolta* complex includes eight species, with only one species reported from egg, nymphal, and imaginal stages [8]: *C. femorata* (Tshernova, 1972). The *C. nigra* complex includes thirteen species, with four species reported from egg, nymphal, and imaginal stages [9,10,11]: *C. elongatula* (McLachlan, 1875), *C. nigra* (Uéno, 1928), *C. levanidovae* (Tshernova, 1952), and *C. orientalis* (Tshernova, 1952); however, color images of those species are lacking. The *C. gosei* complex includes two species, with one of those reported from egg, nymphal, and imaginal stages [12]: *C. gosei* (Allen, 1975). The *C. jianchuan* complex includes two species, with both reported from egg, nymphal, and imaginal stages [5,13,14]: *C. fusca* (Kang & Yang, 1995) and *C. jianchuan* (Sun, Yang, Tan, Li & Jacobus, 2024).

The *C. nigra* complex has the highest number of species in the genus, and its systematics is also the most questionable. The winged stages are particularly poorly known in all geographic areas except for the far eastern Palearctic region. This complex has been relatively poorly investigated in the central and southern parts of China. Only two species of the *C. nigra* complex have been reported from this area [14,15]: *C. colossa* Kang & Yang, 1995 and *C. szechuanensis* Xie, Jia, Chen, Jacobus & Zhou, 2009. In the Palearctic region, four species of the *C. nigra* complex are reported from Japan [9], and some species are also distributed in northeast China [16,17], Korea [18], and Russia [19]. In the Indomalayan region, six species of the *C. nigra* complex have been reported from Thailand, India, Nepal, and the India–China border region [3,4]. The claws of species in the Palearctic region have more than five denticles, except *C. orientalis* (Tshernova, 1952) which has either one or two denticles [9]. The claws of species in the Indomalayan region only have one or two denticles, except *C. funki*, which rarely has three denticles [3,4]. The claws of *C. colossa* and *C. szechuanensis* also only have two denticles [14,15]. Species with intermediate numbers of denticles have not yet been reported.

In recent years, we collected specimens from the Hengduan Mountains, southwest China, where the species have characteristics that fall somewhere between the characterizations of the Indomalayan and Palearctic species [20]. For example, claws of one undescribed species have three denticles, and claws of others have four denticles.

Herein, we describe two new species and provide new geographic data for two species (*C. wangi* Selvakumar, Martynov & Subramanian, 2021 and *C. funki* Martynov, Selvakumar, Palatov & Vasanth, 2021) [3], all based on imago, subimago, nymph, and egg stages, and DNA sequence data (COI).

## 2. Materials and Methods

### 2.1. Specimen Sampling, Morphological Observation, and Storage

*Cincticostella* nymphs were collected with a D-frame net from riffle and run stream habitats. Following the guidelines of Li et al. [21] and Yang et al. [22], habitat photographs were taken using a mobile telephone equipped with a 40–75 mm macro lens (Kase, Jiangmen, China). Some specimens were dissected under a SZ680 stereo microscope (Cnoptec, Chongqing, China) and were mounted on slides with Hoyer’s solution (Kermel, Tianjing, China) for examination under higher light magnification. Slide-mounted specimens were examined, photographed, and measured under a VHX-S550E digital microscope (Keyence, Osaka, Japan). Eggs were dissected from female imagoes. Eggs were dried, coated with gold, observed, and photographed by a IB-3 scanning electron microscopy (JEOL Ltd., Akishima, Japan) at 1500× to 5000× magnification. The final plates were prepared with Adobe Photoshop CC 2018. All imagoes were collected by rearing final nymphal instars in the laboratory. All materials are stored in 95% ethanol (Gengma Hualin Alcohol Company Limited, Lincang, China) and are deposited in the Museum of Biology, Institute of Eastern-Himalaya Biodiversity Research, Dali University (MBDU).

Abbreviations used in this study are as follows: costa (C), subcosta (Sc), radial sector (Rs), radius (R1), medius anterior (MA), medius posterior (MP), cubitus posterior (CuP).

### 2.2. Molecular Study

Reared female imago specimens were fixed in 95% ethanol for description and DNA extraction. Reared female imagoes were chosen to avoid the influence of microbiota on the nymph body surface and in the digestive system. Collection details of the specimens of the four species used for the DNA experiment are shown in Table 1; one sequence was obtained for each species. Total genomic DNA was extracted using an Animal Genomic DNA Kit (Majorb bio, Shanghai, China), according to the manufacturer’s protocol. The cytochrome *c* oxidase subunit I (COI) gene was amplified by the universal primers LCO1490-JJ/HCO2198-JJ to obtain a 658 bp fragment corresponding to the DNA barcoding region [23]. Polymerase chain reaction (PCR) conditions follow Wesener [24]. The COI sequence was assembled by SeqMan using NOVOPlasty version 4.2 and Getorganelle version 1.7.0 (DNASTAR, Inc.). The preliminary phylogenetic tree was reconstructed using COI sequences of *C. wangi*, *C. funki*, *C. xiazhi* Zi, Li & Jacobus, **sp. nov.**, and *C. yushui* Zi, Li & Jacobus, **sp. nov.**, along with sequences of other *Cincticostella* species obtained from GenBank and the BOLD system (accessed on 3 September 2025). Using PhyloSuite for sequence alignment and editing [25], a preliminary phylogenetic tree was constructed, and the K2P genetic distance was estimated in MEGA12 [26]. The COI tree reconstruction method uses the maximum likelihood method to find the tree topology, branch length, and model parameters that maximize the probability of observed data appearing. The mean value was used to calculate pairwise distances for the species with more than one COI sequence.  *Torleya naga* Jacobus & McCafferty, 2004 [27] and *T. nepalica* (Allen & Edmunds, 1963) [28] sequences from GenBank were used as outgroups following the work of Auychinda et al. [6].

## 3. Results

### 3.1. Cincticostella wangi Selvakumar, Martynov & Subramanian, 2021

*Cincticostella wangi*: Martynov et al: figures 20–22 (nymph) [3]. Holotype and paratypes: nymphs, from India–China border region [3].

Figure 1, Figure 2, Figure 3, Figure 4, Figure 5, Figure 6, Figure 7, Figure 8, Figure 9, Figure 10, Figure 11, Figure 12, Figure 13, Figure 14, Figure 15, Figure 16 and Figure 17

The distinguishing characteristics of this species were provided by Martynov et al. [3], who also included figures of the nymph. Herein is a complementary description including variable characteristics of abdominal terga, the posterior margin of sternum IX, and illustrations useful for species identification (Figure 1, Figure 2, Figure 3, Figure 4, Figure 5 and Figure 6). In addition, characteristics of the winged stage (Figure 7, Figure 8, Figure 9, Figure 10, Figure 11, Figure 12, Figure 13, Figure 14, Figure 15 and Figure 16) and egg (Figure 17) are described for the first time.

**Material examined.** 10 nymphs, 28.IV.2024, coll. Yi-Fei Feng; 1 male imago, 1 female imago, 2 male subimagoes, and 2 female subimagoes, 4.V.2023, coll. Xian-Fu Li; 6 nymphs, 6 male imagoes, 5 female imagoes, 4 male subimagoes, and 4 female subimagoes, 21.V.2025, coll. Xian-Fu Li; China, Yunnan Province, Dali City, Mt. Cangshan, Stream Qingbi, 25°39′08.6″ N 100°9′27.3″ E, 2200 m a.s.l., 2 female imagoes, 13.VI.2022; 6 nymphs, 2 male imagoes, 3 female imagoes, 1 male subimago, and 1 female subimago, 15.VI.2022; 6 nymphs, 3 male imagoes, 4 female imagoes, 1 male subimago, and 1 female subimago, 16.VI.2022; 1 male imago, 1 female imago, and 1 male subimago, 17.VI.2022; China, Yunnan Province, Shangri-La City, Jiantang Town, Dugang River, 27°47′50.4″ N, 99°48′43.3″ E, 3361 m a.s.l., 12.VI.2022, coll. Xian-Fu Li.

**Diagnoses.** The species is morphologically similar to *C. colossa* and *C. corpulenta* (Braasch, 1981), each of which has the posteromedian abdominal tergal projections relatively weakly developed [3,14]; this distinguishes them from other species of the *C. nigra* complex. The three species *C. wangi*, *C. colossa*, and *C. corpulenta* can be distinguished from one another by features of the labrum, mesothorax, tarsal claw, and abdominal terga. The labrum of *C. colossa* has no obvious anteromedian emargination, and its tarsal claw has two denticles [14]. In contrast, *C. corpulenta* and *C. wangi* both have a deep anteromedian emargination of the labrum, and their tarsal claws have just one denticle [3]. Anterolateral projections of the mesothorax of *C. corpulenta* are poorly developed, and projections on terga VI–VIII are most prominent, while anterolateral projections of the mesothorax of *C. wangi* are well-developed, and projections on terga V–VII are most prominent [3].

**Figure 1:**
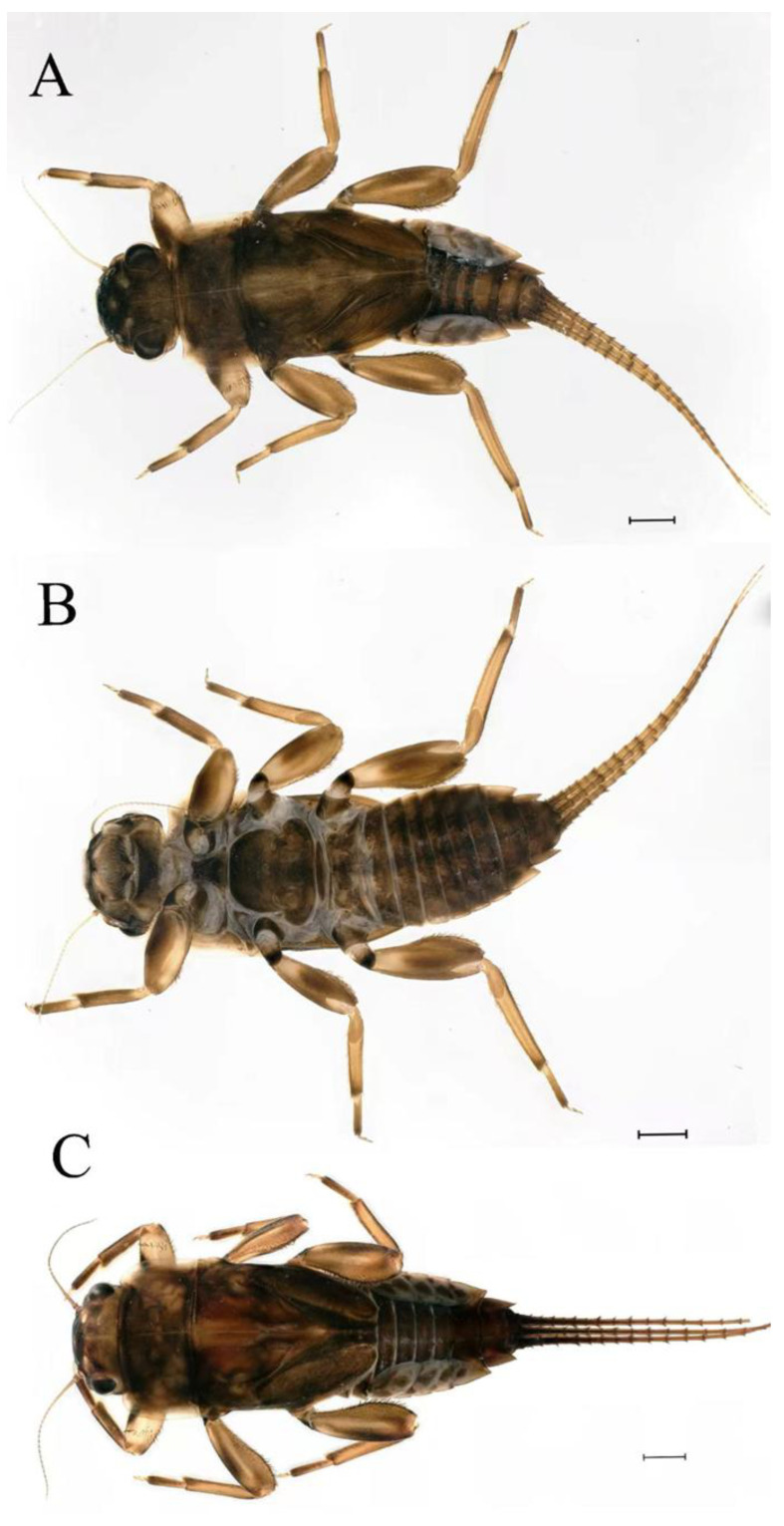
Final nymphal instar of *Cincticostella wangi*: (**A**) dorsal habitus of male; (**B**) dorsal habitus of male; (**C**) ventral habitus of female. Scale bar: 1 mm.

**Descriptions.** Complementary description of ***final nymphal instar*** (Figure 1A–C). Head width, male 2.32–2.38 mm, female 2.22–2.65 mm; body length (excluding tails), male 9.08–11.49 mm, female 8.67–11.63 mm; cerci length, male 4.05–6.12 mm, female 5.29–5.96 mm, middle caudal filament, male 5.34–6.18 mm, female 4.93–7.76 mm.

**Figure 2:**
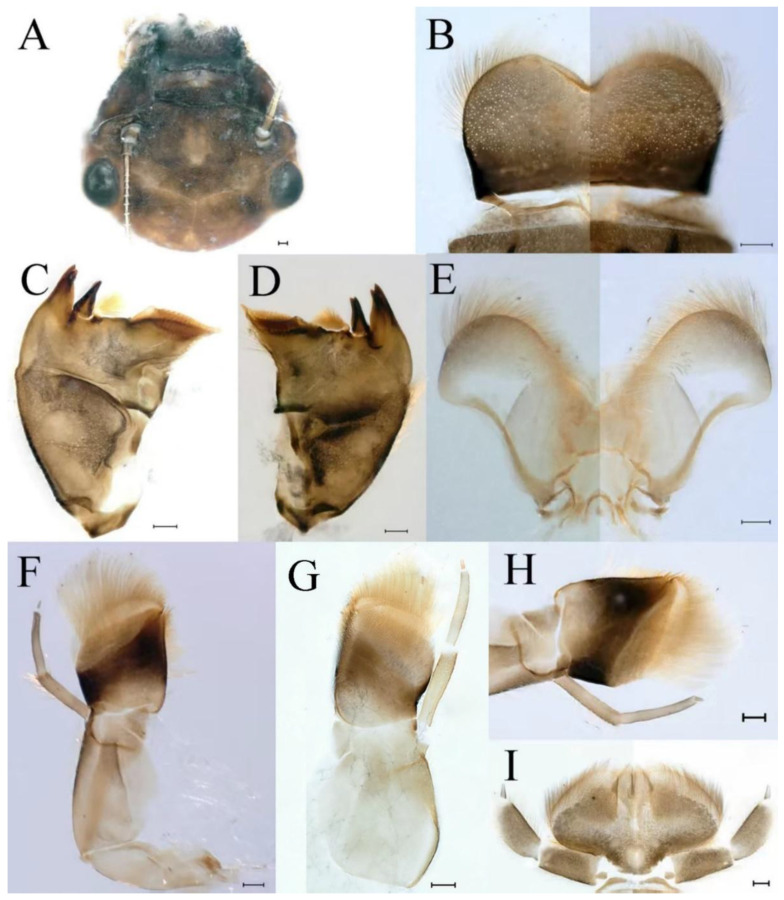
Nymphs of *Cincticostella wangi*: (**A**) head; (**B**) labrum; (**C**) left mandible; (**D**) right mandible; (**E**) hypopharynx; (**F**) right maxilla (ventral view); (**G**) right maxilla (dorsal view); (**H**) apex of left maxilla (ventral view); (**I**) labium. Scale bar: 0.1 mm.

**Head.** Brown without tubercles, prominent bright ocelli (Figure 1A,C and Figure 2A). Genae moderately developed (Figure 2A). Antennae with setae on articulations, antennae 1.2 times longer than head length. Morphology and structure of mouth parts (Figure 2B–I) similar to specimens previously described from India–China border region, but ratio of maximum width of emargination to maximum width of labrum = 1: 1.9 (Figure 2B). Maxillary palp long (0.69 mm), segment length ratio (from basal to apical) = 6: 6: 1 (Figure 2F–H). Labial elliptical glossae elliptical and almost 2.1 times longer than wide (Figure 2I). Segment III of labial palp 2.0 times longer than wide at base (Figure 2I).

**Figure 3:**
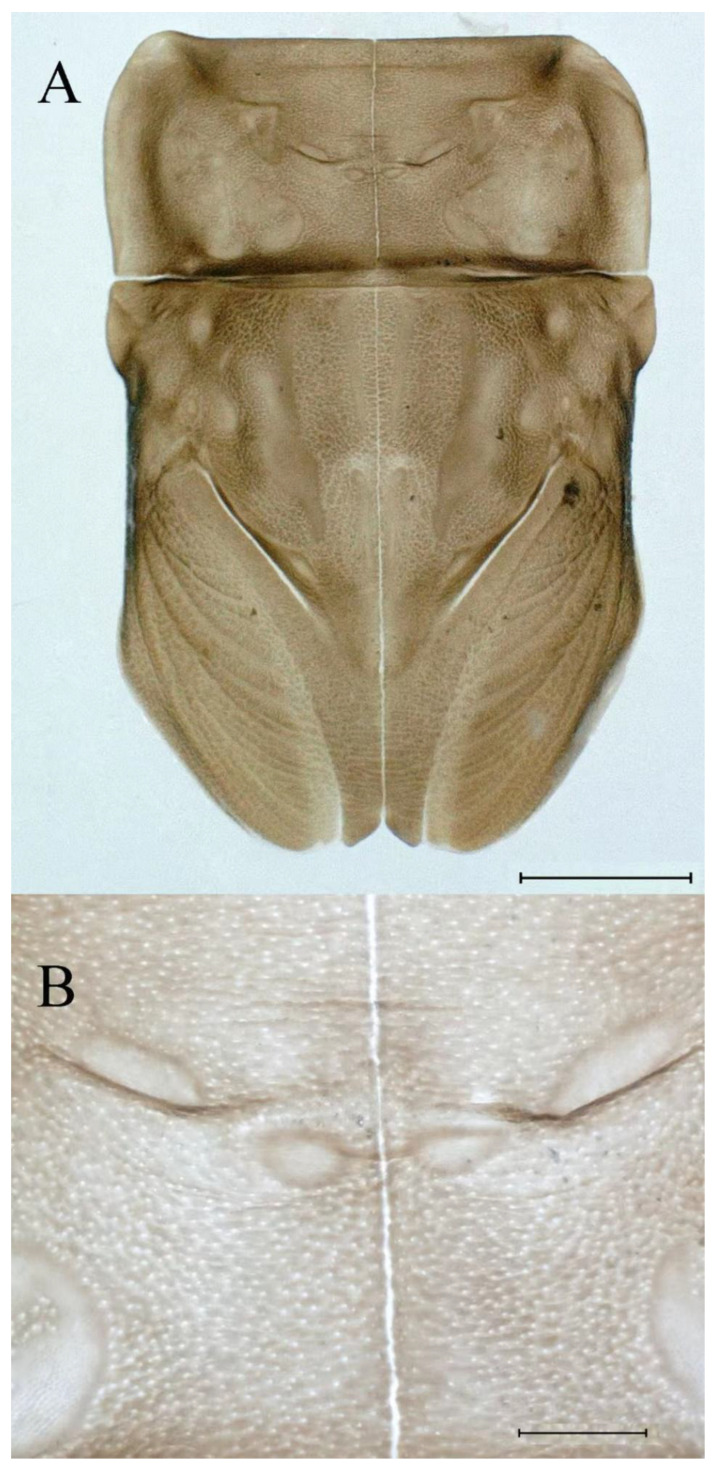
Nymphs of *Cincticostella wangi*: (**A**) thorax of last nymphal instar; (**B**) pronotum of last nymphal instar enlarged. Scale bar: (**A**) = 1 mm; (**B**) = 0.1 mm.

**Figure 4:**
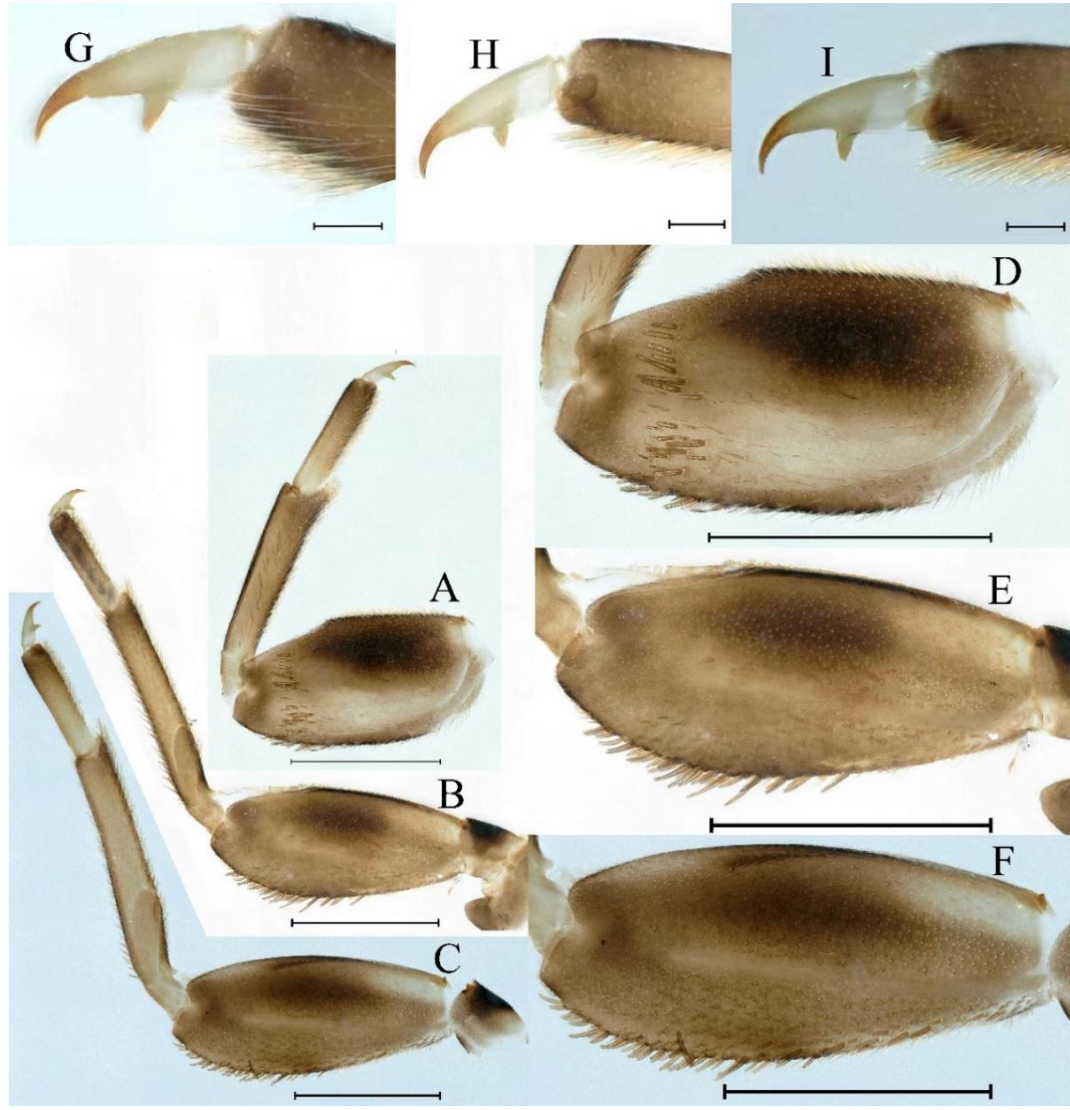
Nymphs of *Cincticostella wangi*: (**A**) foreleg; (**B**) midleg; (**C**) hindleg; (**D**) fore femur; (**E**) middle femur; (**F**) hind femur; (**G**) claw of foreleg; (**H**) claw of midleg; (**I**) claw of hindleg. Scale bar: (**A**–**F**) = 1 mm; (**G**–**I**) = 0.1 mm.

**Thorax.** Pronotum expanded laterally, with anterolateral angles small and projecting forward; mesonotum projections moderately developed, rounded, their outer margins not notched (Figure 1A,C and Figure 3A). Dorsal surface of thorax without setae (Figure 3B). All femora slightly fattened (length/width ratio = fore femur 1.9; middle femur 2.3; hind femur 2.2) (Figure 4A–F), each one with longitudinal ridge. Length ratio of leg segments (femora: tibia: tarsi): foreleg 2.0: 1.8: 1.0; middle leg 2.1: 2.0: 1.0; hindleg 2.8: 2.0: 1.0. All femora outer margins without apical projections, any distinct serration also absent (Figure 4A–F). Fore femora moderately dilated, ventral margin smooth without setae, dorsal margin with long, stout and thin, hair-like setae (Figure 4D), with distinct transverse band of numerous, mainly very long to middle-sized, stout setae with deeply bifurcated apices. Dorsal surfaces of middle and hind femora covered with numerous mainly middle sized and short, rounded or bifurcated apically, stout setae (Figure 4E,F). Outer margins of middle and hind femora covered with long, bifurcated stout setae; basal half of outer margins also with long, stout, hair-like setae. Inner margins of middle and hind femora without stout setae, only with scattered hair-like setae (Figure 4A–C). Claws of all legs with one denticle each and without subapical setae (Figure 4G–I).

**Figure 5:**
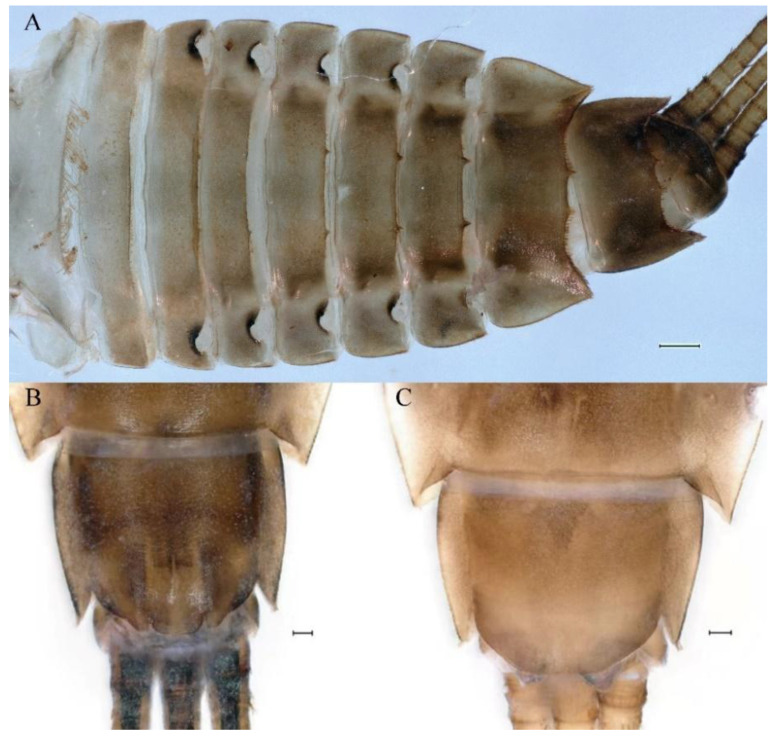
Nymphs of *Cincticostella wangi*: (**A**) abdomen (dorsal view); (**B**) posterior part of abdomen of male (ventral view); (**C**) posterior part of abdomen of female (ventral view). Scale bar: (**A**) = 1 mm; (**B**,**C**) = 0.1 mm.

**Figure 6:**
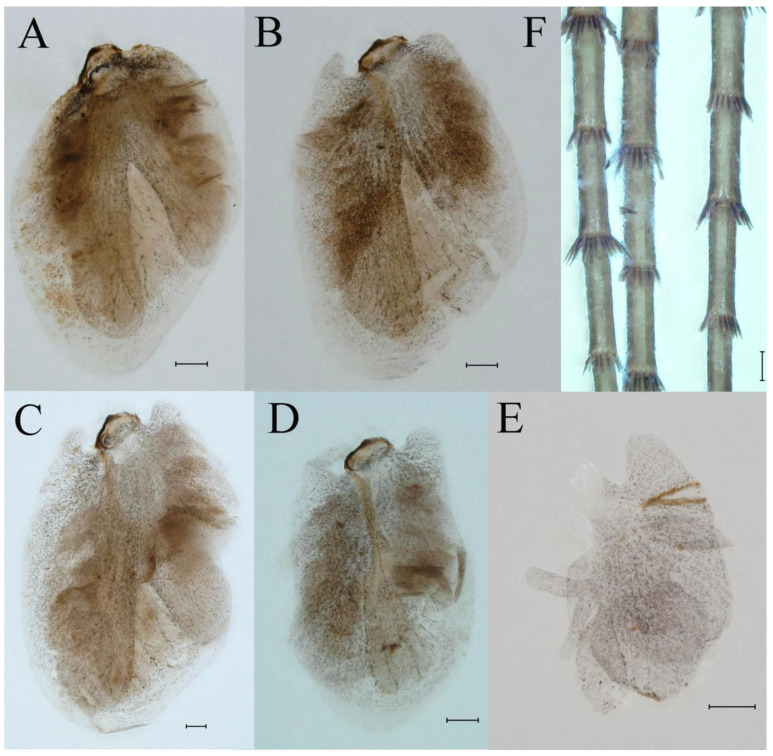
Nymphs of *Cincticostella wangi*: (**A**) gill III; (**B**) gill IV; (**C**) gill V; (**D**) gill VI; (**E**) gill VII; (**F**) caudal filaments. Scale bar: 0.1 mm.

**Abdomen**. Posterior margin of tergum I with thin and stout, hair-like setae (Figure 5A). Paired posteromedian projections present on abdominal terga III–IX, but protuberances of terga III, IV very small, VIII–IX round (Figure 5A). Dorsal surfaces of terga above projections and surfaces of projections covered with short stout setae (Figure 5A). Posterior margins of terga VIII–IX in submedian areas covered with rows of elongated, apically rounded, stout setae (Figure 5A); posterior margin of tergum X with discontinuous row of stout setae. Abdominal segments VII–IX with posterolateral projections (Figure 5A). Posterior margin of sternum IX of male round (Figure 5B); posterior margin of sternum IX of female almost straight (Figure 5C). Shapes of gills as in Figure 6A–E, and characteristics of caudal filaments as in Figure 6F.

**Figure 7:**
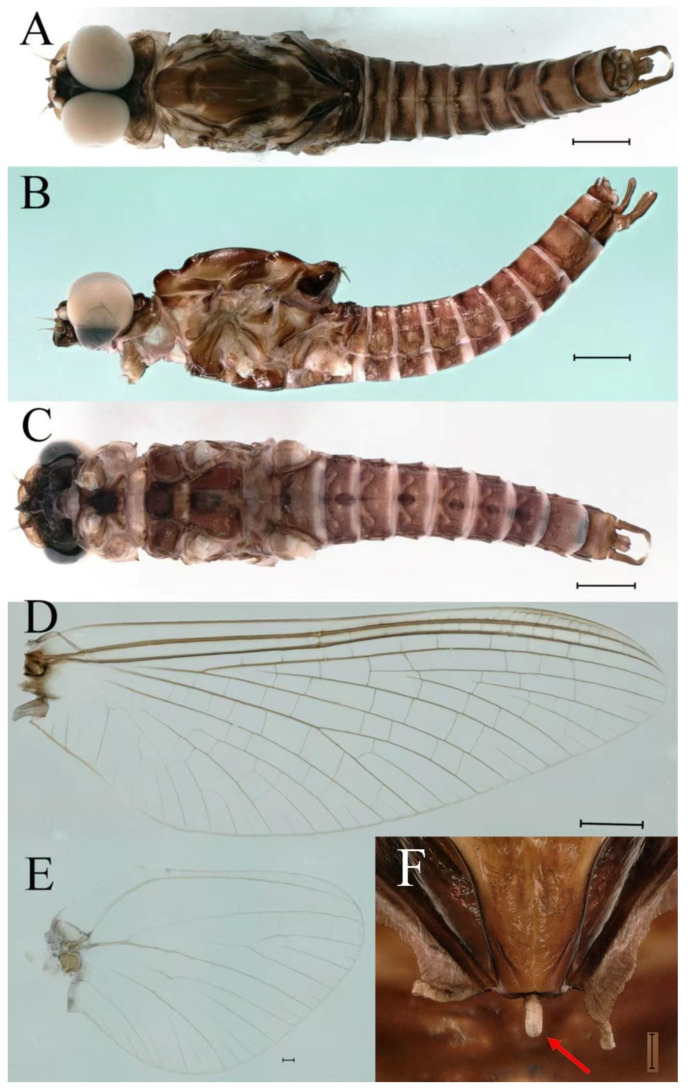
Male imago of *Cincticostella wangi*: (**A**) dorsal view of body; (**B**) lateral view of body; (**C**) ventral view of body; (**D**) forewing; (**E**) hindwing; (**F**) lateral scutellar projections, middle one indicated by red arrow. Scale bar: (**A**–**D**) = 1 mm (**A**–**D**); (**E**,**F**) = 0.1 mm.

***Male imago***. Body length 9.92–11.06 mm (excluding tails), head width 2.07–2.29 mm, forewing length 11.1–12.01 mm, hindwing length 2.41–2.83 mm, cerci length 13.12–13.8 mm, middle caudal filament 11.79–12.86 mm. Body color brown to dark brown (Figure 7A–C and Figure 16A).

**Head.** Compound eyes separate, upper portion brown and lower portion black (Figure 7B).

**Figure 8:**
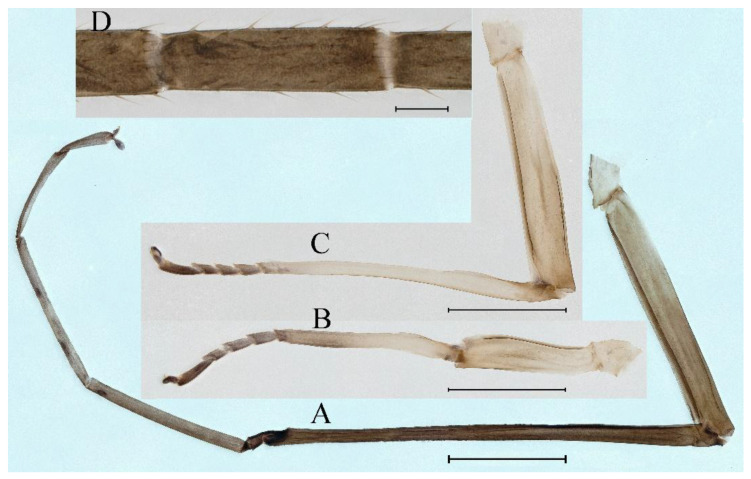
Male imago of *Cincticostella wangi*: (**A**) foreleg; (**B**) midleg; (**C**) hindleg; (**D**) caudal filament enlarged. Scale bar: (**A**–**C**) = 1 mm; (**D**) = 0.1 mm.

**Thorax**. Pronotum with expanded posterolateral sac-like structure (Figure 7A–C). Prosternum dark brown, with slightly anteriorly converging longitudinal carinae, maximum width between carinae 3.0 times minimum width (Figure 7C). Basisternum of mesosternum dark brown, with parallel furcasternum (Figure 7C). Mesonotum with three projections on posterior margin, middle projection short (Figure 7F, indicated by red arrow). Forewing hyaline (Figure 7D), but C and Sc regions semihyaline; longitudinal veins dark brown and cross veins light brown to semihyaline; cross veins in stigma region between C and Sc separated into two parts by long vein; Rs leaves MA at very base, MA forked two-thirds of distance from base to margin; MP forked slightly more distal than fork of MA + Rs; CuP recurved. Hindwing totally hyaline (Figure 7E), costal projection small, rounded, located at distance one-third from base to apex; MP forked between forks of R1 + MA and MA. Forelegs brown to dark brown (Figure 8A), mid- and hindlegs brown (Figure 8B,C). Femur:tibia:tarsus of foreleg = 1.0:1.7:1.8, tarsal segments from basal to apical = 1.0:5.3:5.1:3.0:1.6. Femur:tibia:tarsus of midleg = 1.6:1.7:1.0, tarsal segments from basal to apical = 1.0:1.9:1.6:1.6:2.6. Femur:tibia:tarsus of hindleg = 2.2:2.4:1.0, tarsal segments from basal to apical = 1.0:1.4:1.2:1.2:2.4. Claws of all legs similar, one blunt and one hooked.

**Abdomen.** Terga III–X each with pale median line on dorsal surface (Figure 7A). Terga I–IX each with pale stripe on posterior margin (Figure 7A,B). Terga I, II, IV–VII each with big dark brown spots, and terga II–VIII each with symmetric pale stripe and small spots on anterior part on ventral surface (Figure 7C). All terga without posterolateral spines, excluding terga VIII and IX (Figure 7A,C). Caudal filaments dark brown, covered with spines (Figure 8D).

**Figure 9:**
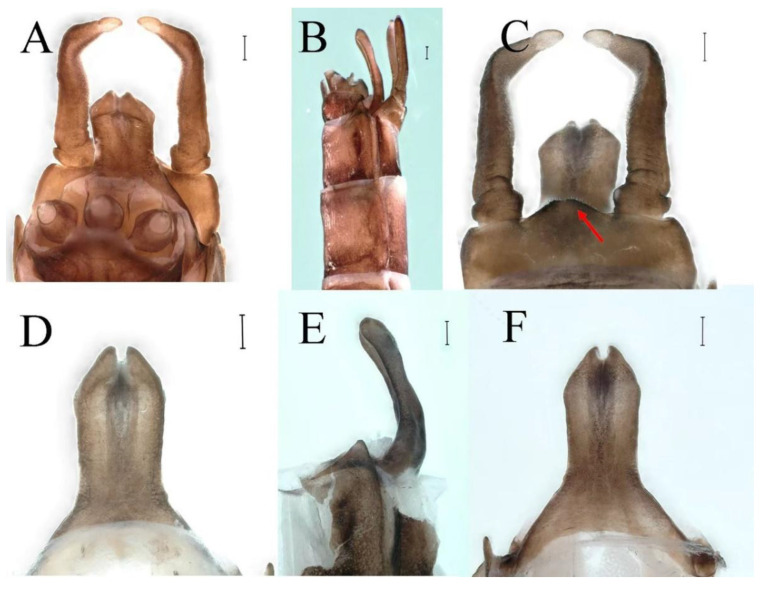
Male imago of *Cincticostella wangi*: (**A**) genitalia (dorsal view); (**B**) genitalia (lateral view); (**C**) genitalia (ventral view), median convex lobe indicated by red arrow; (**D**) penes (dorsal view); (**E**) penes (lateral view); (**F**) penes (ventral view). Scale bar: 0.1 mm.

**Genitalia.** Styliger plate with median convex lobe-like trapezium (Figure 9C, indicated by red arrow). Second segment of forceps slightly constricted at point in apical fourth; segment 3 oval (Figure 9A,C). Penis lobes (Figure 9A–F) compact and smooth, pointed at apex and slightly subapically swollen in ventral view (Figure 9C,F); penis lobes with linear groove on apical half in dorsal view (Figure 9A,B,D,E), lobes separated by round medial cleft at apex, ratio of apicomedial length of round cleft to maximum lobes length = 1:6.3 (Figure 9C,F).

**Figure 10:**
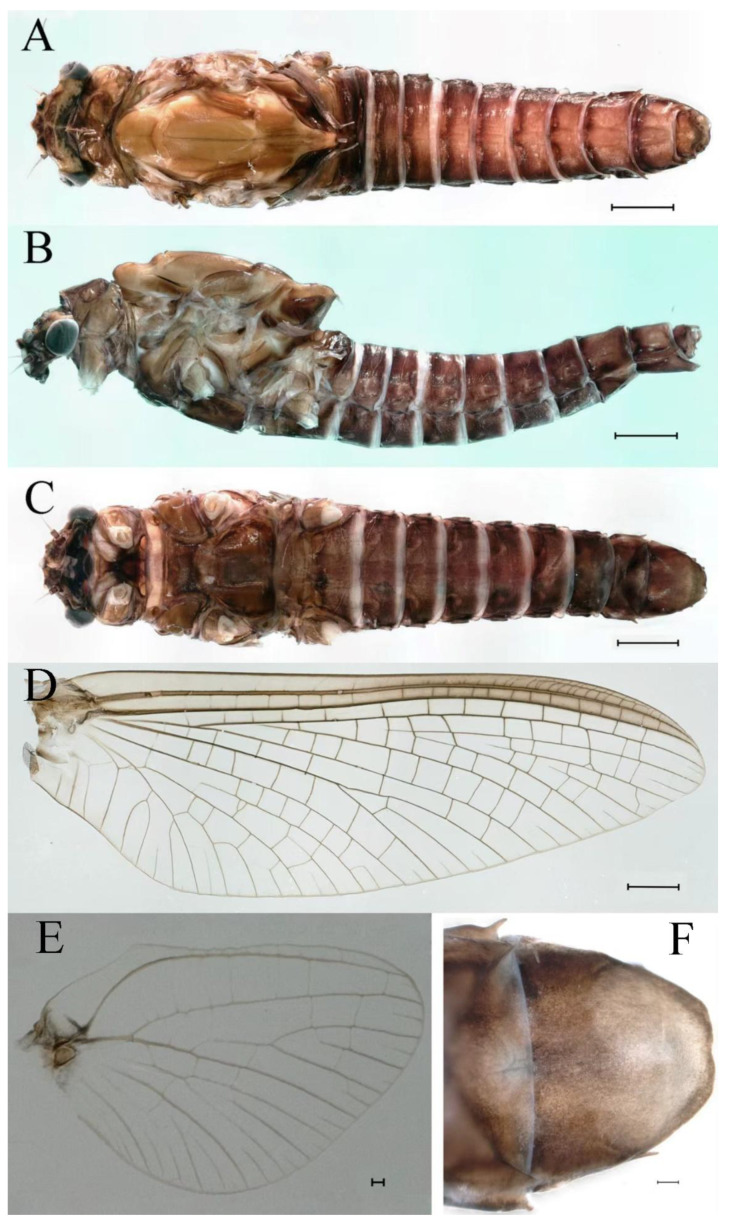
Female imago of *Cincticostella wangi*: (**A**) dorsal view; (**B**) lateral view; (**C**) ventral view; (**D**) forewing; (**E**) hindwing; (**F**) posterior part of abdomen (ventral view). Scale bar: (**A**–**D**) = 1 mm; (**E**,**F**) = 0.1 mm.

**Figure 11:**
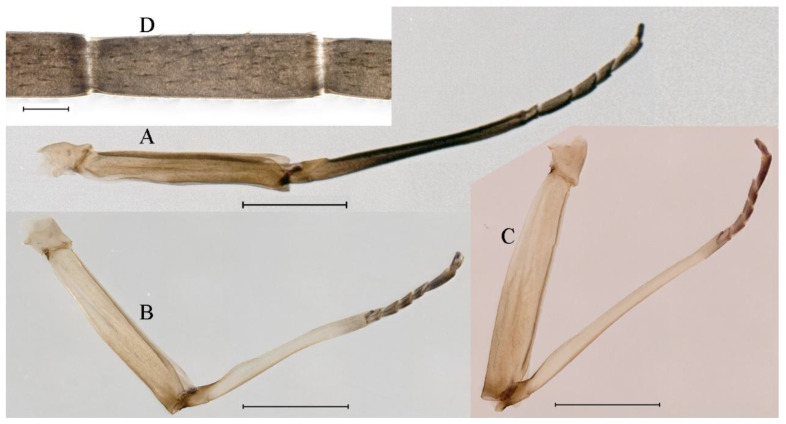
Female imago of *Cincticostella wangi*: (**A**) foreleg; (**B**) midleg; (**C**) hindleg; (**D**) caudal filament enlarged. Scale bar: (**A**–**C**) = 1 mm; **D** = 0.1 mm.

***Female imago***. Color pattern similar to male (Figure 10A–C and Figure 16B); body length 9.91–11.24 mm (excluding tails), head width 1.89–2.15 mm, cerci length 11.84–13.46 mm, middle caudal filament 12.02–12.5 mm, forewing 12.09–12.46 mm (Figure 10A), hindwing 3.01–3.16 mm (Figure 10B). Lengths of femur (Figure 11A):tibia:tarsus of foreleg = 1.4:1.7:1.0, tarsal segments from basal to apical = 1.0:2.3:1.8:1.2:2.0; femur: tibia: tarsus of midleg.(Figure 11B) = 2.1:2.1:1.0, tarsal segments from basal to apical = 1.1:1.3:1.2:1.0:2.2; femur:tibia:tarsus of hindleg (Figure 11C) = 2.7:3.4:1.0, tarsal segments from basal to apical = 1.0:1.2:1.2:1.1:2.4. Posterior margin of subgenital plate produced to one-fifth length of sternum VIII. Posterior margin of subanal plate concave (Figure 10F). Color pattern of caudal filaments similar to male (Figure 11D).

**Figure 12:**
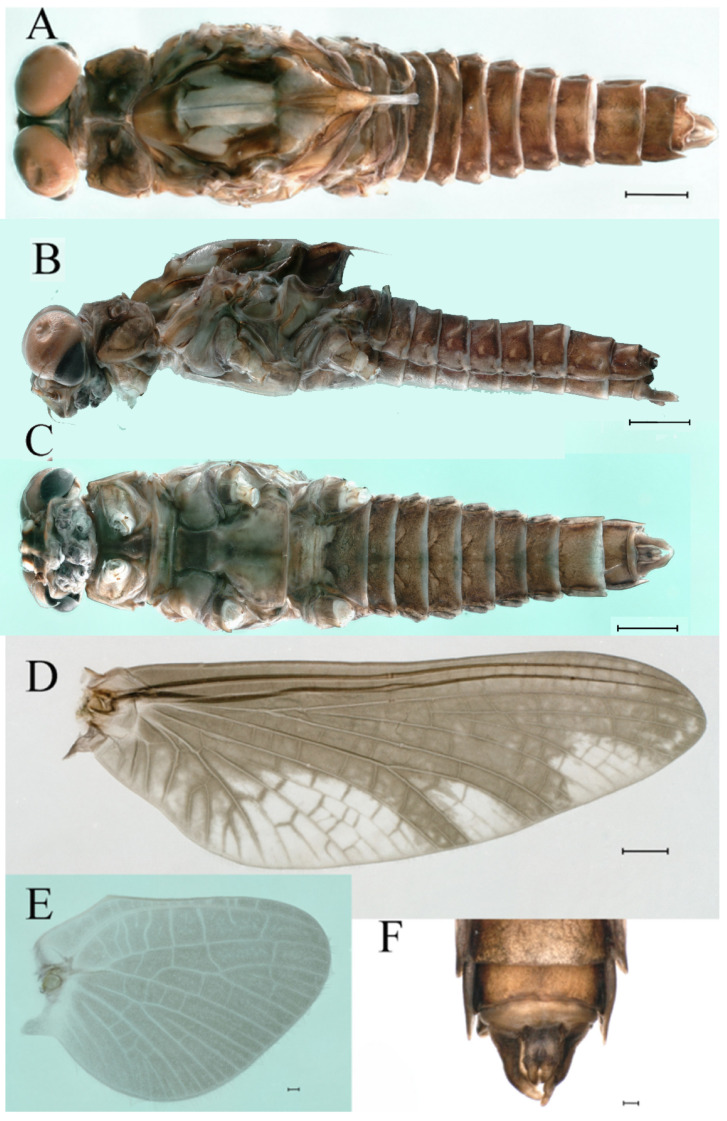
Male subimago of *Cincticostella wangi*: (**A**) dorsal view; (**B**) lateral view; (**C**) ventral view; (**D**) forewing; (**E**) hindwing; (**F**) ventral view of genitalia. Scale bar: (**A**–**D**) = 1 mm; (**E**,**F**) = 0.1 mm.

**Figure 13:**
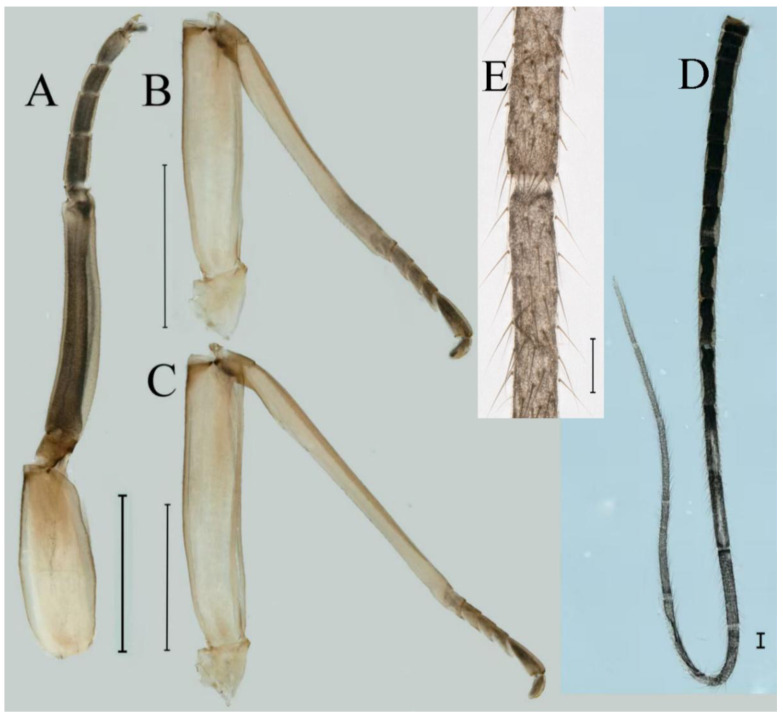
Male subimago of *Cincticostella wangi*: (**A**) foreleg; (**B**) midleg; (**C**) hindleg; (**D**) caudal filament; (**E**) caudal filament enlarged. Scale bar: (**A**–**C**) = 1 mm; 0.1 mm.

***Male subimago*.** Body color brown (Figure 12A–C and Figure 16C). Forewing taupe to black brown with crossveins infuscated (Figure 12D and Figure 16C), hindwing taupe to black brown without crossvein infuscation (Figure 12E and Figure 16C). Mesonotum with three projections on posterior margin, middle projection longest (Figure 12A). Penes as in Figure 12F. Body length 9.3–11.85 mm (excluding tails), head width 2.26–2.8 mm, cerci length 7.61–11.08 mm, middle caudal filament 6.77–8.79 mm, forewing 11.39–13.81 mm, hindwing 2.74–2.92 mm. Margins of femur, tibia, and tarsus of foreleg, midleg, and hindleg densely covered with spines (Figure 13A–C). Length of femur:tibia:tarsus of foreleg = 1.1:1.4:1.0, tarsal segments from basal to apical = 1.0:3.3:2.9:1.9:2.6; femur:tibia:tarsus of midleg = 1.5:1.6:1.0, tarsal segments from basal to apical = 1.0:2.1:2.0:1.6:2.6; femur:tibia:tarsus of hindleg = 2.3:2.6:1.0, tarsal segments from basal to apical = 1.1:1.2:1.1:1.0:2.2. Caudal filaments brown, relatively densely covered with long spines (Figure 13D,E).

**Figure 14:**
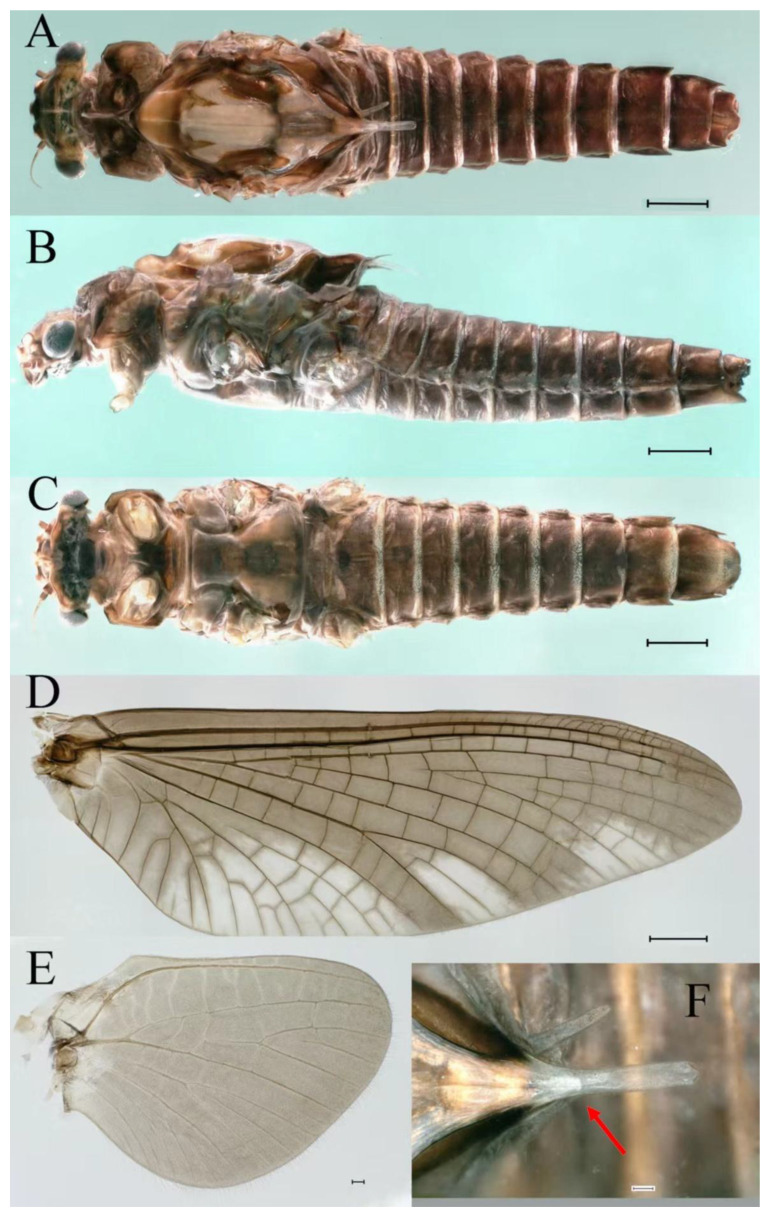
Female subimago of *Cincticostella wangi*: (**A**) dorsal view of body; (**B**) lateral view of body; (**C**) ventral view of body; (**D**) forewing; (**E**) hindwing; (**F**) lateral scutellar projections, middle one indicated by red arrow. Scale bar: (**A**–**D**) = 1 mm; (**E**,**F**) = 0.1 mm.

**Figure 15:**
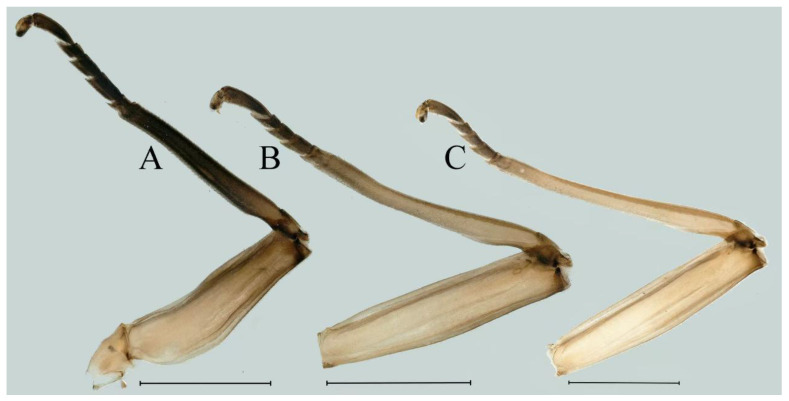
Female subimago of *Cincticostella wangi*: (**A**) foreleg; (**B**) midleg; (**C**) hindleg. Scale bar: 1 mm.

**Figure 16:**
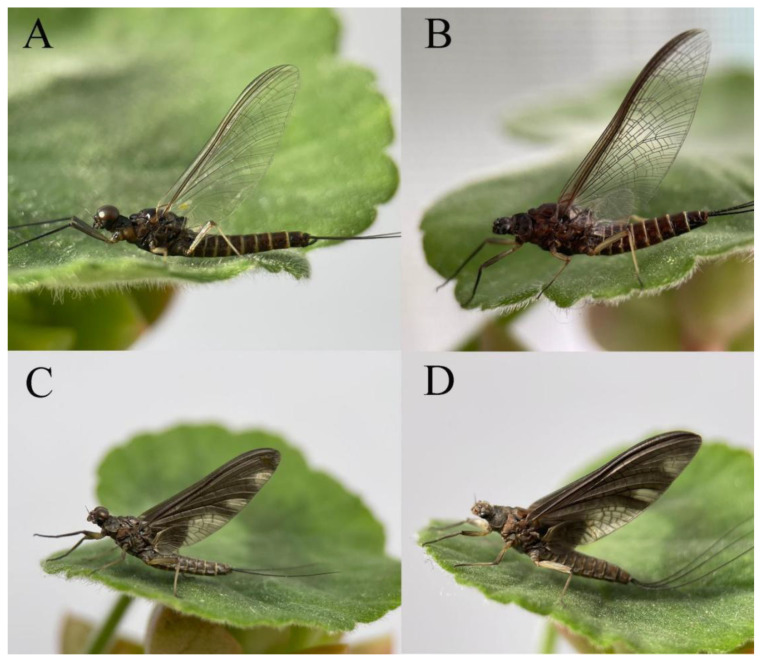
Winged stages of *Cincticostella wangi* (living): (**A**) male imago; (**B**) female imago; (**C**) male subimago; (**D**) female subimago.

***Female subimago***. Similar to male subimago except for usual sexual differences (Figure 14A–F and Figure 16D). Head width 1.94–2.16 mm, body length 9.35–11.12 mm (excluding tails), forewing length 11.71–11.93 mm, hindwing length 2.7–3.1 mm, cerci length 8.24–10.3 mm, middle caudal filament 8.6–10.71 mm. Length of femur:tibia:tarsus of foreleg (Figure 15A) = 1.8:2.0:1.0, tarsal segments from basal to apical = 1.0:1.6:1.2:1.0:2.3; femur:tibia:tarsus of midleg (Figure 15B) = 1.2:1.3:1.0, tarsal segments from basal to apical = 1.1:1.3:1.1:1.0:2.8; femur:tibia:tarsus of hindleg (Figure 15C) = 1.9:2.1:1.0, tarsal segments from basal to apical = 1.0:1.1:1.6:1.2:3.4.

**Figure 17:**
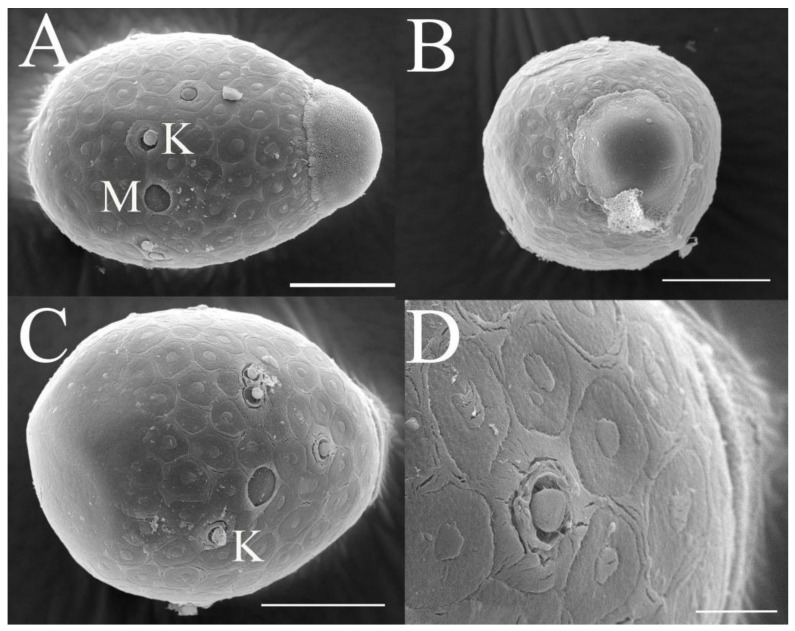
Egg of *Cincticostella wangi*: (**A**) lateral view with micropyle (M) and knob of attachment structure (K); (**B**) polar cap; (**C**) bottom view; (**D**) knob of attachment structure (K) enlarged. Scale bar: (**A**–**C**) = 0.05 mm; (**D**) = 0.01 mm.

***Eggs***. Dissected from female imago. Length 159–172 μm, width 110–117 μm. Ovoid with polar cap composed of dense filaments, each filament with intumescent terminal (Figure 17A,B). Chorion with irregular hexagonal strands; mesh with one big tubercle medially; micropyle and knobs of attachment structure distributed near equator (Figure 17A–D).

**Remarks.** We examined specimens of *C. wangi* from different altitudes, and we found that, while some characters and behavior of the nymphs may vary, from altitude 2221 m to altitude 3361 m in Yunnan Province, the characters of its imago tend to be very stable. Specimens from Shangri-La City, Jiantang Town, Dugang River, 3361 m a.s.l. (where *Siphlonurus dongxi* Li & Tong was described [22]) have posterior margins of terga VIII and IX almost straight, protuberances of terga VIII very small and smooth, and terga IX with-out projections, like those (Martynov et al.: figure 22D,F) [3] from the India–China border region. However, specimens from Dali City, Mt. Cangshan, Qingbi Stream, 2221 m a.s.l. (where *Ameletus daliensis* Tong, 2021, *Notacanthella jinwu* Li & Jacobus, 2022, and *Nigrobaetis bilongus* Li, Shi, Li & Tong, 2023 were described [20,21,29]) have posterior margins of terga VIII and IX wavy with paired, blunt projections. Some nymphs from the high altitude location (Shangri-La City) have a white median line along the body. The winged stages of *C. wangi* were collected from Dali City in early March to April and from Shangri-La City in June.

### 3.2. Cincticostella funki Martynov, Selvakumar, Palatov & Vasanth, 2021

*Cincticostella funki*: Martynov et al.: figures 9–12 (nymph) [3]. Holotype and paratypes: nymphs, from India–China border region.

Figure 18, Figure 19, Figure 20, Figure 21, Figure 22, Figure 23, Figure 24, Figure 25, Figure 26, Figure 27, Figure 28, Figure 29, Figure 30, Figure 31, Figure 32, Figure 33 and Figure 34

The distinguishing characteristics of this species were provided by Martynov et al. [3], and they provided figures of the nymph. Herein is a complementary description of some characteristics including posterior margin of sternum IX and illustrations useful for species identification. In addition, the characteristics of the egg and winged stages are described for the first time.

**Material examined.** 30 nymphs, 9.III.2024; 6 nymphs and 2 female imagoes, 27.III.2024; 1 male imago, 2 female imagoes, and 1 female subimago, 29.III.2024; 2 male imagoes, 1 female imago, 2 male subimagoes, and 3 female subimagoes, 2.IV.2024; China, Yunnan Province, Tengchong City, Guangming Town, Longchuan River, 25°39′10.6″ N 98°32′12.1″ E, 1853 m a.s.l., coll. Yan-Chang Zi and Xian-Fu Li. 20 nymphs, 23.II.2024; 4 female imagoes and 9 male imagoes, 2.III.2024; 1 male subimago, 1 female subimago, 10.III.2024; China, Yunnan Province, Jingdong County, Ju River, 24°27′13.1″ N 100°48′59.2″ E, 1171 m a.s.l., coll. Xian-Fu Li.

**Diagnoses.** The nymph of *C. funki* is similar to *C. nigra* and *C. ebura*, because these nymphs all have a white median line along the body that can be used to separate them from other *Cincticostella* species. But all abdominal terga of *C. ebura* have long pairs of posteromedian projections, starting on tergum IV [4]. While abdominal terga I and X of *C. funki* and *C. nigra* have no posteromedian projections, the first appearance of relatively well-developed posteromedian projections is on tergum V [3,9]. *Cincticostella funki* can be distinguished from *C. nigra* by its claws; claws of *C. funki* have two denticles (Figure 21G,H; Martynov et al.: figure 11E [3]), or rarely three in the study of Martynov et al. [3], but claws of *C. nigra* have more than five denticles [2,3,9,10], such as Allen: figure 6 [2]. In addition, the penes of *C. funki* and *C. nigra* have significant differences. The penis lobes of *C. funki* are separated by rectangular cleft at point, with an inverted triangular groove on the apical half in both dorsal and ventral views (Figure 27A–C), while those of *C. nigra* are pointed at apex and with a subapical swelling (Allen: figure 1) [2].

**Figure 18:**
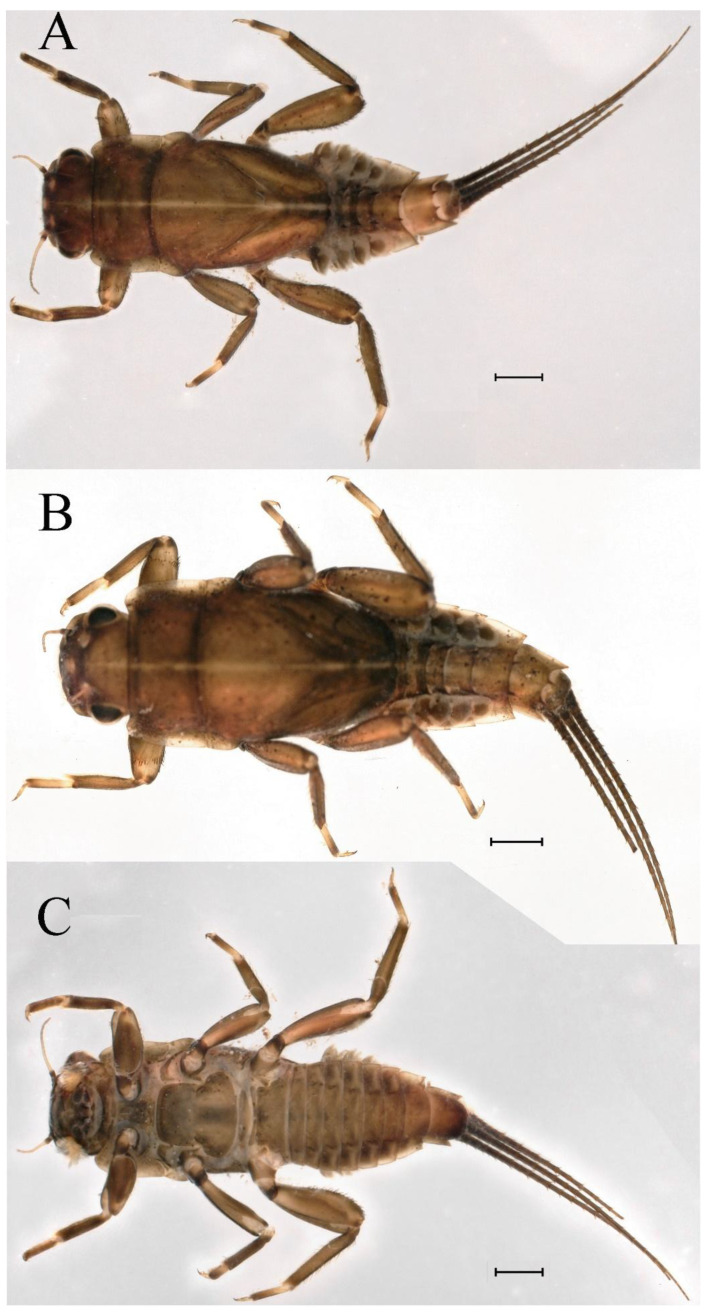
Last nymphal instar of *Cincticostella funki*: (**A**) dorsal habitus of male; (**B**) dorsal habitus of female; (**C**) ventral habitus of male. Scale bar: 1 mm.

**Descriptions.** Complementary description of ***final nymphal instar***. Head width, male 2.14–2.48 mm, female 2.20–2.36 mm; body length (excluding tails), male 8.98–12.53 mm, female 9.62–10.93 mm; cerci length, male 6.05–6.21 mm, female 5.82–6.85 mm, middle caudal filament, male 6.19–7.17 mm, female 4.93–7.76 mm. Body black with dorsal median pale line from head to abdominal tergum X (Figure 18A–C).

**Figure 19:**
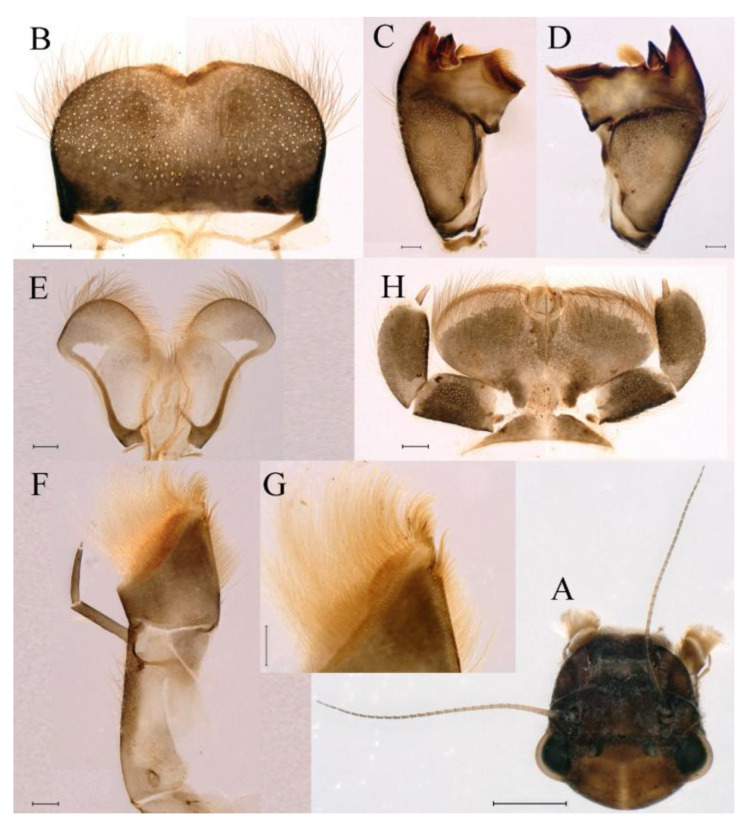
Nymphs of *Cincticostella funki*: (**A**) head; (**B**) labrum; (**C**) left mandible; (**D**) right mandible; (**E**) hypopharynx (ventral view); (**F**) left maxilla; (**G**) apex of right maxilla (ventral view); (**H**) labium (ventral view). Scale bar: (**A**) = 1 mm; (**B**–**H**) = 0.1 mm.

**Head.** The shapes of genae and mouthparts are as depicted in Figure 19A–H. Ratio of ratio of maximum width of emargination to maximum width of labrum = 1:3.0. Maxillary palpi long (0.56 mm), length ratio from basal to apical segments = 6.6:5.6:1.

**Figure 20:**
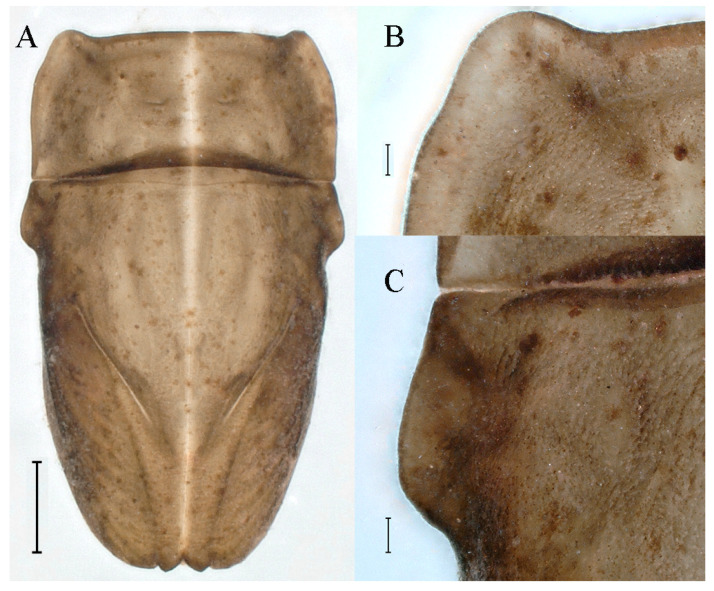
Nymphs of *Cincticostella funki*: (**A**) thorax of last nymphal instar (dorsal view); (**B**) anterolateral projections of pronotum; (**C**) anterolateral projections of mesothorax. Scale bar: (**A**) = 1 mm; (**B**,**C**) = 0.1 mm.

**Figure 21:**
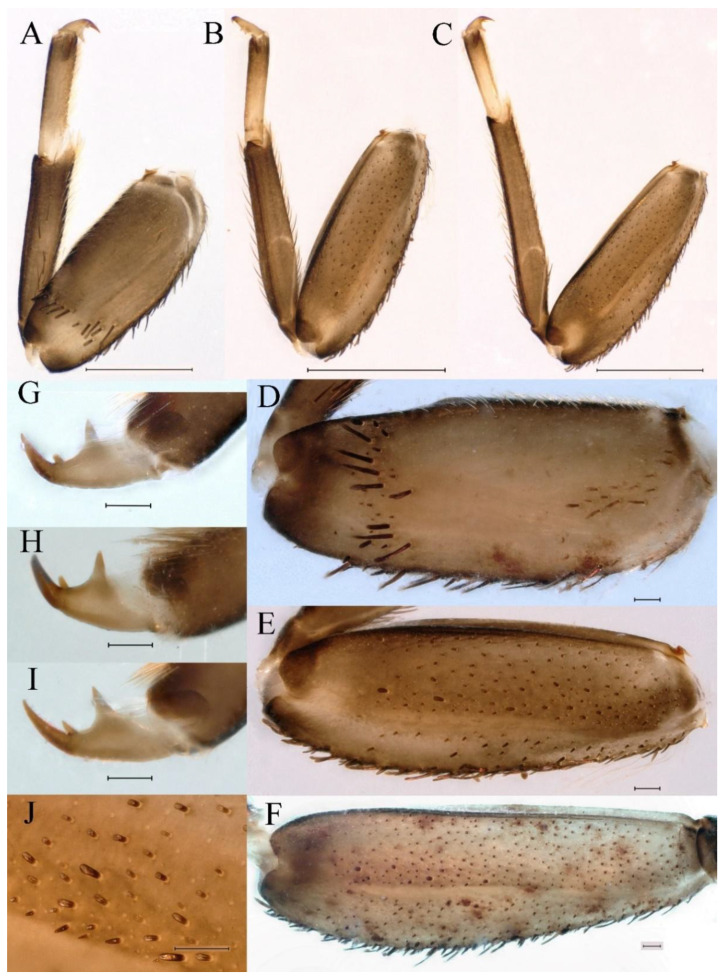
Nymphs of *Cincticostella funki*: (**A**) foreleg; (**B**) midleg; (**C**) hindleg; (**D**) fore femur; (**E**) middle femur; (**F**) hind femur; (**G**) claw of foreleg; (**H**) claw of midleg; (**I**) claw of hindleg; (**J**) setae of dorsal surface of middle femur. Scale bar: (**A**–**C**) = 1 mm; (**D**–**J**) = 0.1 mm.

**Thorax.** Black with white median line, shapes of thorax as in Figure 20A–C. All femora slightly fattened (length/width ratio = fore femur 2.4; middle femur 2.6; hind femur 3.1) (Figure 21A–F), Length ratio of leg segments (femora:tibia:tarsi): foreleg 1.0:1.3:1.1; middle leg 1.3:1.9:1.0; hindleg 1.0:1.4:1.1. Setae of dorsal surface of middle femur as in Figure 21J. Tarsal claws of all legs hooked, with two denticles relatively distant from each other (Figure 21G–I).

**Figure 22:**
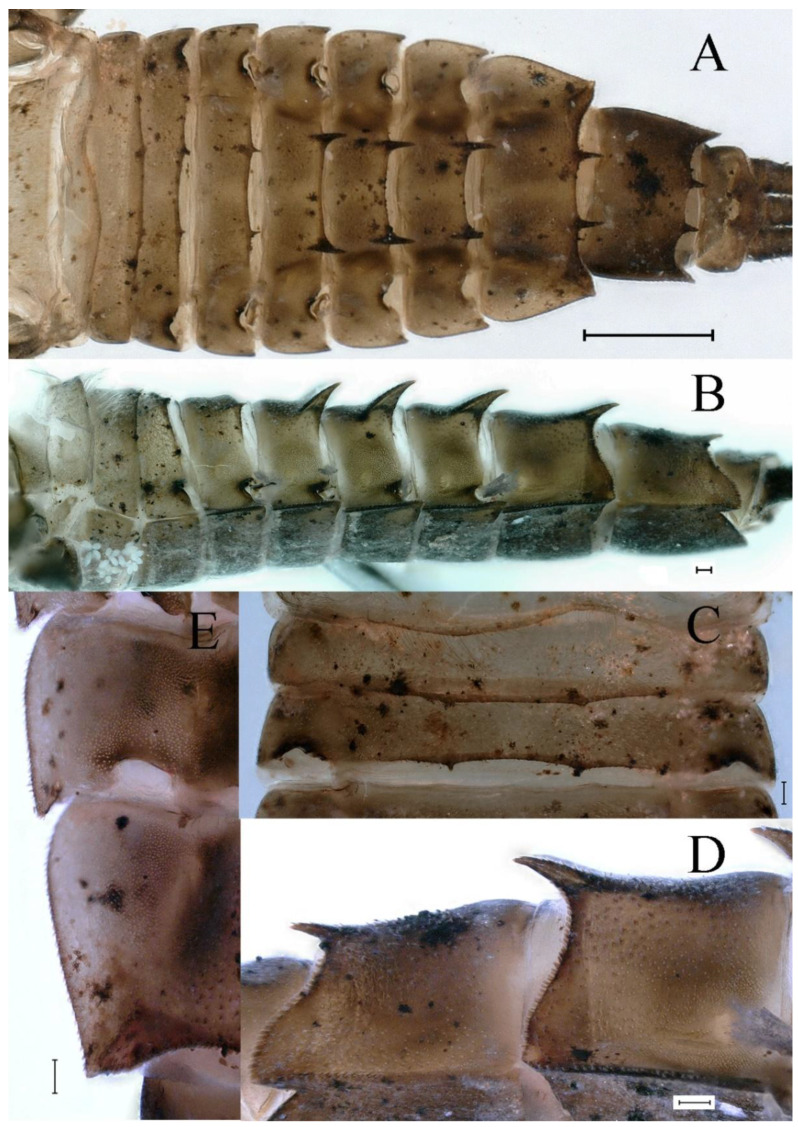
Nymphs of *Cincticostella funki*: (**A**) abdomen (dorsal view); (**B**) abdomen (lateral view); (**C**) abdominal terga I–III enlarged (dorsal view); (**D**) abdominal terga VIII–IX enlarged (lateral view); (**E**) lateral margins of abdominal terga VII, VIII. Scale bar: (**A**) = 1 mm; (**B**–**E**) = 0.1 mm.

**Figure 23:**
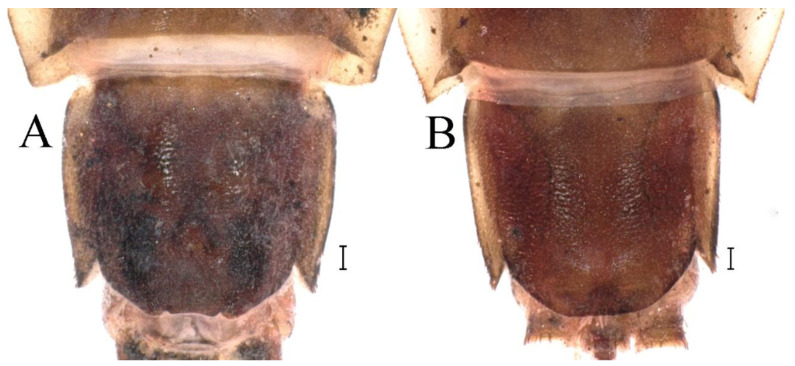
Nymphs of *Cincticostella funki*. **A** Posterior part of abdomen of male (ventral view); **B** posterior part of abdomen of female (ventral view). Scale bar: 100 μm (**A**,**B**).

**Figure 24:**
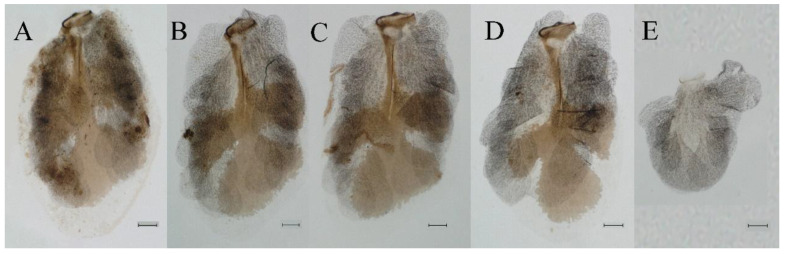
Nymphs of *Cincticostella funki*: (**A**) gill III; (**B**) gill IV; (**C**) gill V; (**D**) gill VI; (**E**) gill VII. Scale bar: 0.1 mm.

**Abdomen**. Posterior margins of terga I and II with thin and stout, hair-like setae (Figure 22B,C). Pairs of pointed, not bifurcated, projections present on abdominal terga II–IX; those on terga V–VIII strongest (Figure 22A–D). Posterior margin of tergum X with stout setae with apices rounded, without projections. Abdominal segments V–IX with posterolateral projections; those on segment IX most developed and directed backwards and laterally (Figure 22A,E). Posterior margin of sternum IX of male slightly concave (Figure 23A); posterior margin of sternum IX of female straight (Figure 23B). Shapes of gills as in Figure 24A–E, and characteristics of caudal filaments as in Figure 18A–C.

**Figure 25:**
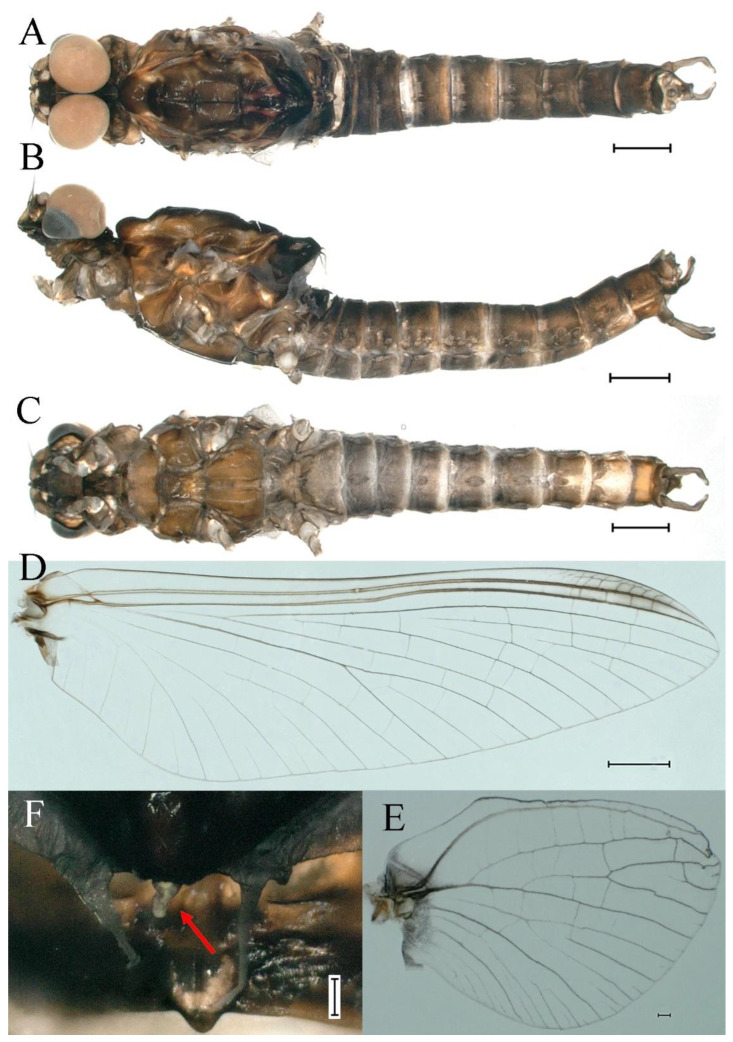
Male imago of *Cincticostella funki*: (**A**) dorsal view of body; (**B**) lateral view of body; (**C**) ventral view of body; (**D**) forewing; (**E**) hindwing; (**F**) lateral scutellar projections, middle one indicated by red arrow. Scale bar: (**A**–**D**) = 1 mm; (**E**,**F**) = 0.1 mm (**E**–**F**).

***Male imago***. Body length 10.40–12.13 mm (excluding tails), head width 1.99–2.16 mm, forewing length 12.30–13.37 mm, hindwing length 2.82–3.20 mm, cerci length 14.06–15.65 mm, middle caudal filament 12.97–14.74 mm. Body color brown to dark brown (Figure 25A–C and Figure 33A).

**Head**. Compound eyes separate, upper portion brown and lower portion black (Figure 25B).

**Figure 26:**
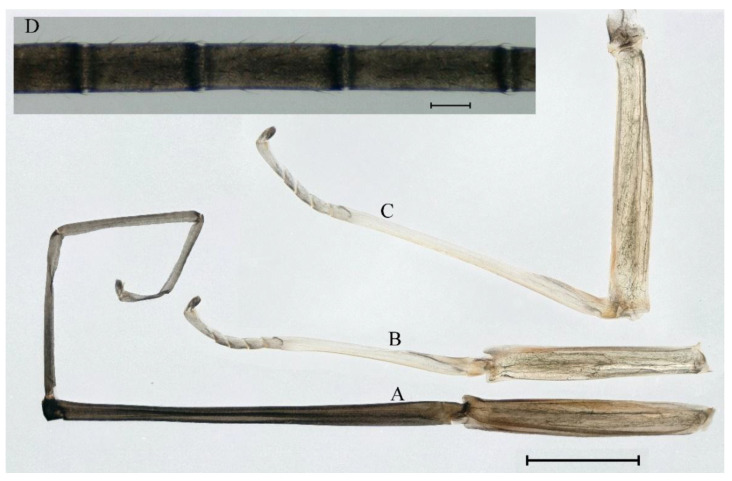
Male imago of *Cincticostella funki*: (**A**) foreleg; (**B**) midleg; (**C**) hindleg; (**D**) caudal filament. Scale bar: (**A**–**C**) = 1 mm; (**D**) = 0.1 mm.

**Thorax**. Pronotum with expanded posterolateral sac-like structure (Figure 25A,C). Prosternum dark brown, with slightly anteriorly converging longitudinal carinae, maximum width between carinae 2.3 times minimum width (Figure 25C). Basisternum of mesosternum dark brown, with parallel furcasternum (Figure 25C). Mesonotum with three projections on posterior margin, middle projection short (Figure 25F, indicated by red arrow). Forewings hyaline (Figure 25D), but C and Sc regions semihyaline; longitudinal veins dark brown and crossveins light brown to semihyaline; crossveins in stigma region between C and Sc separated into two parts by long vein; Rs leaves MA at subbasal part, MA forked three-fifths of distance from base to margin; MP and stem of MA+Rs forked equidistant from base of wings to margin; CuP recurved slightly. Hindwing totally hyaline (Figure 25E), costal projection small, rounded, located one-third from base to apex; MP forked between forks of R1 + MA and MA. Forelegs brown to dark brown (Figure 26A), mid- and hindlegs yellow brown (Figure 26B,C). Femur:tibia:tarsus of foreleg = 1.0:1.6:2.0, tarsal segments from basal to apical = 1.0:6.2:5.4:3.4:1.9; femur:tibia:tarsus of midleg = 2.1:2.1:1.0, tarsal segments from basal to apical = 1.1:1.2:1.1:1.0:2.4; femur:tibia:tarsus of hindleg = 2.3:2.5:1.0, tarsal segments from basal to apical = 1.1:1.2:1.0:1.1:2.3. Claws of all legs similar, one blunt and one hooked.

**Abdomen.** Terga II–VI each with pale median line on dorsal surface (Figure 25A). Terga II, IV–VII each with big dark brown spots and terga VII–IX each with pale spots on ventral surface (Figure 25C). All terga without posterolateral spines (Figure 25A,C). Cau-dal filaments dark brown, covered with spines (Figure 26D).

**Figure 27:**
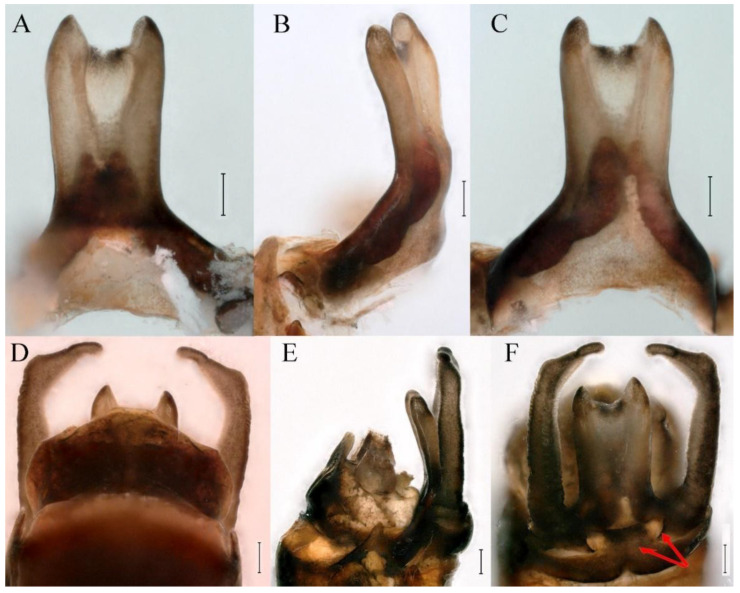
Male imago of *Cincticostella funki*: (**A**) penes (dorsal view); (**B**) penes (lateral view); (**C**) penes (ventral view); (**D**) genitalia (dorsal view); (**E**) genitalia (lateral view); (**F**) genitalia (ventral view), median convex lobe indicated by red arrow. Scale bar: 0.1 mm.

**Genitalia.** Dorsal margin of styliger plate with median convex lobe, the median lobe (including pale lateral extensions) between forceps bases overall boat-shaped in appearance (Figure 27F, indicated by red arrow). Second segment of forceps slightly constricted at point in apical fourth; segment 3 elongated, clavate (Figure 27A,C). Penis lobes compact (Figure 27A–C), expanded; penis lobes separated by rectangular cleft at point in apical ninth (Figure 27A–F), ratio of apicomedial length of rectangular cleft to maximum lobes length = 1: 1.6. Penis lobes with inverted triangular groove on apical half in both dorsal and ventral views (Figure 27A–C).

**Figure 28:**
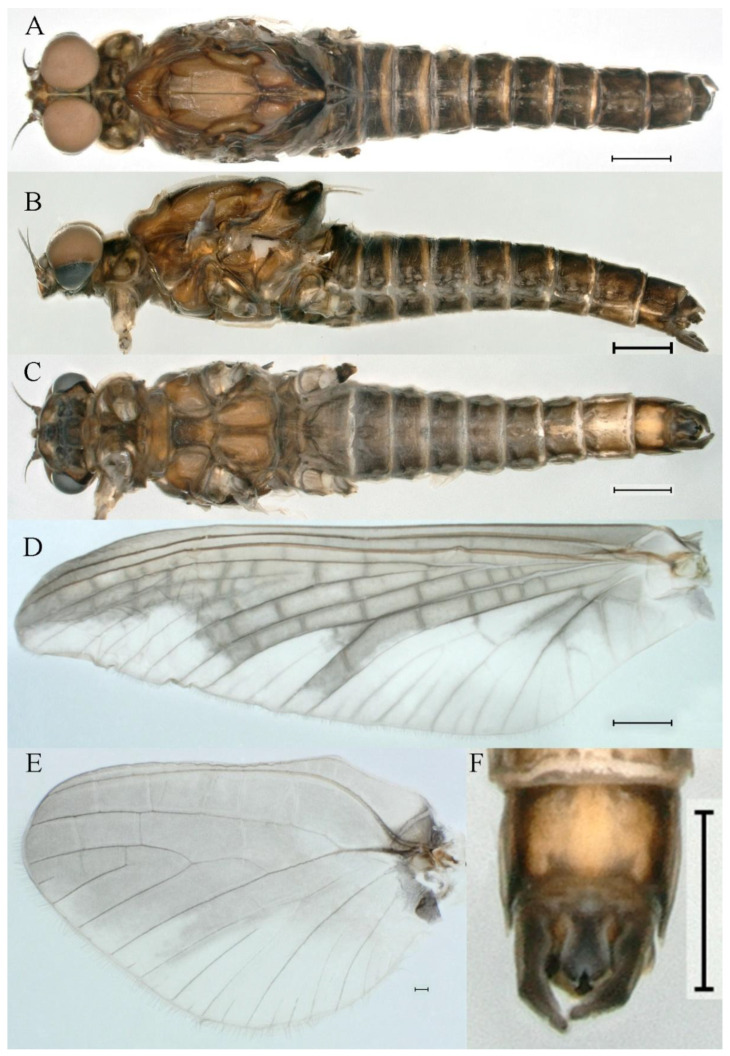
Male subimago of *Cincticostella funki*: (**A**) dorsal view; (**B**) lateral view; (**C**) ventral view; (**D**) forewing; (**E**) hindwing; (**F**) ventral view of genitalia. Scale bar: (**A**–**D**,**F**) = 1 mm; (**E**) = 0.1 mm.

**Figure 29:**
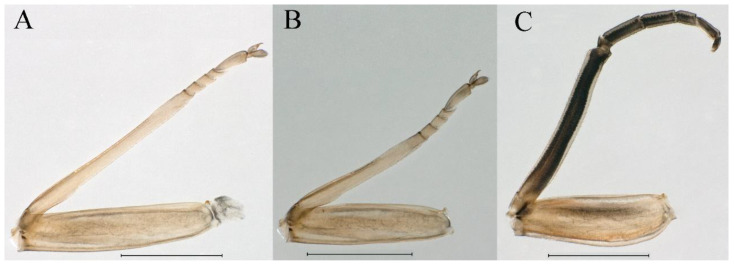
Male subimago of *Cincticostella funki*: (**A**) foreleg; (**B**) midleg; (**C**) hindleg. Scale bar: 1 mm.

***Male subimago*.** Body color brown to black (Figure 28A–C and Figure 33C). Forewing gray to black with crossveins infuscated (Figure 28D and Figure 33C). Front portion of hindwing taupe gray and rear portions nearly white (Figure 28E and Figure 33C). Mesonotum with three projections on posterior margin, middle projection longest (Figure 28A). Penes as in Figure 28F. Body length 10.80 mm (excluding tails), head width 2.00 mm, cerci length 10.42 mm, middle caudal filament 11.60 mm, forewing 11.48 mm, hindwing 3.10 mm. Margins of femur, tibia, and tarsus of foreleg, midleg, and hindleg densely covered with spines (Figure 29A–C). Length of femur:tibia:tarsus of foreleg = 1.0:1.2:1.1, tarsal segments from basal to apical = 1.0:1.9:1.7:1.3:1.3; femur:tibia:tarsus of midleg = 2.1:2.0:1.0, tarsal segments from basal to apical = 1.1:1.5:1.3:1.0:3.2; femur:tibia:tarsus of hindleg = 2.3:2.5:1.0, tarsal segments from basal to apical = 1.2:1.2:1.3:1.0:3.3.

**Figure 30:**
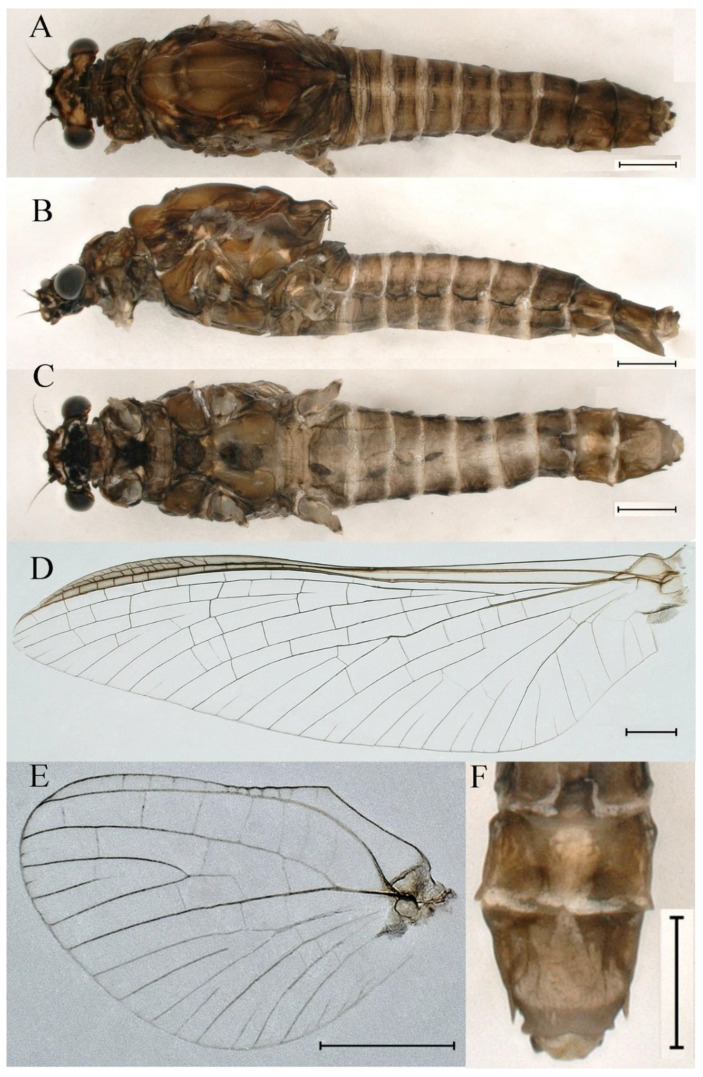
Female imago of *Cincticostella funki*: (**A**) dorsal view; (**B**) lateral view; (**C**) ventral view; (**D**) forewing; (**E**) hindwing; (**F**) posterior part of abdomen (ventral view). Scale bar: 1 mm.

**Figure 31:**
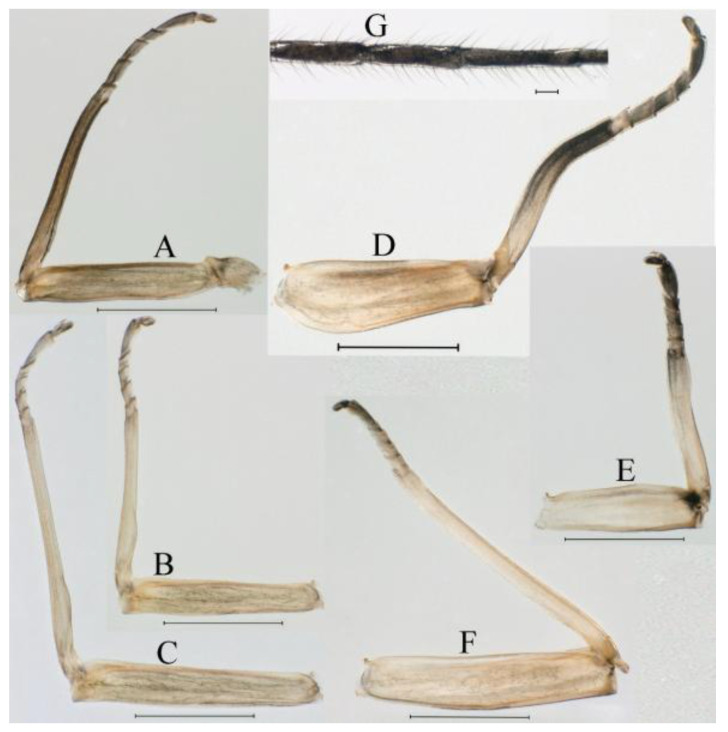
Female imago and subimago of *Cincticostella funki*: (**A**) foreleg of imago; (**B**) midleg of imago, (**C**) hindleg of imago; (**D**) foreleg of subimago; (**E**) midleg of subimago; (**F**) hindleg of subimago; (**G**) caudal filament. Scale bar: (**A**–**F**) = 1 mm; (**G**) = 0.1 mm.

***Female imago***. Color pattern similar to male (Figure 30A–C and Figure 33B); body length 11.44 mm (excluding tails), head width 1.94 mm, cerci length 12.94 mm, middle caudal filament 13.23 mm, forewing 12.26 mm (Figure 30D), hindwing 3.04 mm (Figure 30E). Lengths of femur (Figure 31A):tibia:tarsus of foreleg = 1.3:1.6:1.0, tarsal segments from basal to apical = 1.0:1.7:1.6:1.1:2.4; femur: tibia: tarsus of midleg (Figure 31B) = 2.0:1.8:1.0, tarsal segments from basal to apical = 1.3:1.9:1.8:1.0:3.8; femur:tibia:tarsus of hindleg (Figure 31C) = 2.4:2.7:1.0, tarsal segments from basal to apical = 1.1:1.3:1.4:1.0:3.3. Outer margins of femur and tibia of foreleg covered with spines. Posterior margin of subgenital plate produced to one-fifth length of sternum VIII. Posterior margin of subanal plate notched medially (Figure 30F). Color pattern of caudal filaments similar to male.

**Figure 32:**
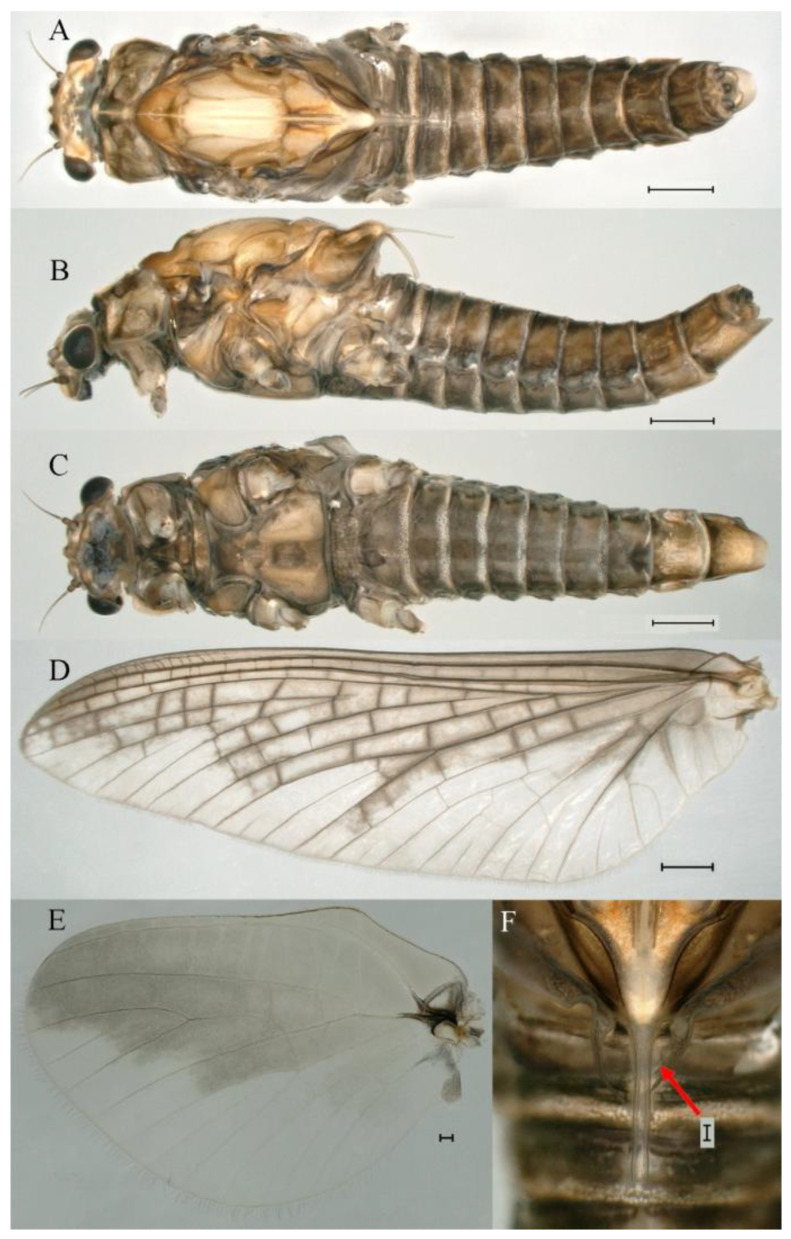
Female subimago of *Cincticostella funki*: (**A**) dorsal view; (**B**) lateral view; (**C**) ventral view; (**D**) forewing; (**E**) hindwing; (**F**) lateral scutellar projections, middle one indicated by red arrow. Scale bar: (**A**–**D**) = 1 mm; (**E**,**F**) = 0.1 mm.

**Figure 33:**
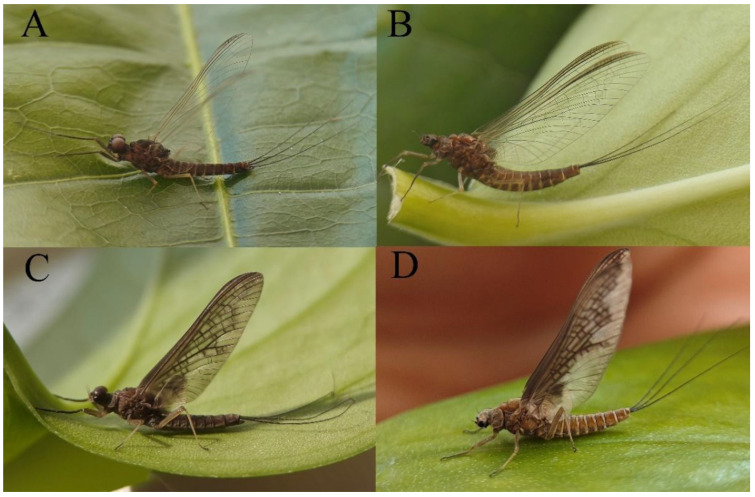
Winged stages of *Cincticostella funki* (living): (**A**) male imago; (**B**) female imago; (**C**) male subimago; (**D**) female subimago.

***Female subimago***. Similar to male subimago except for usual sexual differences (Figure 31D–F, Figure 32A–F and Figure 33D). Head width 2.24–2.45 mm, body length 11.93–12.59 mm (excluding tails), forewing length 14.45–14.80 mm, hindwing length 3.55–3.64 mm, cerci length 11.09–12.13 mm, middle caudal filament 10.12–11.80 mm. Length of femur:tibia:tarsus of foreleg (Figure 31D) = 1.5:1.5:1.0, tarsal segments from basal to apical = 1.1:1.6:1.4:1.0:2.6; femur:tibia:tarsus of midleg (Figure 31E) = 2.0:1.9:1.0, tarsal segments from basal to apical = 1.1:1.2:1.0:1.3:2.3; femur:tibia:tarsus of hindleg (Figure 31F) = 2.8:3.1:1.0, tarsal segments from basal to apical = 1.7:1.5:1.3:1.0:3.1. Caudal filaments relative densely covered with spines (Figure 31G).

**Figure 34:**
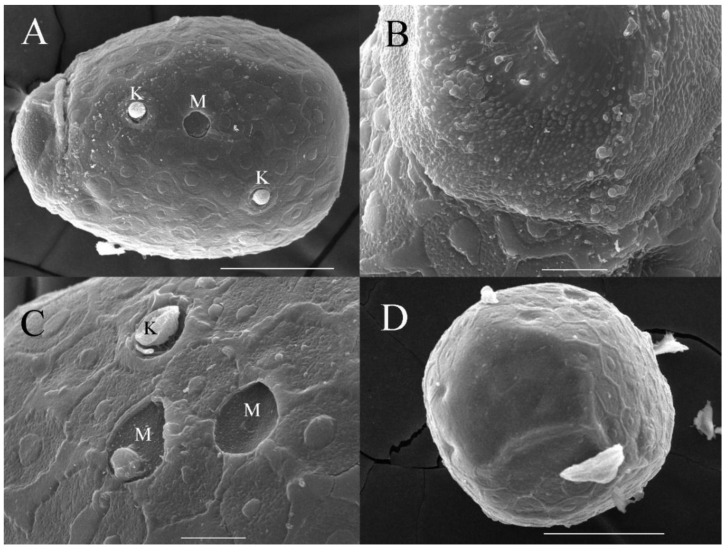
Egg of *Cincticostella funki*: (**A**) lateral view with micropyle (M) and knob of attachment structure (K); (**B**) polar cap; (**C**) micropyle (M) and attachment structure (K) enlarged; (**D**) bottom view. Scale bar: (**A**,**D**) = 0.05 mm; (**B**,**C**) = 0.01 mm.

***Eggs***. Dissected from female imago. Length 132–149 μm, width 96–107 μm. Ovoid with polar cap composed of dense filaments, each filament with intumescent terminal (Figure 34A,B). Chorion with irregular hexagonal strands; mesh with one tubercle medially; micropyle and knobs of attachment structure distributed near equator (Figure 34A,C).

**Remarks.** We examined specimens of different instars of *C. funki*, and the body coloration may vary between earlier and later instars. The early and middle instars of *C. funki* have relatively light body coloration with yellowish white markings on the thorax (such as Martynov et al.: figure 9A,C) [3] and legs, but the whole body of the last nymphal instar (living) is dark brown, and the white median line along the body is not obvious.

### 3.3. Cincticostella xiazhi Zi, Li & Jacobus, **sp. nov.**

Zoobank: urn:lsid:zoobank.org:act:FC861E87-29DD-4767-A911-5BE1BA7976CA

Figure 35, Figure 36, Figure 37, Figure 38, Figure 39, Figure 40, Figure 41, Figure 42, Figure 43, Figure 44, Figure 45, Figure 46, Figure 47, Figure 48, Figure 49, Figure 50, Figure 51, Figure 52 and Figure 53

**Type material. Holotype**: Male imago, with final nymphal instar exuviae (in ethanol), China, Yunnan Province, Dali City, Mt. Cangshan, Stream Qingbi, 25°39′08.6″ N 100°9′27.3″ E, 2200 m a.s.l., 21.VI.2024, coll. Xian-Fu Li and Yi-Fei Feng. **Paratypes**: 30 nymphs with same location as holotype, 28.V.2024, coll. Xian-Fu Li and Yi-Fei Feng; 1 female imago, 19.VI.2022, coll. Xian-Fu Li; 2 female imagoes, 23.VI.2022, coll. Xian-Fu Li; 1 female imago and 2 female subimagoes, 28.V.2024, coll. Xian-Fu Li; 2 male imagoes, 2 male subimagoes, and 2 female subimagoes, 21.VI.2024, coll. Xian-Fu Li; with same location as holotype.

**Etymology.** The name, xiazhi (neutral), comes from Xia Zhi, the tenth solar term of the 24 solar terms. The 24 solar terms are a traditional Chinese calendar system that divides the year into 24 periods, each lasting about two weeks. The emergence of *C. xiazhi* **sp. nov**. happened on the summer solstice. The English common name of this species is the summer solstice spiny crawler mayfly.

**Figure 35:**
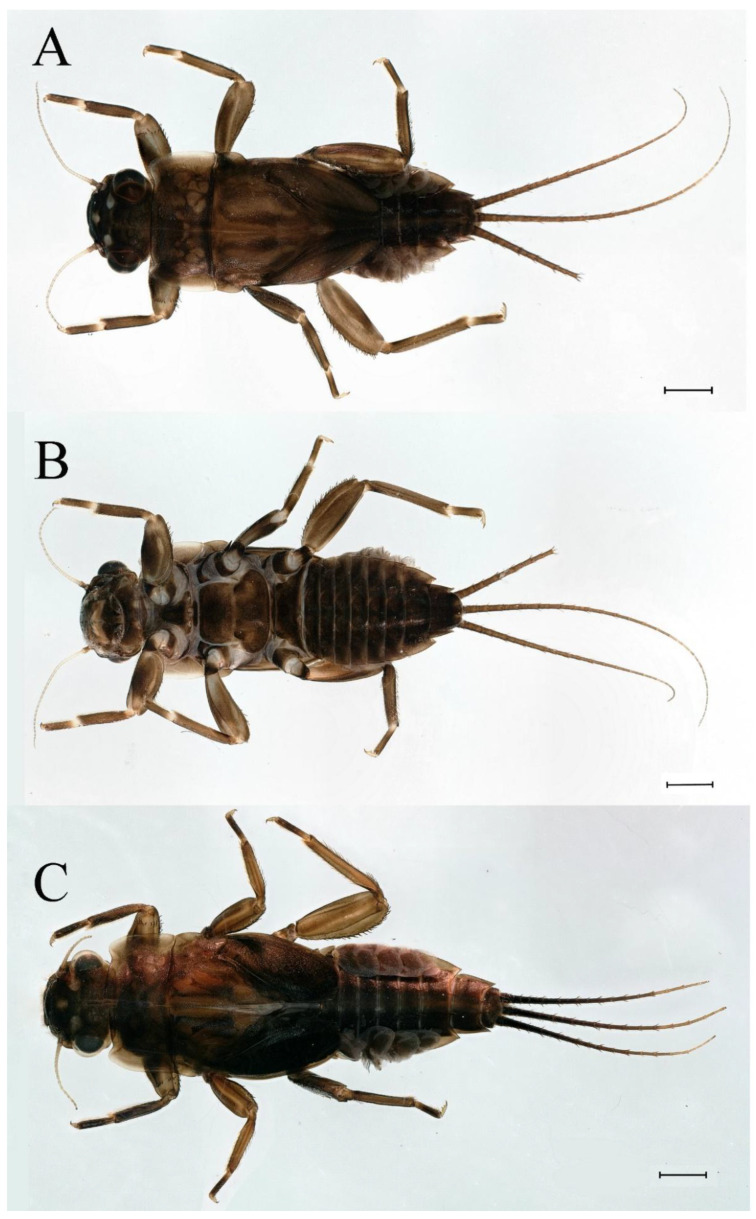
Final nymphal instar of *Cincticostella xiazhi* **sp. nov.**: (**A**) dorsal habitus of male; (**B**) ventral habitus of male; (**C**) dorsal habitus of female. Scale bar: 1 mm.

**Diagnoses.** The nymph of *C. xiazhi* **sp. nov.** is similar to *C. nigra* [10], *C. funki* [3], and *C. ebura* [4], because these nymphs have a white median line along their bodies (Figure 35A,C and Figure 53A,B) that can be used to separate them from other *Cincticostella* species. *Cincticostella xiazhi* **sp. nov.** can be distinguished from *C. nigra*, *C. funki*, and *C. ebura* by its claws. Claws of *C. ebura* and *C. funki* have 2 denticles [3,4], and claws of *C. nigra* have 6–8 denticles (Uéno: figure 9H,I) [9] or 5–8 denticles [10], but claws of *C. xiazhi* **sp. nov.** have 3 denticles.

**Descriptions. *Final nymphal instar***. Head width, male 2.09–2.26 mm, female 2.10–2.34 mm; body length (excluding tails), male 8.68–9.41 mm, female 8.77–10.18 mm; cerci length, male 6.05–6.21 mm, female 5.82–6.85 mm; middle caudal filament, male 6.19–7.17 mm, female 4.93–7.76 mm. Body black with dorsal median pale line from head to tergum X (Figure 35A,C).

**Figure 36:**
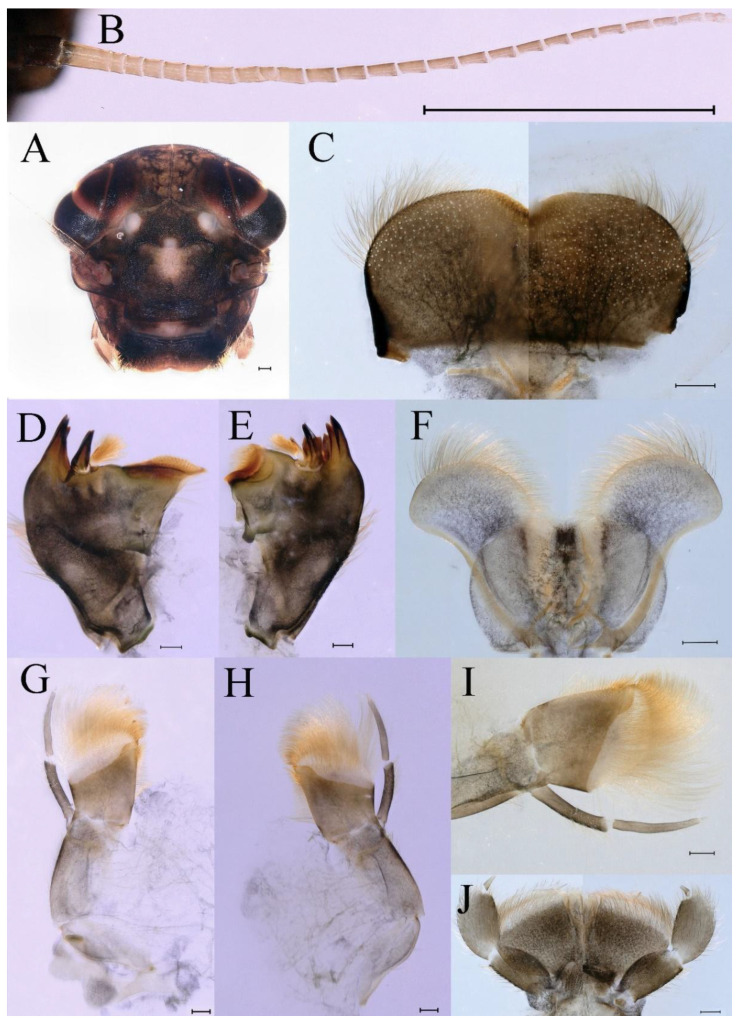
Nymphs of *Cincticostella xiazhi* **sp. nov.**: (**A**) head; (**B**) antenna; (**C**) labrum; (**D**) right mandible (ventral view); (**E**) left mandible (ventral view); (**F**) hypopharynx; (**G**) right maxilla (ventral view); (**H**) left maxilla (ventral view); (**I**) apex of left maxilla (ventral view); (**J**) labium. Scale bar: (**B**) = 1 mm; (**A**,**C**–**J**) = 0.1 mm.

**Head.** Black without tubercles, prominent bright ocelli (Figure 36A). Genae rounded, moderately developed (Figure 36A). Antennae without setae on articulations, antennae 1.5 times longer than head length (Figure 36B). Labrum densely covered with long, fine setae, apicolateral angles rounded, apicomedially with medium emargination; ratio of ratio of maximum width of emargination to maximum width of labrum = 1: 3.7 (Figure 36C). Mandibles stout with numerous, hair-like setae on proximal two-thirds of dorsal and lateral surfaces (Figure 36D,E). Right mandible: outer incisor composed of three pointed teeth; inner incisor composed of two apically pointed teeth; prostheca consisting of numerous hair-like setae (Figure 36D). Left mandible: outer incisor composed of four acute teeth; inner incisor composed of three apically pointed teeth; prostheca divided into two groups of numerous spines (Figure 36E). Hypopharynx: lingua recumbent, oval, with shallow anteromedian concavity, pale spot and short setae densely situated on anterolateral margins; superlinguae with curved outer, anterior margin and surface densely covered with long setae (Figure 36F). Maxillary palp long (1.04 mm), covered with tiny setae and three-segmented, length ratio from basal to apical segments = 11:12:1, apex of segment II with long, hair-like setae, segment III clavate and with tiny, short setae apically (Figure 36I); apex of maxilla widened, surface with numerous long, hair-like setae; maxillary maxillary canines reduced to small denticulated blade and less than half as long as crown; inner margin of galea-lacinia with rows of simple setae (Figure 36G–I). Labium with glossae elliptical and almost 1.4 times longer than broad and covered with numerous short, fine setae; paraglossae broad, semicircular, with surfaces covered with numerous simple setae (Figure 36J), pale spot situated on apical margin. Labial palp three-segmented; segments I and II stout and equal in length, outer margins covered with hair-like setae, segment III spine-like in shape, 2.4 times longer than broad at base (Figure 36J).

**Figure 37:**
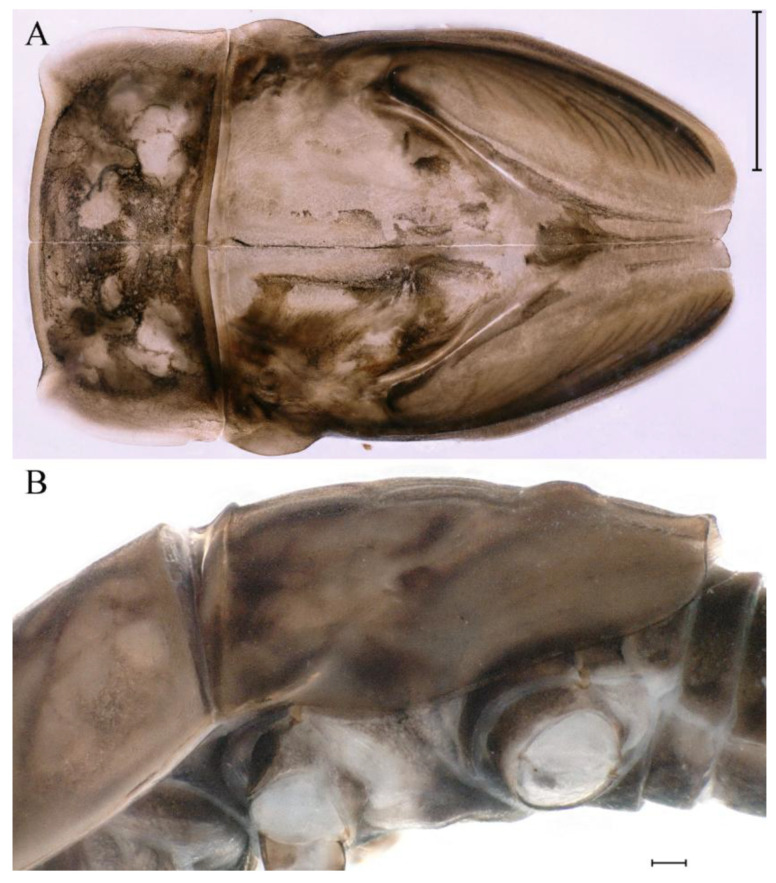
Nymphs of *Cincticostella xiazhi* **sp. nov.**: (**A**) thorax of last nymphal instar (dorsal view); (**B**) thorax of middle nymphal instar (lateral view). Scale bar: A = 1 mm; B = 0.1 mm.

**Figure 38:**
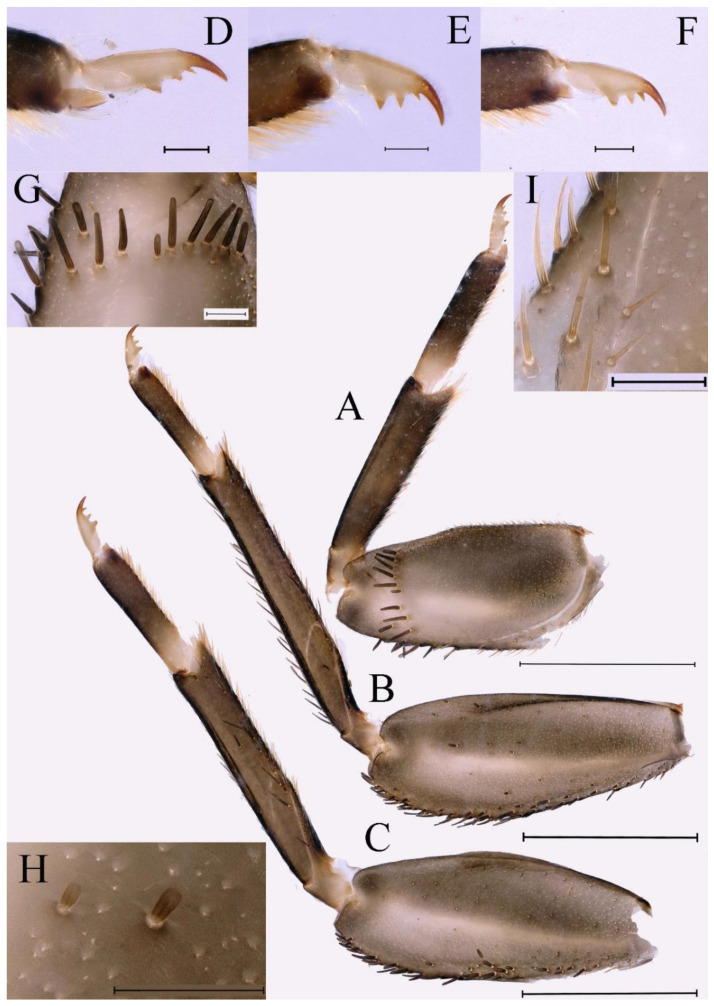
Nymphs of *Cincticostella xiazhi* **sp. nov.**: (**A**) foreleg; (**B**) midleg; (**C**) hindleg; (**D**) claw of foreleg; (**E**) claw of midleg; (**F**) claw of hindleg; (**G**) setae on femur; (**H**) setae on midleg femur; (**I**) setae on foreleg femur. Scale bar: (**A**–**C**) = 1 mm; (**D**–**I**) = 0.1 mm.

**Thorax.** Black with thin, white median line. Pronotum with moderately convex, rounded, and broad anterolateral angles. Mesonotum with rounded anterolateral projections, outer margins not notched (Figure 35A,C and Figure 37A), and with single prominent posteromedian tubercle (Figure 37B). Prothoracic sternum trapezoidal; mesothoracic basisternum rectangular; and mesothoracic furcastemum broader than basisternum, oval transversely (Figure 35B). Paired posterior projections between forewing pads small, rounded with medium cleft between them; apical parts of outer margins of projections not pressed against wing pads (Figure 37A). All femora slightly fattened (length/width ratio = fore femur 2.1; middle femur 2.5; hind femur 2.5) (Figure 38A–C), covered with scattered hair-like setae and scale sockets, each one with longitudinal ridge, especially prominent on middle and hind femora. Length ratio of leg segments (femora:tibia:tarsi): foreleg 1.7:1.5:1.0; middle leg 2.3:2.3:1.0; hindleg 2.2:2.2:1.0. All femora outer margins without apical projections, any distinct serration also absent. Fore femora moderately dilated, ventral margin smooth without setae, dorsal margin with long, pointed, stout setae (Figure 38I) along inner and outer margins and in basal part (Figure 38A), distal part of dorsal surface with transverse discontinuous row of about 10 spatulate setae (Figure 38A,G). Mid-femora and hind femora moderately expanded, dorsal margins with few short stout setae (Figure 38H) on surface and spatulate setae along outer margins (Figure 38B,C,I). Ventral surfaces of all tibiae and tarsi with solitary hair-like setae and long, thin, pointed, stout setae; stout setae situated most abundantly in apical parts of tibiae and tarsi and along their inner margins (Figure 38A–C). Tarsal claws of all legs hooked, with three denticles distantly spaced from each other; basal two denticles distinctly larger; distal denticle angled forward and easily broken; claws without subapical setae (Figure 38D–F).

**Figure 39:**
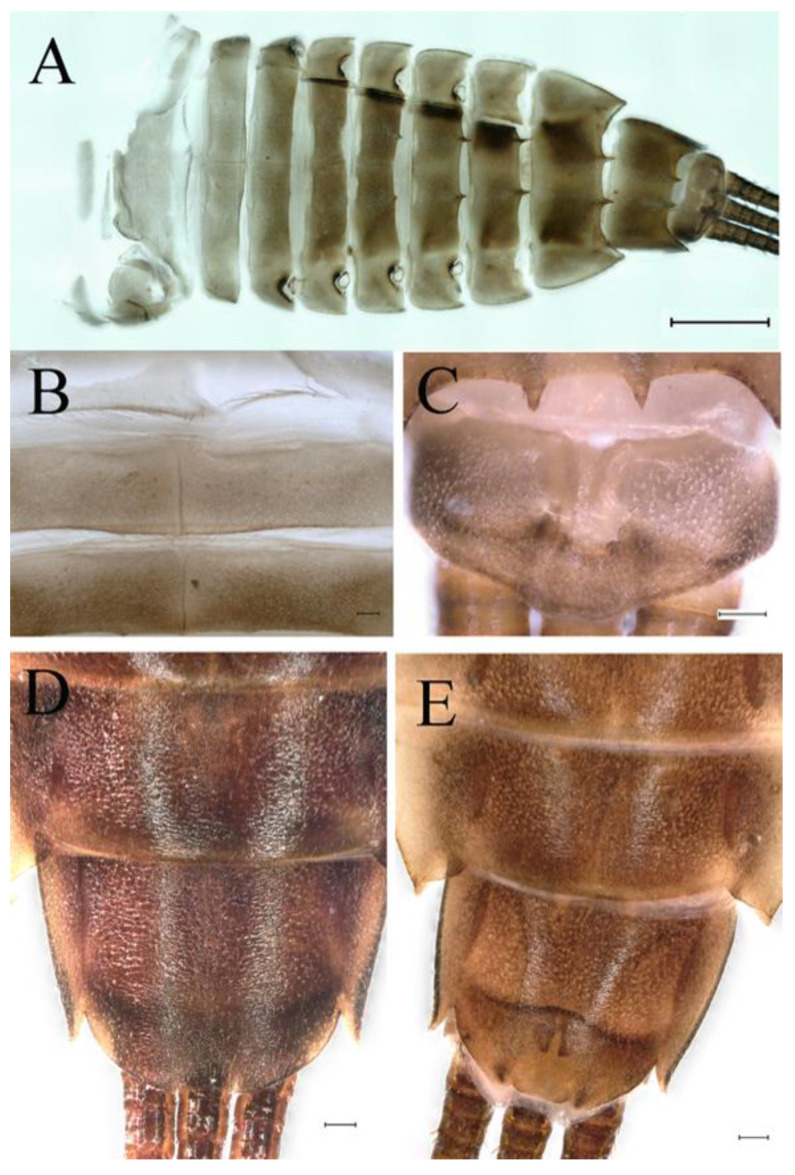
Nymphs of *Cincticostella xiazhi* **sp. nov.**: (**A**) abdomen (dorsal view); (**B**) setae of abdominal terga I and II; (**C**) abdominal terga IX and X enlarged; (**D**) posterior part of abdomen of female male (ventral view); (**E**) posterior part of abdomen of male (ventral view). Scale bar: (**A**) = 1 mm; (**B**–**E**) = 0.1 mm.

**Abdomen**. Posterior margins of terga I and II with thin and stout, hair-like setae (Figure 39B). Submedian surfaces of terga II–IX, posterior margins of terga VIII–X (excluding central area between submedian projections) and all paired submedian projections covered with small and middle-sized, oval, or sometimes, with slightly divergent margins, stout setae with rounded apices. All tergal surfaces covered with scale sockets. Pairs of pointed, not bifurcate, posteromedian projections present on abdominal terga II–IX; those on terga II–IV distinctly smaller than others (Figure 39A–C). Posterior margin of tergum X with small, stout setae with apices rounded, without projections (Figure 39C). Abdominal segments IV–IX with posterolateral projections; those on segments VIII and IX most developed and directed posterolaterally (Figure 39A). Lateral margins of terga IV–VIII covered with small, stout setae with apices rounded. Posterior margin of sternum IX of male slightly wavy (Figure 39E); posterior margin of sternum IX of female slightly concave (Figure 39D).

**Figure 40:**
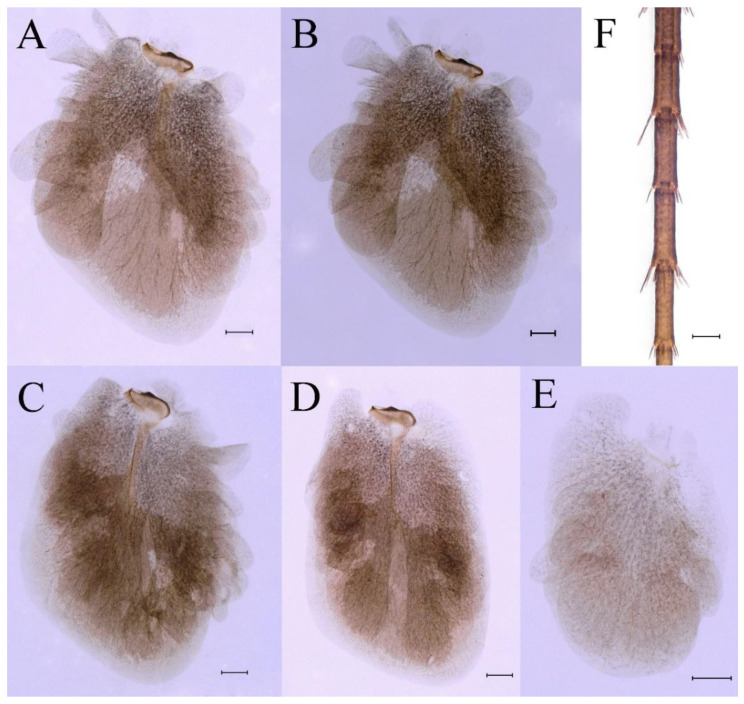
Nymphs of *Cincticostella xiazhi* **sp. nov.**: (**A**) gill III; (**B**) gill IV; (**C**) gill V; (**D**) gill VI; (**E**) gill VII; (**F**) caudal filaments. Scale bar: 0.1 mm.

Dorsal surface of abdominal gills covered with scale sockets, without scattered hair-like setae; shape of gills as in Figure 40A–E. Caudal filaments subequal in length, with mainly elongate, apically rounded, stout setae at articulations (Figure 40F).

**Figure 41:**
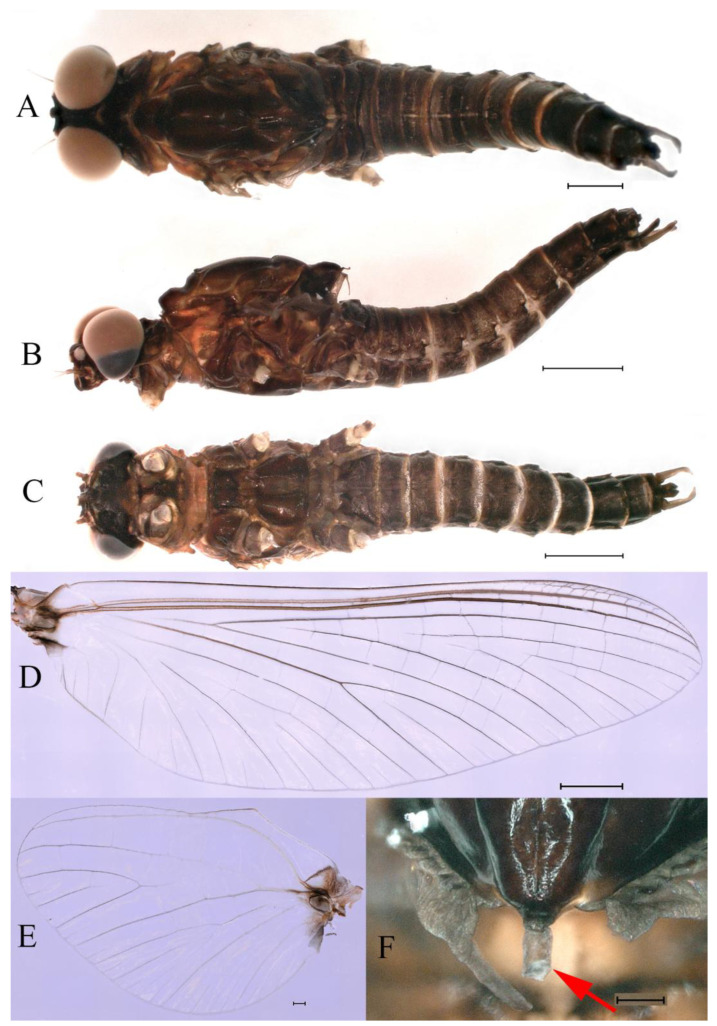
Male imago of *Cincticostella xiazhi* **sp. nov.**: (**A**) dorsal view; (**B**) lateral view; (**C**) ventral view; (**D**) forewing; (**E**) hindwing; (**F**) lateral scutellar projections, middle one indicated by red arrow. Scale bar: (**A**–**D**) = 1 mm; (**E**,**F**) = 0.1 mm.

***Male imago***. Body length 9.13–9.45 mm (excluding tails), head width 1.97–2.11 mm, forewing length 10.62–10.7 mm, hindwing length 3.17–3.22 mm, cerci length 10.78–11.93 mm, middle caudal filament 9.14–10.64 mm. Body color brown to dark brown (Figure 41A–C and Figure 51A).

**Head**. Compound eyes separated, upper portion brown and lower portion black (Figure 41B).

**Figure 42:**
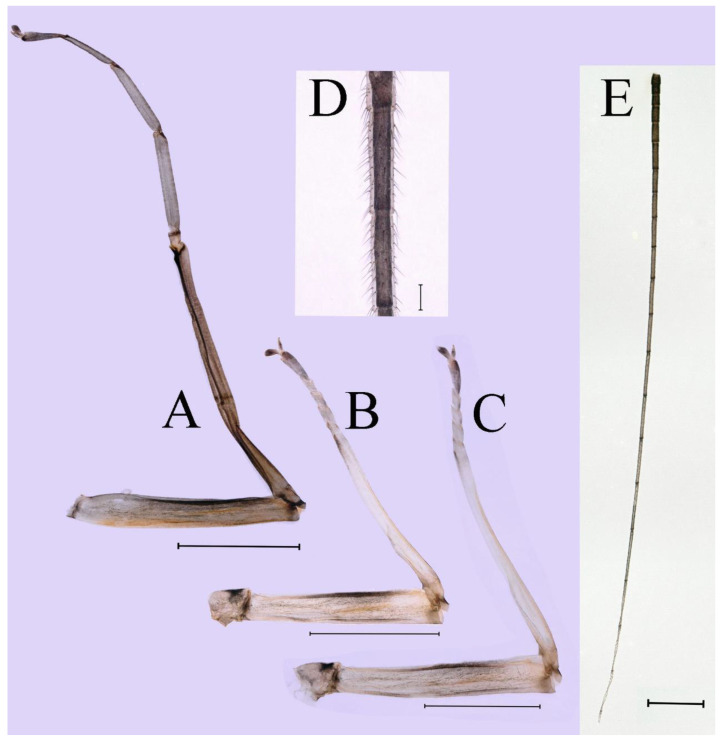
Male imago of *Cincticostella xiazhi* **sp. nov.**: (**A**) foreleg; (**B**) midleg; (**C**) hindleg; (**D**) caudal filament enlarged; (**E**) caudal filament. Scale bar: (**A**–**C**,**E**) = 1 mm; (**D**) = 0.1 mm.

**Thorax**. Pronotum with expanded posterolateral sac-like structure (Figure 41A). Prosternum dark brown, with slightly anteriorly converging longitudinal carinae, maximum width between carinae 4.7 times minimum width (Figure 41C). Basisternum of mesosternum dark brown, with parallel furcasternum (Figure 41C). Mesonotum with three projections on posterior margin, middle projection short (Figure 41F, indicated by red arrow). Forewings hyaline (Figure 41D), but C and Sc regions semihyaline; longitudinal veins dark brown and crossveins light brown to semihyaline; crossveins in stigma region between C and Sc separated into two parts by long vein; Rs leaves MA at very base, MA forked three-fifths of distance from base to margin; MP forked slightly more distally than fork of MA + Rs; CuP recurved strongly. Hindwing totally hyaline (Figure 41E), costal projection small, rounded, located at distance one-third from base to apex; MP forked between forks of R1 + MA and MA. Forelegs brown to dark brown (Figure 42A), mid- and hindlegs brown (Figure 42B,C). Femur:tibia:tarsus of foreleg = 1.0:1.7:1.5, tarsal segments from basal to apical = 1.0:4.3:4.4:2.8:1.5; femur:tibia:tarsus of midleg = 2.0:2.0:1.0, tarsal segments from basal to apical = 1.0:1.5:1.3:1.6:2.8; femur:tibia:tarsus of hindleg = 2.4:2.6:1.0, tarsal segments from basal to apical = 1.0:1.5:1.0:1.2:2.1. Claws of all legs similar, one blunt and one hooked.

**Abdomen.** Terga II–VI each with pale median lines (Figure 41A). Posterior margins of terga I–VIII lightly colored (Figure 41A–C). All terga without posterolateral spines, except tergum VIII (Figure 41C). Caudal filaments dark brown, covered with spines (Figure 42D,E).

**Figure 43:**
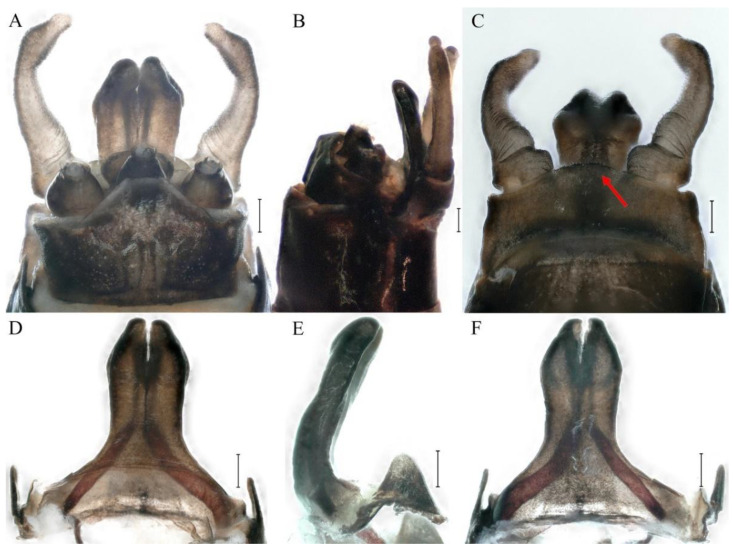
Male imago of *Cincticostella xiazhi* **sp. nov.**: (**A**) genitalia (dorsal view); (**B**) genitalia (lateral view); (**C**) genitalia (ventral view), median convex lobe indicated by red arrow; (**D**) penes (dorsal view); (**E**) penes (lateral view); (**F**) penes (ventral view). Scale bar: 0.1 mm.

**Genitalia.** Posterior margin of styliger plate round without obvious median convex lobe (Figure 43C). Second segment of forceps slightly constricted in apical third; segment 3 globular (Figure 43A–C). Penis lobes compact (Figure 43D–F), not expanded, pointed at apex and with slight subapical swelling; penis lobes with linear groove on apical half of dorsal face (Figure 43A,D), lobes separated by linear medial cleft in apical fourth or fifth, lateral margins slightly grooved subapically (Figure 43A,E).

**Figure 44:**
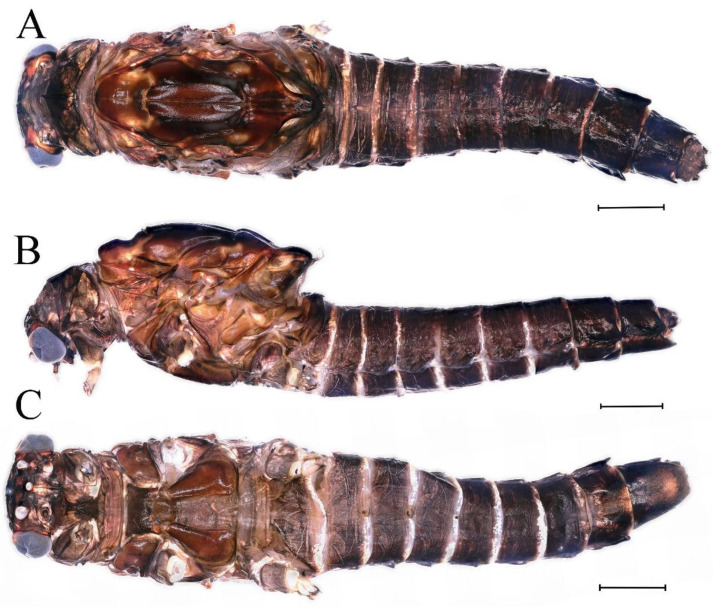
Female imago of *Cincticostella xiazhi* **sp. nov.**: (**A**) dorsal view; (**B**) lateral view; (**C**) ventral view. Scale bar: 1 mm.

**Figure 45:**
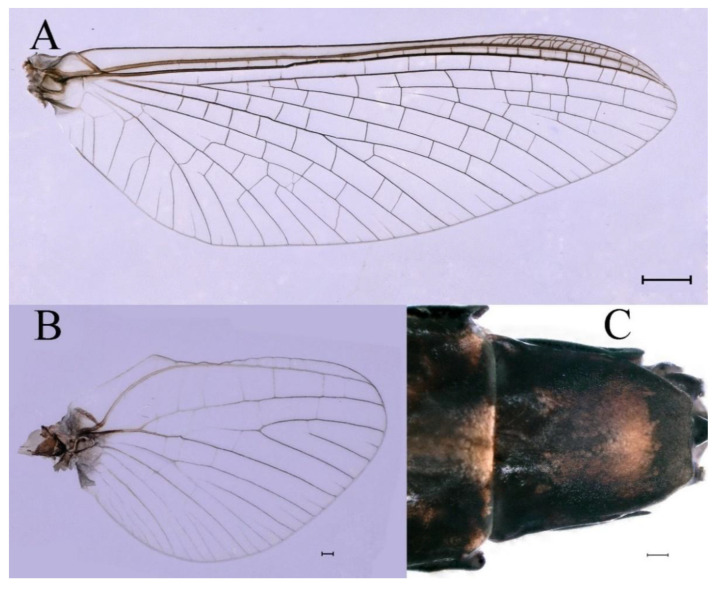
Female imago of *Cincticostella xiazhi* **sp. nov.**: (**A**) forewing; (**B**) hindwing; (**C**) posterior part of abdomen (ventral view). Scale bar: (**A**) = 1 mm; (**B**,**C**) = 0.1 mm.

**Figure 46:**
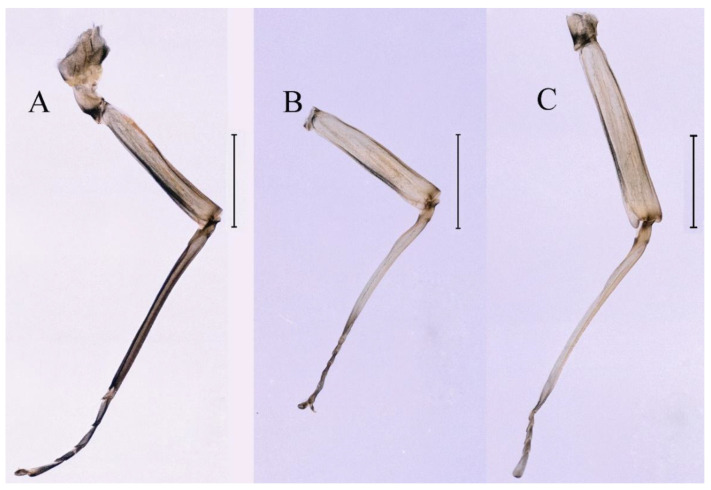
Female imago of *Cincticostella xiazhi* **sp. nov.**: (**A**) foreleg; (**B**) midleg; (**C**) hindleg. Scale bar: 1 mm.

***Female imago***. Color pattern similar to male (Figure 44A–C and Figure 51B); body length 10.58–11.06 mm (excluding tails), head width 1.89–1.91 mm, cerci length 10.95–11.46 mm, middle caudal filament 7.49 mm, forewing 13.14–15.01 mm (Figure 45A), hindwing 2.66–2.79 mm (Figure 45B). Lengths of femur (Figure 46A):tibia:tarsus of foreleg = 1.3:1.6:1.0, tarsal segments from basal to apical = 1.0:2.2:2.0:1.4:2.6; femur:tibia:tarsus of midleg (Figure 46B) = 1.9:2.0:1.0, tarsal segments from basal to apical = 1.0:1.1:1.1:1.4:2.4; femur:tibia:tarsus of hindleg (Figure 46C) = 2.4:2.7:1.0, tarsal segments from basal to apical = 1.0:1.9:1.9:2.2:2.7. Outer margins of femur and tibia of foreleg covered with spines. Posterior margin of subgenital plate produced to one-fifth length of sternum VIII. Posterior margin of subanal plate straight (Figure 45C). Color pattern of caudal filaments similar to male.

**Figure 47:**
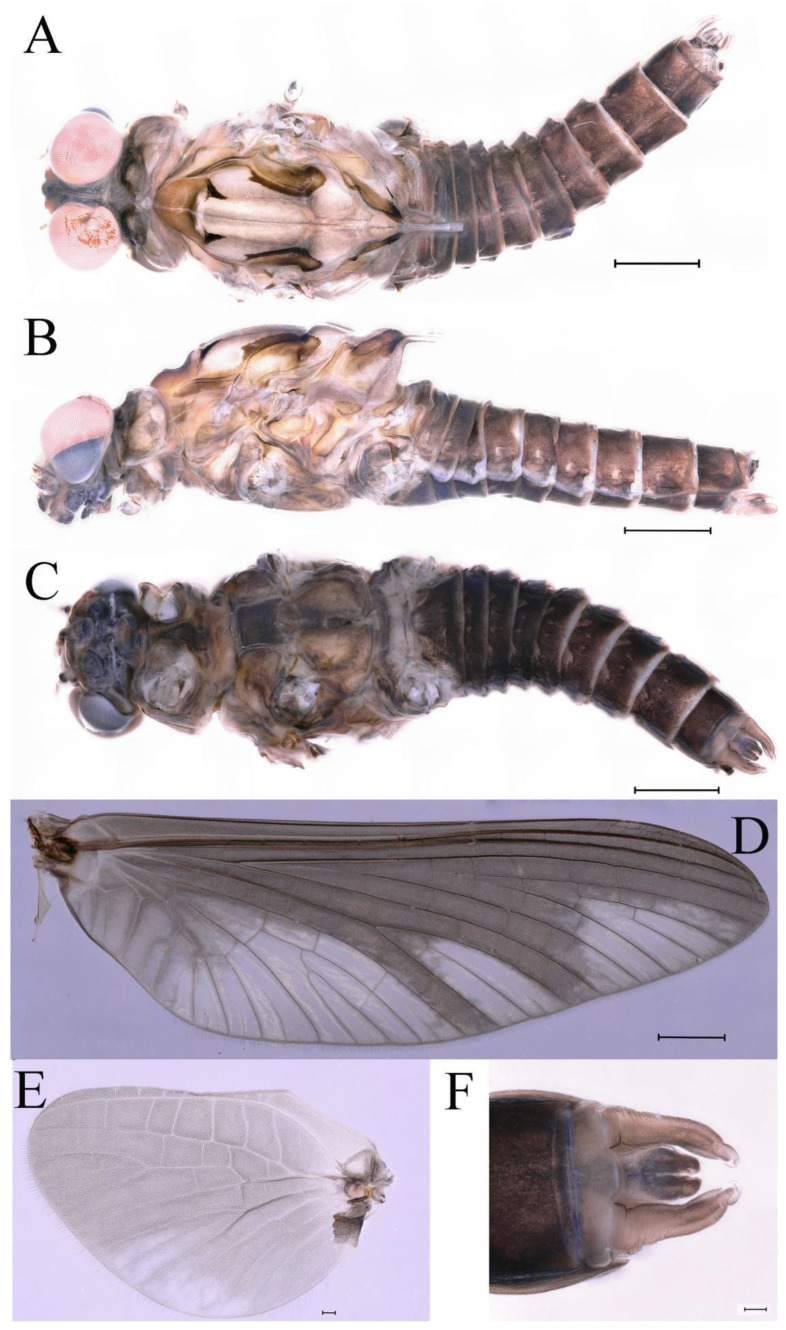
Male subimago of *Cincticostella xiazhi* **sp. nov.**: (**A**) dorsal view; (**B**) lateral view; (**C**) ventral view; (**D**) forewing; (**E**) hindwing; (**F**) ventral view of genitalia. Scale bar: (**A**–**D**) = 1 mm; (**E**,**F**) = 0.1 mm.

**Figure 48:**
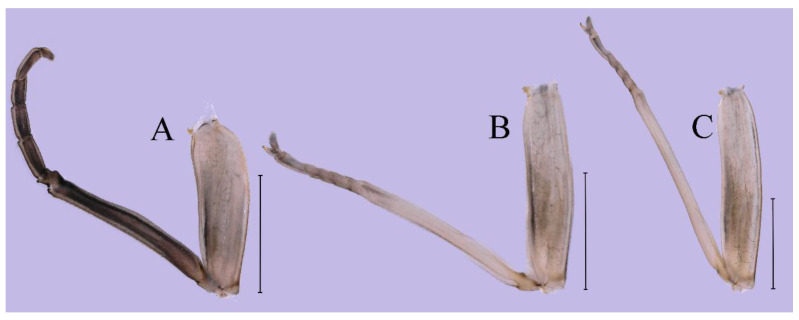
Male subimago of *Cincticostella xiazhi* **sp. nov.**: (**A**) foreleg; (**B**) midleg; (**C**) hindleg. Scale bar: 1 mm.

***Male subimago*.** Body color brown to black (Figure 47A–C and Figure 51C). Body length 7.59–10.07 mm (excluding tails), head width 2.00–2.28 mm, cerci length 8.61–11.00 mm, middle caudal filament 7.45–11.45 mm, forewing length 11.53–12.07 mm, hindwing length 2.07–2.81 mm. Forewing gray to black with crossveins infuscated (Figure 47D and Figure 51C), hindwing gray to black without crossvein infuscation (Figure 47E and Figure 51C). Mesonotum with three projections on posterior margin, middle projection longest (Figure 47A). Penes as in Figure 47F. Margins of femur, tibia and tarsus of foreleg, midleg, and hindleg densely covered with spines (Figure 48A–C). Length of femur:tibia:tarsus of foreleg = 1.2:1.3:1.0, tarsal segments from basal to apical = 1.0:2.4:2.0:1.3:3.1; femur: tibia: tarsus of midleg = 2.1:2.1:1.0, tarsal segments from basal to apical = 1.2:1.5:1.2:1.0:3.3; femur:tibia:tarsus of hindleg = 2.7:2.9:1.0, tarsal segments from basal to apical = 1.1:1.2:1.0:1.3:2.5.

**Figure 49:**
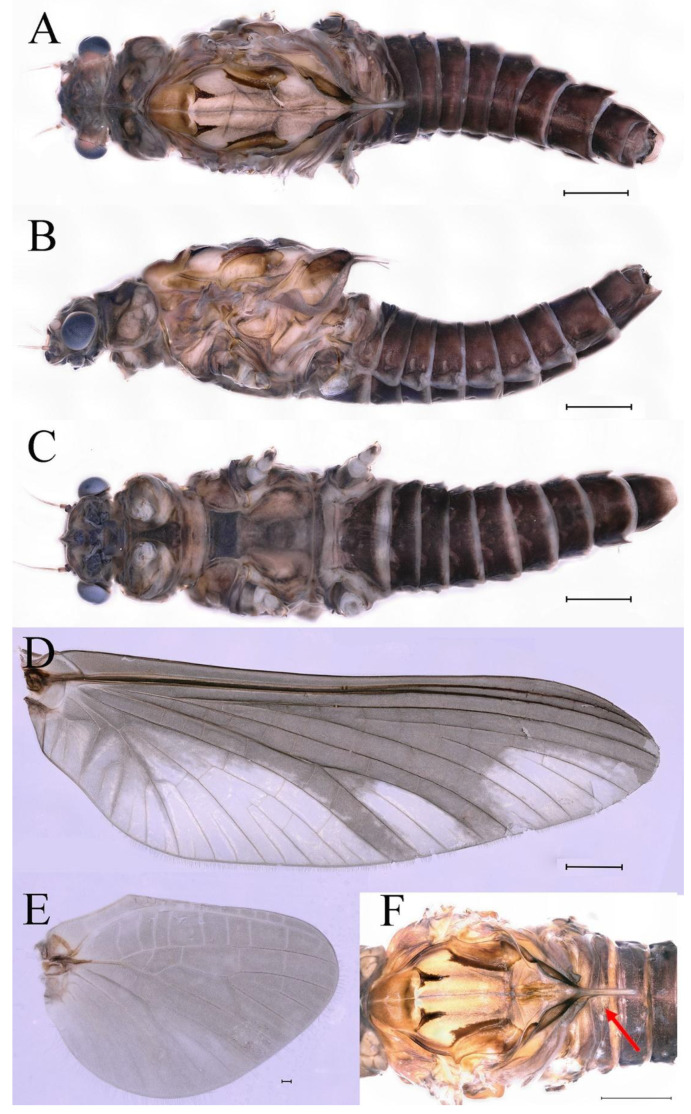
Female imago of *Cincticostella xiazhi* **sp. nov.**: (**A**) dorsal view of body; (**B**) lateral view of body; (**C**) ventral view of body; (**D**) forewing; (**E**) hindwing; (**F**) lateral scutellar projections, middle one indicated by red arrow. Scale bar: (**A**–**D**,**F**) = 1 mm; (**E**) = 0.1 mm.

**Figure 50:**
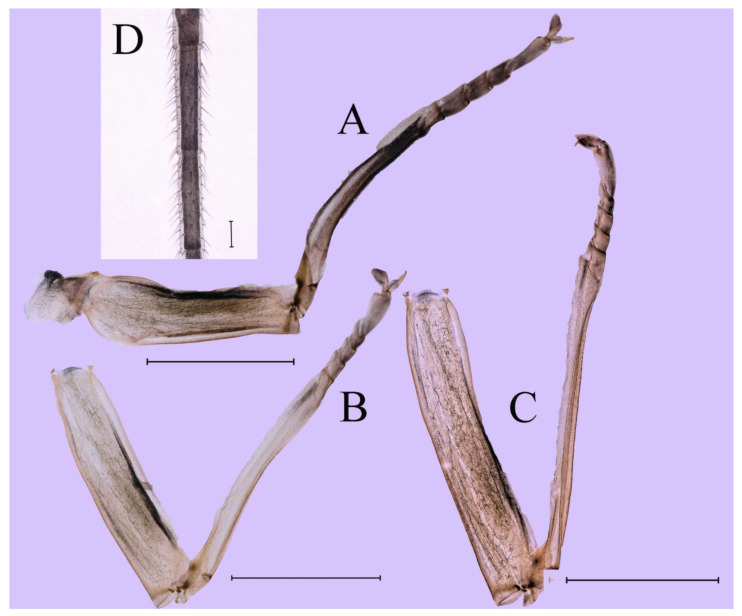
Female subimago of *Cincticostella xiazhi* **sp. nov.**: (**A**) foreleg; (**B**) midleg; (**C**) hindleg; (**D**) caudal filament. Scale bar: (**A**–**C**) = 1 mm; (**D**) = 0.1 mm.

**Figure 51:**
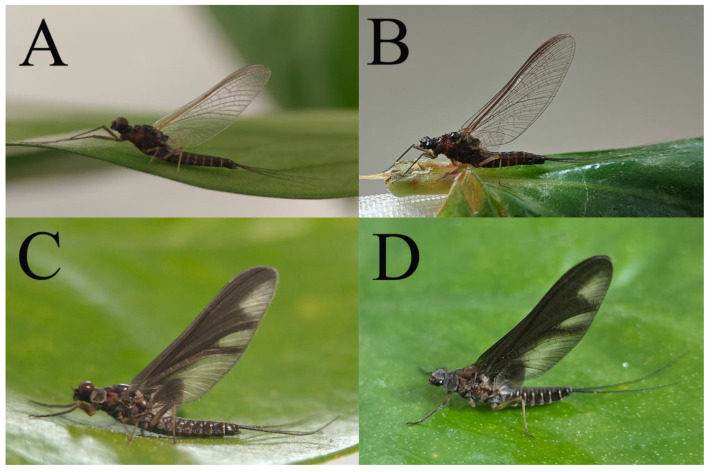
Winged stages of *Cincticostella xiazhi* **sp. nov.** (living): (**A**) male imago; (**B**) female imago; (**C**) male subimago; (**D**) female subimago.

***Female subimago***. Similar to male subimago except for usual sexual differences (Figure 49A–F and Figure 51D). Head width 1.93–2.03 mm, body length 9.52–9.69 mm (excluding tails), forewing length 11.32–13.01 mm, hindwing length 3.01–3.23 mm, cerci length 9.15–9.38 mm, middle caudal filament 8.15–8.88 mm. Length of femur:tibia:tarsus of foreleg (Figure 50A) = 1.0:1.3:1.0, tarsal segments from basal to apical = 1.0:2.8:2.4:1.6:2.1; femur:tibia:tarsus of midleg (Figure 50B) = 2.0:2.0:1.0, tarsal segments from basal to apical = 1.0:1.3:1.3:1.2:2.6; femur:tibia:tarsus of hindleg (Figure 50C) = 2.5:2.6:1.0, tarsal segments from basal to apical = 1.0:1.4:1.3:1.5:3.0. Caudal filaments relatively densely covered with spines (Figure 50D).

**Figure 52:**
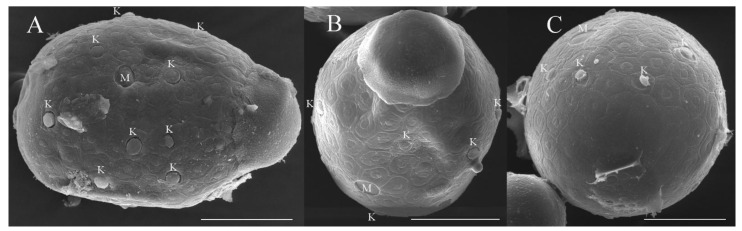
Egg of *Cincticostella xiazhi* **sp. nov.**: (**A**) lateral view with micropyle (M) and knob of attachment structure (K); (**B**) polar cap; (**C**) bottom view. Scale bar: 0.05 mm.

***Eggs***. Dissected from female imago. Length 145–163 μm, width 101–114 μm. Ovoid with polar cap composed of dense filaments, each filament with intumescent terminal (Figure 52A,B). Chorion with irregular polygonal strands (Figure 52A–C); mesh with one tubercle medially; micropyles distributed near equator; chorion with knobs of attachment structure except in subpolar areas (Figure 52A–C).

**Figure 53:**
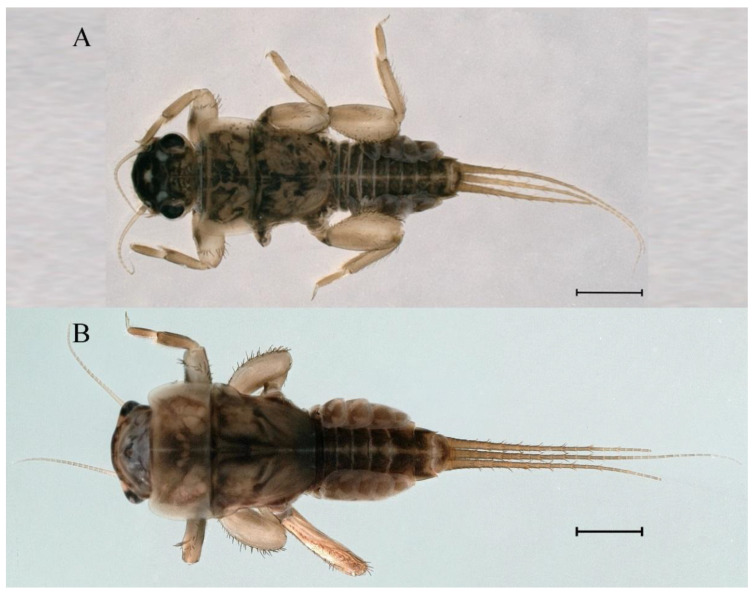
Nymphs of *Cincticostella xiazhi* **sp. nov.**: (**A**) early instar (dorsal view); (**B**) middle instar (dorsal view). Scale bars: 0.1 mm.

**Remarks.** Different nymphal instars (Figure 18A,C, Figure 39A and Figure 53A,B) and the subimago (Figure 47A and Figure 49A) of *C. xiazhi* **sp. nov.** always have the white median line along the body, similar to *C. nigra*, *C. funki*, and *C. ebura*. However, *C. ebura* has very unique characteristics of the abdomen [4]. The remaining three species, *C. funki*, *C. nigra*, and *C. xiazhi* **sp. nov.**, have different geographic areas, ecological factors, and biological behavior. *Cincticostella nigra* is only reported from the east Palaearctic [9]; *C. funki* has an Indomalayan distribution including northern India, northern Thailand [3], and southwestern Yunnan Province, China, while *C. xiazhi* **sp. nov.** is reported from northwestern Yunnan Province, China, which is the transition zone between the eastern Palearctic and Indomalayan regions [20]. Late instar nymphs of *C. funki* were collected in December from a stream with water temperature of 12 °C at 1285 m a.s.l. [3] and in March at 1526 m a.s.l. (herein), but late instar nymphs of *C. xiazhi* **sp. nov.** were collected in June from a stream with water temperature of 9–15 °C at 2200–2500 m a.s.l. Final nymphal instars of *C. funki* [3] and *C. ebura* [4] were collected at times when only small nymphs of *C. nigra* [20] and *C. xiazhi* **sp. nov.** (pers. obs., Xian-Fu Li) might be collected, each in its respective location.

**Distribution.** Yunnan, China.

### 3.4. Cincticostella yushui Zi, Li & Jacobus, **sp. nov.**

Zoobank: urn:lsid:zoobank.org:act:8B32EA40-D757-4978-9108-F91C11225ECA

Figure 54, Figure 55, Figure 56, Figure 57, Figure 58, Figure 59, Figure 60, Figure 61, Figure 62, Figure 63, Figure 64, Figure 65, Figure 66, Figure 67, Figure 68 and Figure 69

**Type material. Holotype**: Male imago, with final nymphal instar exuviae, China, Yunnan Province, Dali City, Yunlong County, Tuanjie Township, west slope of Mt. Cangshan, a Shunbi River feeder stream, 25°45′45.5″ N 99°38′5.8″ E, 2284 m a.s.l., 22.II.2025, coll. Xian-Fu Li. **Paratypes**: 12 nymphs, 6 female imagoes, 5 male and 5 female subimagoes with same data and location as holotype, coll. Xian-Fu Li; 3 male and 3 female imagoes reared from nymphs, with same location as holotype, 20.II.2025, coll. Xian-Fu Li.

**Etymology.** The name, yushui (neutral), comes from Yu Shui, the second solar term of the 24 solar terms. The name of this term means rainwater. The emergence of *C. yushui* **sp. nov.** happened during this term. The English common name of this species is the rainwater spiny crawler mayfly.

**Diagnoses.** The nymph of *C. yushui* **sp. nov.** is similar to *C. elongatula* and *C. shinichii* Martynov & Palatov, 2021*,* because these nymphs lack a white median line along their bodies, have posteromedian projections on terga V–IX relatively well-developed, and terga I and X lack posteromedian projections [3,9]. *Cincticostella yushui* **sp. nov.** can be distinguished from *C. shinichii* and *C. elongatula* by claws, labrum, and geographic distribution. The tarsal claw of *C. shinichii* has one denticle [3]; *C. yushui* **sp. nov.** has four denticles (with both of these species occurring in the Himalayan region), and *C. elongatula* has five to eight denticles (occurring in the eastern Palearctic region) [9]. The anteromedian emargination of the labrum of *C. shinichii* is relatively deep and wide [3], while the labral emarginations of *C. elongatula* [9] and *C. yushui* **sp. nov.** are shallow and narrow.

**Figure 54:**
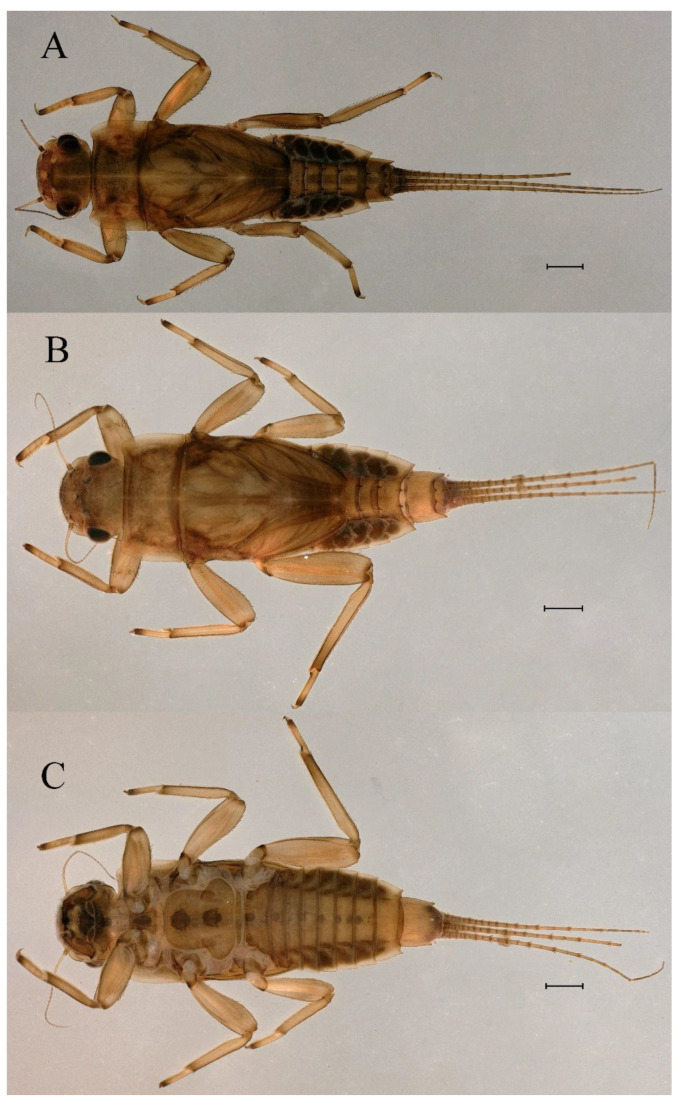
Last nymphal instar of *Cincticostella yushui* **sp. nov.**: (**A**) dorsal habitus of male; (**B**) dorsal habitus of female; (**C**) ventral habitus of female. Scale bars: 1 mm.

**Descriptions.** *Final nymphal instar*. Head width, male 2.18–2.31 mm, female 2.12–2.44 mm; body length (excluding tails), male 9.59–12.02 mm, female 10.33–11.29 mm; cerci length, male 5.72–7.07 mm, female 5.65–6.92 mm, middle caudal filament, male 6.17–6.46 mm, female 6.22–8.17 mm. Body brown (Figure 54A–C).

**Figure 55:**
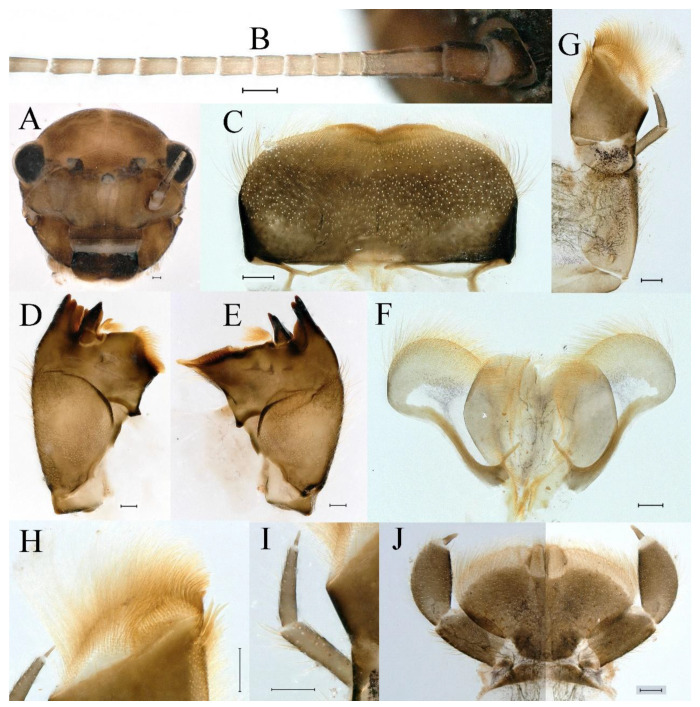
Nymphs of *Cincticostella yushui* **sp. nov.**: (**A**) head; (**B**) antenna; (**C**) labrum; (**D**) left mandible; (**E**) right mandible; (**F**) hypopharynx (ventral view); (**G**) right maxilla; (**H**) apex of right maxilla (ventral view); (**I**) maxillae palp; (**J**) labium (ventral view). Scale bars: 0.1 mm.

**Head.** Brown without tubercles, ocelli prominent and bright (Figure 55A). Genae rounded, moderately developed (Figure 55A). Antennae without setae on articulations, antennae 1.2 times longer than head length (Figure 55B). Labrum densely covered with long, fine setae, apicolateral angles rounded, apicomedially with shallow emargination; ratio of maximum width of emargination to maximum width of labrum = 1:5.6 (Figure 55C). Mandibles stout with numerous, hair-like setae on proximal two-thirds of dorsal and lateral surfaces (Figure 55D,E). Right mandible: outer incisor composed of three pointed teeth; inner incisor composed of two pointed teeth; prostheca consisting of numerous hair-like setae (Figure 55D). Left mandible: outer incisor composed of four acute teeth; inner incisor composed of three pointed teeth; prostheca divided into two groups of numerous spines (Figure 55E). Hypopharynx: lingua round, with medium anteromedian concavity, and short setae densely situated on anterolateral margins; superlinguae with curved outer, anterior margin and surface densely covered with long setae (Figure 55F). Maxillary palpi long (1.04 mm) and three-segmented, covered with tiny setae on surfaces of segments I, II, length ratio from basal to apical segments = 6.2:5.7:1 (Figure 55G–I), segment III clavate and without setae (Figure 55H); apex of maxilla round, surface with numerous long, hair-like setae; maxillary canine reduced to small denticulate blade and about half as long as crown, inner margin of galea-lacinia with rows of simple setae (Figure 55G–I). Labium with glossae elliptical and almost 2.2 times longer than broad and covered with numerous short, fine setae; paraglossae broad, semicircular, with surfaces covered with numerous simple setae (Figure 55J), pale spot situated on apical margins. Labial palp three-segmented; segments I and II stout and equal in length, outer margin covered with hair-like setae, segment III spine-like in shape, 2.3 times longer than broad at base Figure 55J).

**Figure 56:**
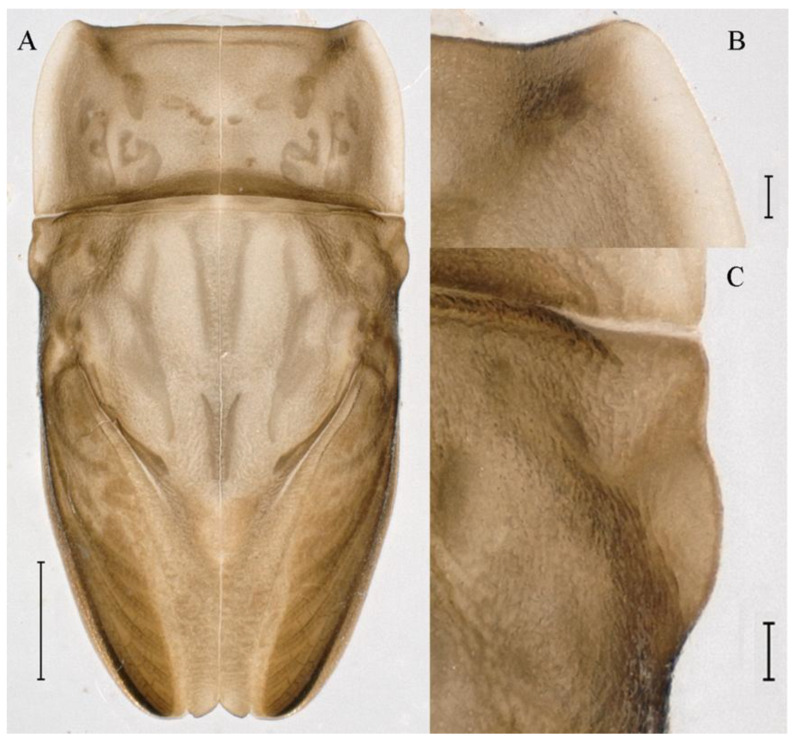
Nymphs of *Cincticostella yushui* **sp. nov.**: (**A**) thorax of last nymphal instar (dorsal view); (**B**) anterolateral projections of pronotum; (**C**) anterolateral projections of mesothorax. Scale bar: (**A**) = 1 mm; (**B**,**C**) = 0.1 mm.

**Figure 57:**
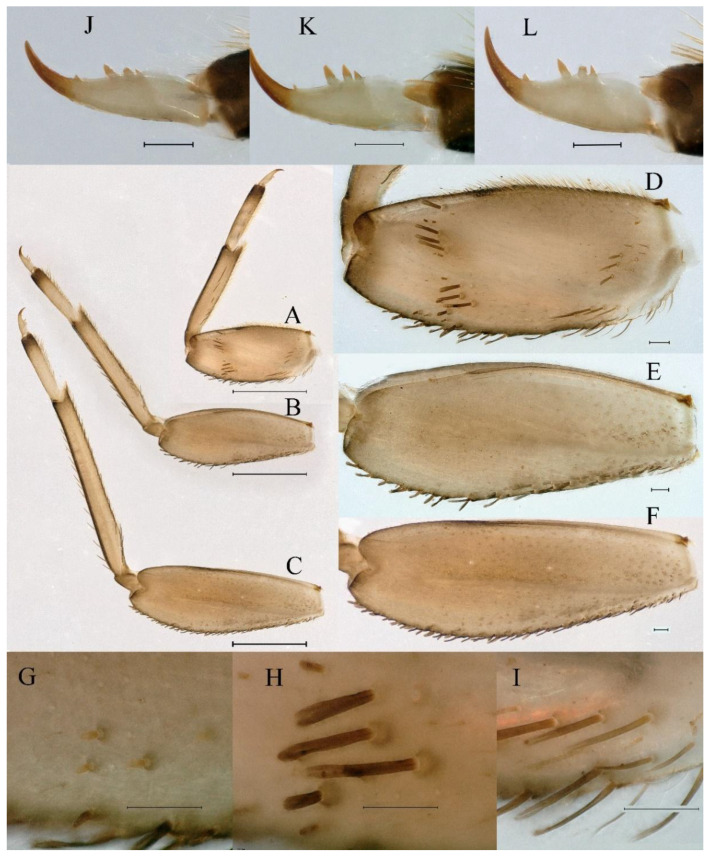
Nymphs of *Cincticostella yushui* **sp. nov.**: (**A**) foreleg; (**B**) midleg; (**C**) hindleg; (**D**) fore femur; (**E**) middle femur; (**F**) hind femur; (**G**) setae of dorsal surface of hind femur; (**H**) setae of dorsal surface of fore femur; (**I**) setae of outer margin of fore femur; (**J**) claw of foreleg; (**K**) claw of midleg; (**L**) claw of hindleg. Scale bar: (**A**–**C**) = 1 mm; (**D**–**L**) = 0.1 mm.

**Thorax.** Pronotum with moderately convex, rounded, and broad anterolateral angles. Anterolateral projections of mesothorax well-developed, not notched, broad, rounded, with outer margins not subparallel to lateral aspect of body (Figure 54A,B and Figure 56A,B). Prothoracic sternum trapezoidal; mesothoracic basisternum rectangular; and mesothoracic furcastemum broader than basisternum, oval transversely (Figure 54C). Paired posterior projections between forewing pads small, rounded with medium cleft between them; apical parts of outer margins of projections not pressed against wing pads (Figure 56A). All femora slightly fattened (length/width ratio = fore femur 2.2; middle femur 2.5; hind femur 2.8) (Figure 57A–F), covered with scattered hair-like setae and scale sockets, each one with longitudinal ridge, especially prominent on hind femur (Figure 57D–F). Length ratio of leg segments (femora:tibia:tarsi): foreleg 1.6:1.4:1.0; middle leg 2.4:2.3:1.0; hindleg 2.5:2.8:1.0. All femora outer margins without apical projections, any distinct serration also absent, with hair-like setae on dorsal surfaces. Fore femur moderately dilated, dorsal margin smooth with long, pointed, stout, hair-like setae along inner margin, and long, bifurcated, stout setae (Figure 57I) along outer margin and in basal part (Figure 57D), distal part of dorsal surface with transverse discontinuous row of about 10 long, bifurcated, stout setae (Figure 57A,D,H). Mid-femur moderately expanded, dorsal margin with few short, stout setae in basal part and long, bifurcated, stout setae along outer margins (Figure 57E). Hind femur moderately expanded, dorsal margin with few short, stout setae on surface and long, bifurcated, stout setae along outer margins (Figure 57F,G). Ventral surfaces of all tibiae and tarsi with solitary hair-like setae and long, thin, pointed, stout setae; stout setae situated most abundantly on apical parts of tibiae and tarsi and along their inner margins (Figure 57A–C). Tarsal claw of all legs hooked, with four denticles; three basal denticles distanced from one distal denticle, middle two denticles distinctly larger; basal and distal denticles small and easily broken; claw without subapical setae (Figure 57J–L).

**Figure 58:**
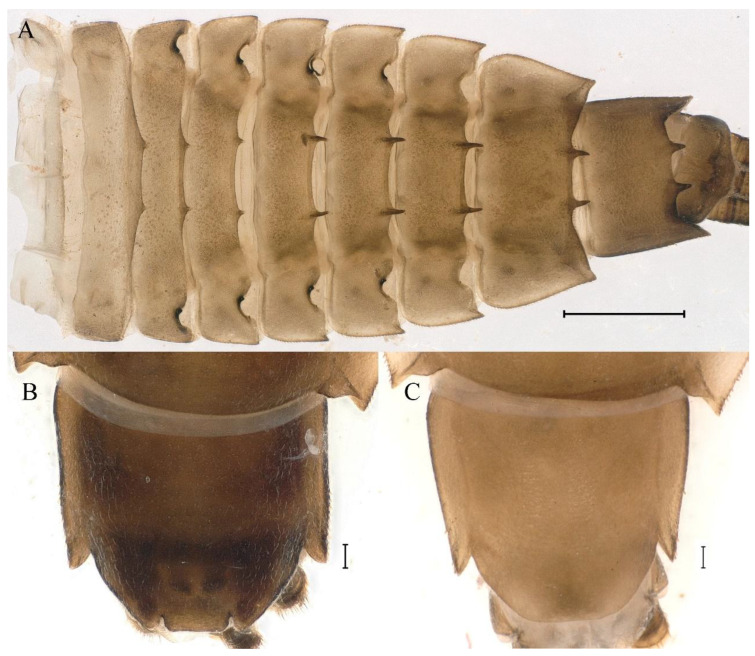
Nymphs of *Cincticostella yushui* **sp. nov.**: (**A**) abdomen (dorsal view); (**B**) posterior part of abdomen of male (ventral view); (**C**) posterior part of abdomen of female (ventral view). Scale bar: **A** = 1 mm; (**B**,**C**) = 0.1 mm.

**Abdomen**. Posterior margins of terga I and II with thin and stout, hair-like setae (Figure 58A). Posterior margins of terga VIII, IX (excluding central area between submedian projections) covered with middle-sized, stout setae with rounded apices. All tergal surfaces covered with scale sockets. Submedian areas of terga II–IX surfaces and posterior margins of terga II–VII (excluding central area between submedian projections) covered with small, stout setae with rounded apices. Pairs of pointed, not bifurcate, posteromedian projections present on abdominal terga II–IX; those on terga II–IV distinctly smaller than others (Figure 58A). Posterior margin of tergum X without stout setae and projections. Lateral margins of terga IV–VIII covered with small, stout setae with apices rounded. Abdominal segments V–IX with posterolateral projections; segment IX posterolateral projections most developed and directed distinctly posterolaterally. Posterior margin of sternum IX of male distinctly straight (Figure 58B); posterior margin of sternum IX of female almost straight (Figure 58C).

**Figure 59:**
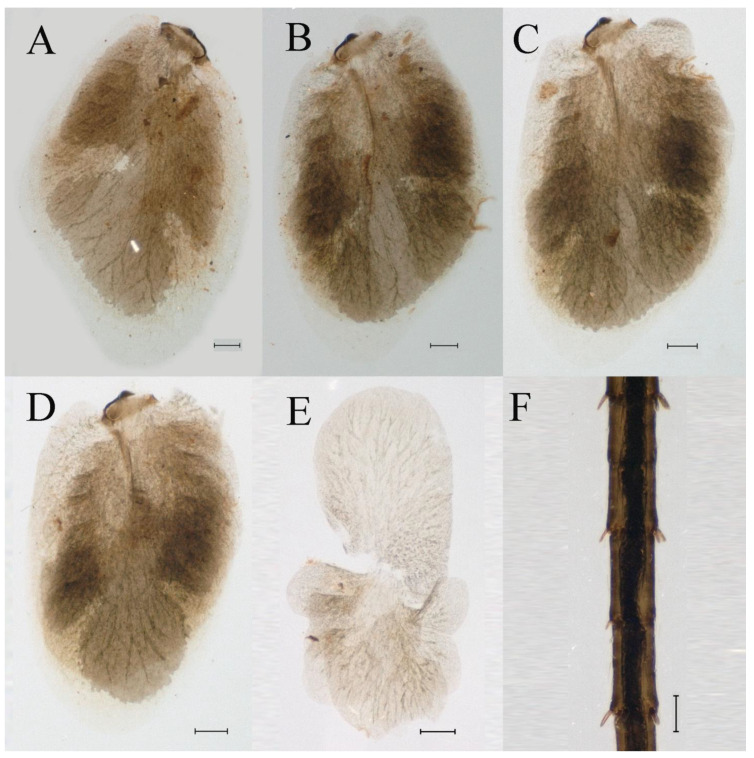
Nymphs of *Cincticostella yushui* **sp. nov.**: (**A**) gill III; (**B**) gill IV; (**C**) gill V; (**D**) gill VI; (**E**) gill VII; (**F**) caudal filament. Scale bar: 0.1 mm.

Dorsal surface of abdominal gills covered with scale sockets, without scattered hair-like setae; shapes of gills as in Figure 59A–F. Caudal filaments subequal in length, with many elongate, apically rounded, stout setae at articulations (Figure 59F).

**Figure 60:**
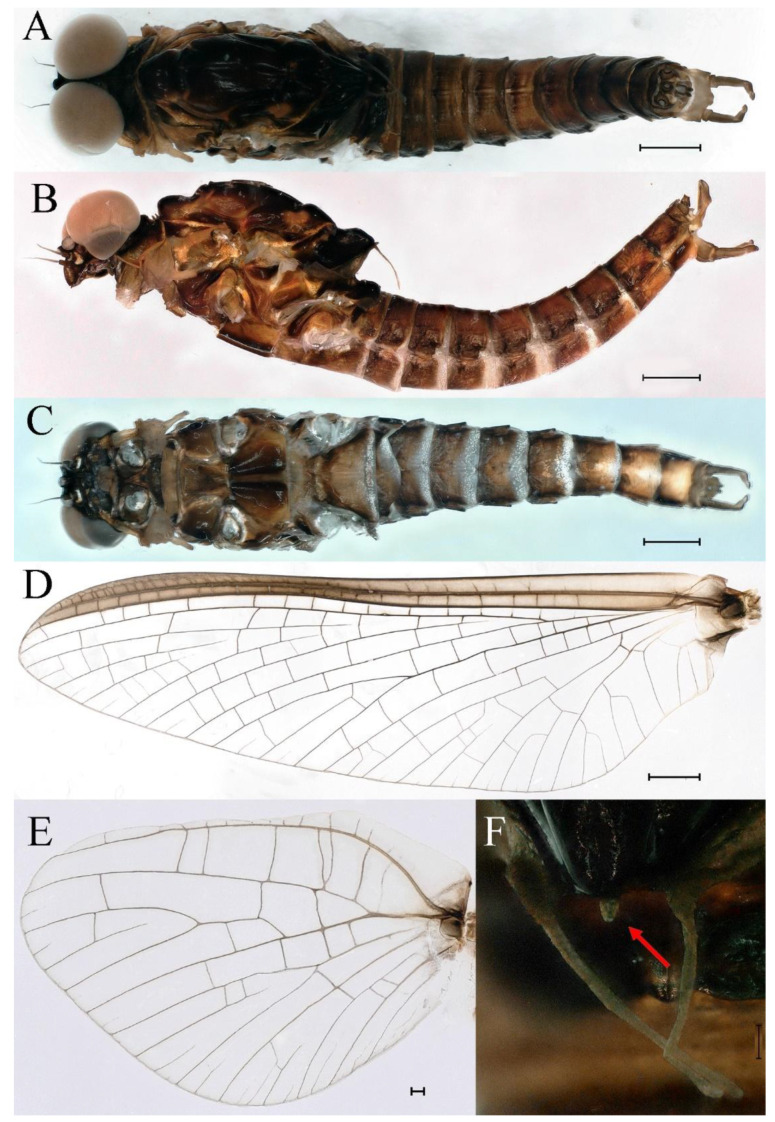
Male imago of *Cincticostella yushui* **sp. nov.**: (**A**) dorsal view of body; (**B**) lateral view of body; (**C**) ventral view of body; (**D**) forewing; (**E**) hindwing; (**F**) lateral scutellar projections, middle one indicated by red arrow. Scale bar: (**A**–**D**) = 1 mm; (**E**,**F**) = 0.1 mm.

***Male imago***. Body length 11.42–12.56 mm (excluding tails), head width 2.16–2.39 mm, forewing length 12.03–14.35 mm, hindwing length 3.59–3.60 mm, cerci length 15.00–17.64 mm, middle caudal filament 15.41–17.61 mm. Body color brown to dark brown (Figure 60A–C and Figure 68A).

**Head**. Compound eyes separated, upper portion brown and lower portion black (Figure 60B).

**Figure 61:**
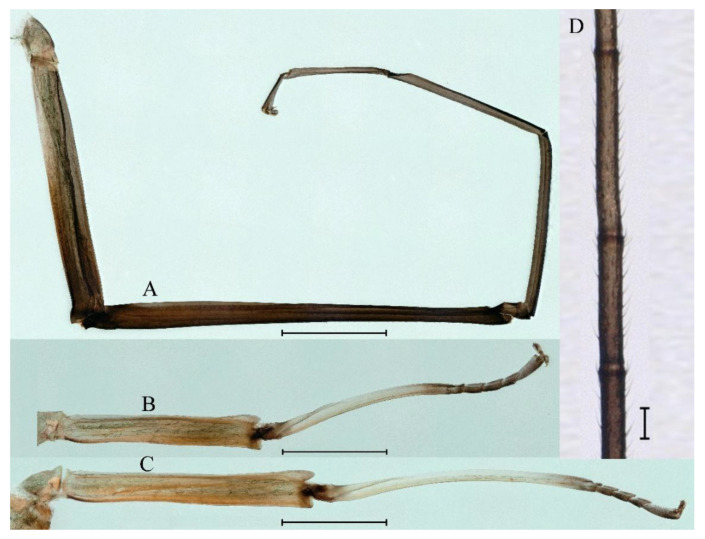
Male imago of *Cincticostella yushui* **sp. nov.**: (**A**) foreleg; (**B**) midleg; (**C**) hindleg; (**D**) caudal filament. Scale bar: (**A**–**C**) = 1 mm; (**D**) = 0.1 mm.

**Thorax**. Pronotum with expanded, posterolateral, long sac-like structure (Figure 60A). Prosternum dark brown, with slightly anteriorly converging longitudinal carinae, maximum width between carinae 3.6 times minimum width (Figure 60C). Basisternum of mesosternum dark brown, with parallel furcasternum (Figure 60C). Mesonotum with three projections on posterior margin, middle projection short (Figure 60F, indicated by red arrow). Forewings hyaline (Figure 60D), but C and Sc regions semihyaline (brown); longitudinal veins dark brown and crossveins light brown; crossveins in stigma region between C and Sc separated into two parts by long vein; Rs leaves MA at very base, MA forked one-third of distance from base to margin; MP forked slightly more distally than fork of MA + Rs; CuP recurved. Hindwing totally hyaline (Figure 60E), costal projection small, rounded, located at distance about one-third from base to apex; MP forked between forks of R1+MA and MA. Forelegs brown to dark brown (Figure 61A), mid- and hindlegs brown (Figure 61B,C). Femur:tibia:tarsus of foreleg = 1.0:1.6:2.3, tarsal segments from basal to apical = 1.0:6.8:6.6:4.1:1.5; femur:tibia:tarsus of midleg = 1.9:1.9:1.0, tarsal segments from basal to apical = 1.0:1.5:1.4:1.2:2.1; femur:tibia:tarsus of hindleg = 2.4:2.7:1.0, tarsal segments from basal to apical = 1.0:1.4:1.5:1.3:2.5. Claws of all legs similar, one blunt and one hooked.

**Abdomen.** Terga II–VI each with pale median line on dorsal surface (Figure 60A). Terga I–VII each with pale stripe on posterior margin (Figure 60A–C). All terga without posterolateral projections, except tergum VIII (Figure 60C). Posterior parts of terga V–VII with pale spots. Caudal filaments dark brown, covered with spines (Figure 61D).

**Figure 62:**
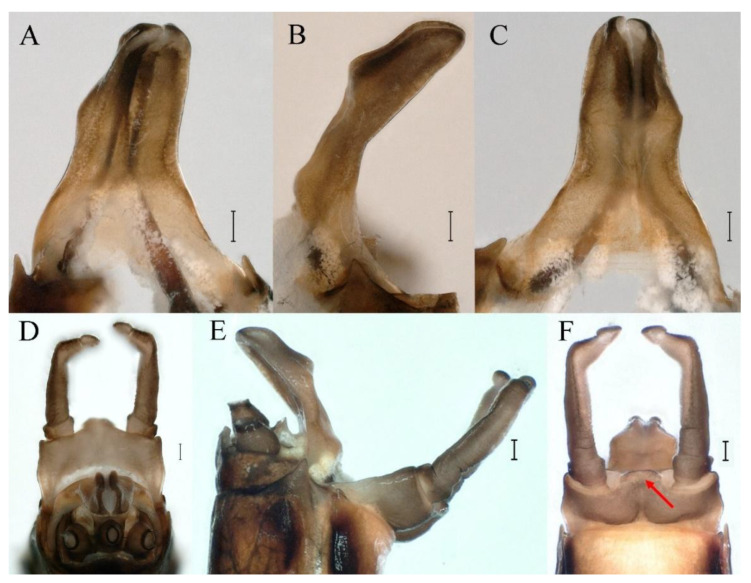
Male imago of *Cincticostella yushui* **sp. nov.**: (**A**) penes (dorsal view); (**B**) penes (lateral view); (**C**) penes (ventral view); (**D**) genitalia (dorsal view); (**E**) genitalia (lateral view); (**F**) genitalia (ventral view), median convex lobe indicated by red arrow. Scale bar: 0.1 mm.

**Genitalia.** Styliger plate with median convex lobe, the median lobe between forceps bases overall ellipsoid in appearance (Figure 62F, indicated by red arrow). Second segment of forceps slightly constricted at point in about apical fifth; segment 3 nearly globular (Figure 62A–C). Penis lobes compact (Figure 62D–F), not expanded, rounded at apex and with middle slightly swollen; penis lobes not divided, with linear groove on apical half in dorsal view (Figure 62A,D), with two longitudinal bumps on apical half and one slight basal bump in ventral view (Figure 62A,E). The two longitudinal bumps separated with large distance at apex, as shown in Figure 62C.

**Figure 63:**
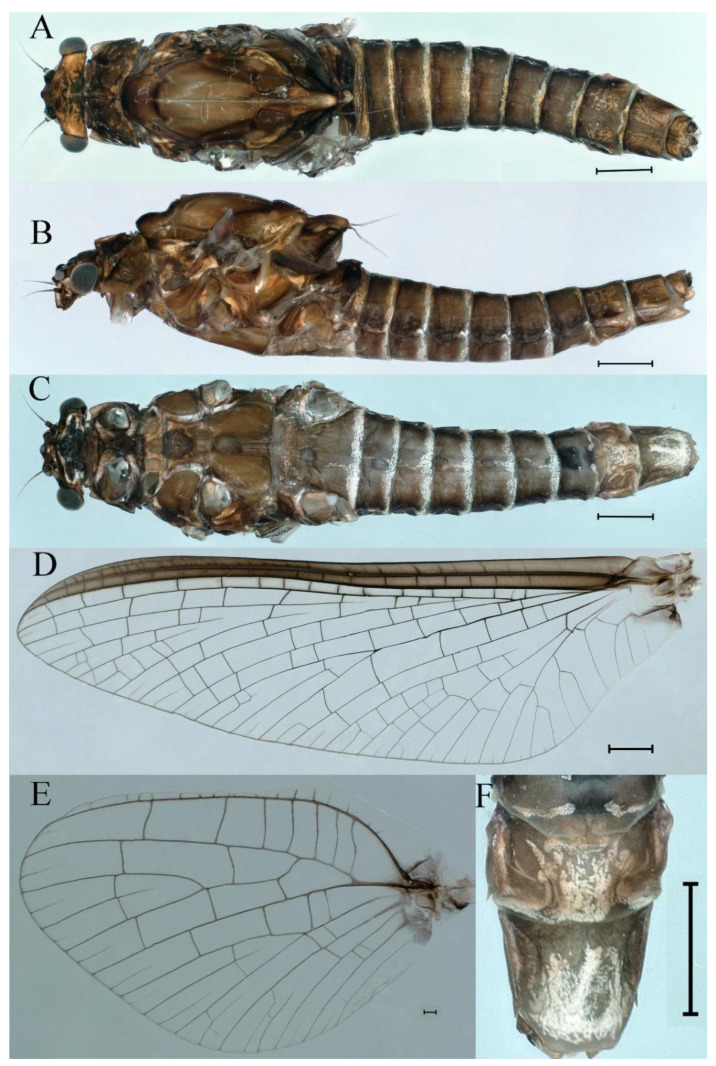
Female imago of *Cincticostella yushui* **sp. nov.**: (**A**) dorsal view; (**B**) lateral view; (**C**) ventral view; (**D**) forewing; (**E**) hindwing; (**F**) posterior part of abdomen (ventral view). Scale bar: (**A**–**D**), (**F**) = 1 mm; (**E**) = 0.1 mm.

**Figure 64:**
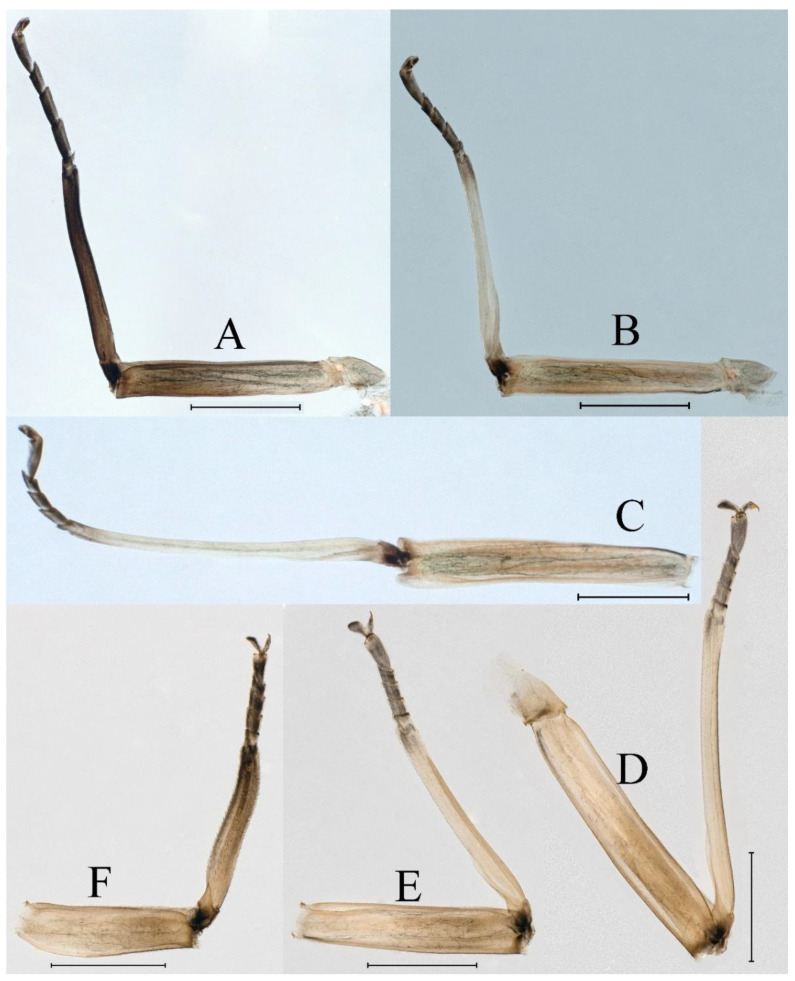
Female imago and subimago of *Cincticostella yushui* **sp. nov.**: (**A**) foreleg of imago; (**B**) midleg of imago, (**C**) hindleg of imago; (**D**) hindleg of subimago; (**E**) midleg of subimago; (**F**) foreleg of subimago. Scale bar: 1 mm.

***Female imago*** (in ethanol). Color pattern similar to male (Figure 63A–C and Figure 68B); body length 11.63–12.42 mm (excluding tails), head width 1.90–2.15 mm, cerci length 14.80–14.46 mm, middle caudal filament 15.38–15.47 mm, forewing length 13.32–15.38 mm (Figure 63D), hindwing length 3.54–3.80 mm (Figure 63E). Lengths of femur (Figure 64A):tibia:tarsus of foreleg = 1.4:1.4:1.0, tarsal segments from basal to apical = 1.0:2.2:2.2:1.4:2.3; femur:tibia:tarsus of midleg (Figure 64B) = 2.1:2.1:1.0, tarsal segments from basal to apical = 1.1: 1.2: 1.3: 1.0: 2.4; femur:tibia:tarsus of hindleg (Figure 64C) = 2.5:2.9:1.0, tarsal segments from basal to apical = 1.0:1.3:1.5:1.1:2.6. Posterior margin of subgenital plate produced to one-fifth length of sternum VIII. Posterior margin of subanal plate straight (Figure 63F). Color pattern of caudal filaments similar to male.

**Figure 65:**
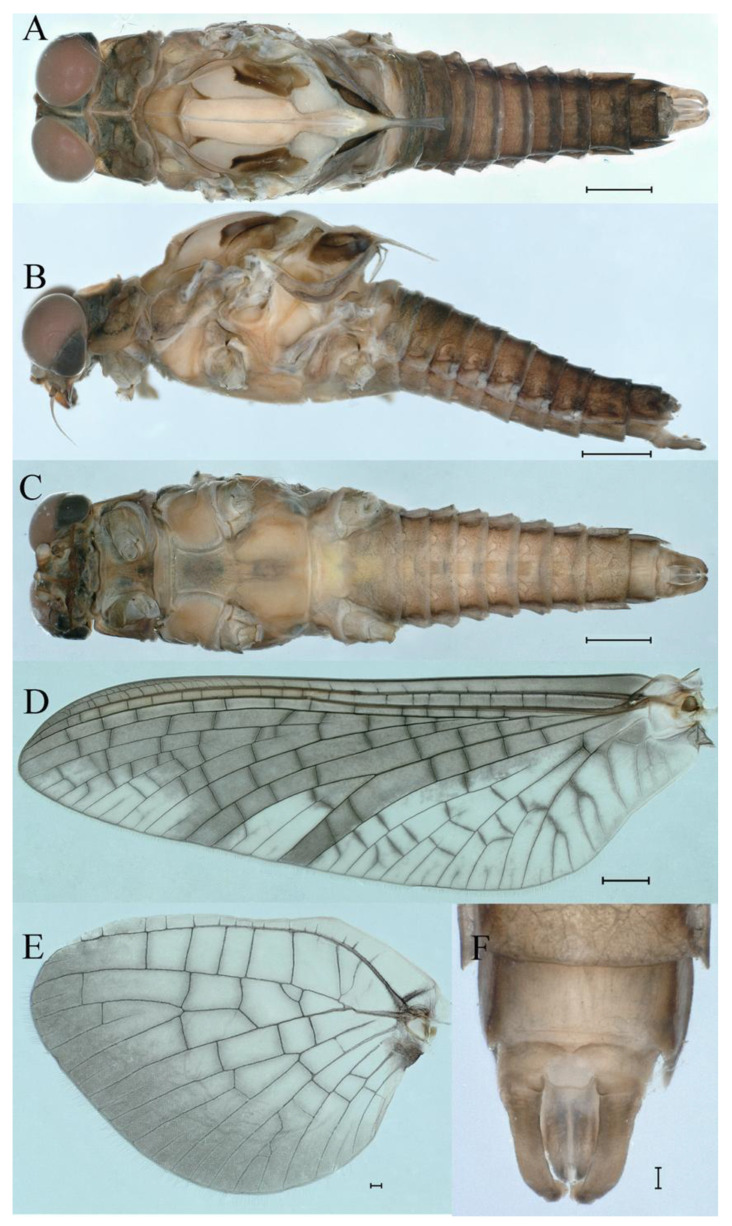
Male subimago of *Cincticostella yushui* **sp. nov.**: (**A**) dorsal view; (**B**) lateral view; (**C**) ventral view; (**D**) forewing; (**E**) hindwing; (**F**) ventral view of genitalia. Scale bar: (**A**–**D**) = 1 mm; (**E**,**F**) = 0.1 mm.

**Figure 66:**
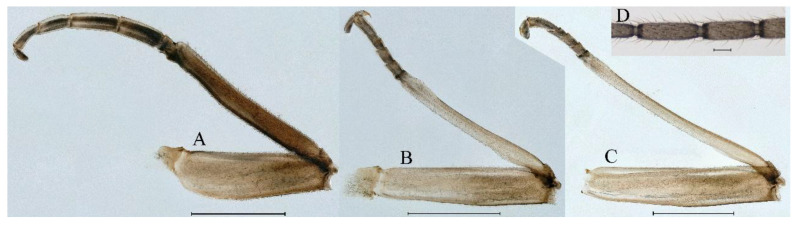
Male subimago of *Cincticostella yushui* **sp. nov.**: (**A**) foreleg; (**B**) midleg; (**C**) hindleg; (**D**). Scale bar: (**A**–**C**) = 1 mm; (**D**) = 0.1 mm.

***Male subimago*.** Body color brown to black (Figure 65A–C and Figure 68C). Mesonotum with three projections on posterior margin, middle projection longest (Figure 65A,B). Body length 8.70–11.52 mm (excluding tails), head width 2.12–2.33 mm, cerci length 9.42–9.54 mm, middle caudal filament 9.24–9.48 mm, forewing length 11.60–14.54 mm, hindwing length 3.44–3.74 mm. Forewing taupe to black brown with crossveins infuscated (Figure 65D and Figure 68C), hindwing taupe to black brown without crossvein infuscation (Figure 65E and Figure 68C). Penes as in Figure 65F. Margins of femur, tibia, and tarsus of foreleg, midleg, and hindleg densely covered with spines (Figure 66A–C). Length of femur:tibia:tarsus of foreleg = 1.0:1.1:1.0, tarsal segments from basal to apical = 1.0:2.3:2.3:1.7:1.4; femur:tibia:tarsus of midleg = 2.2:2.1:1.0, tarsal segments from basal to apical = 1.0:1.2:1.1:1.1:2.2; femur:tibia:tarsus of hindleg = 2.4:2.6:1.0, tarsal segments from basal to apical = 1.5:1.3:1.4:1.0:3.2. Caudal filaments relatively densely covered with spines (Figure 66D).

**Figure 67:**
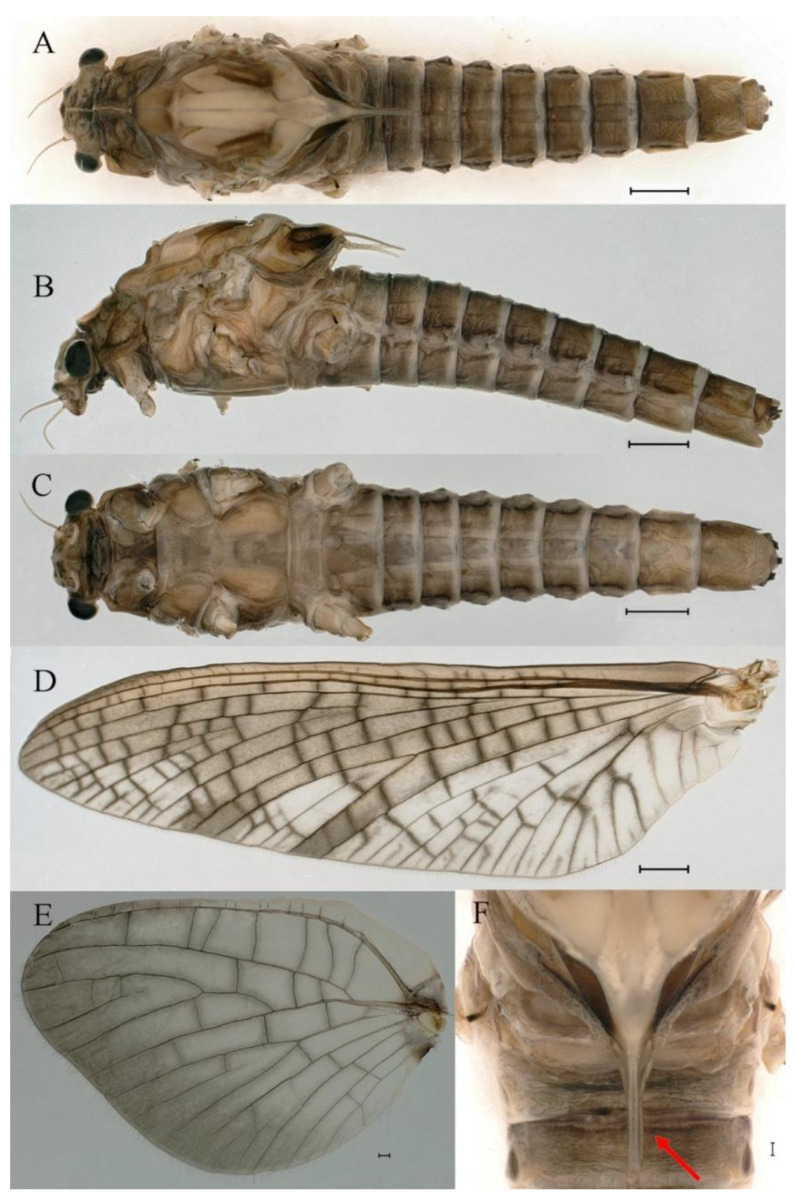
Female subimago of *Cincticostella yushui* **sp. nov.**: (**A**) dorsal view; (**B**) lateral view; (**C**) ventral view; (**D**) forewing; (**E**) hindwing; (**F**) lateral scutellar projections, middle one indicated by red arrow. Scale bar: (**A**–**D**) = 1 mm; (**E**,**F**) = 0.1 mm.

**Figure 68:**
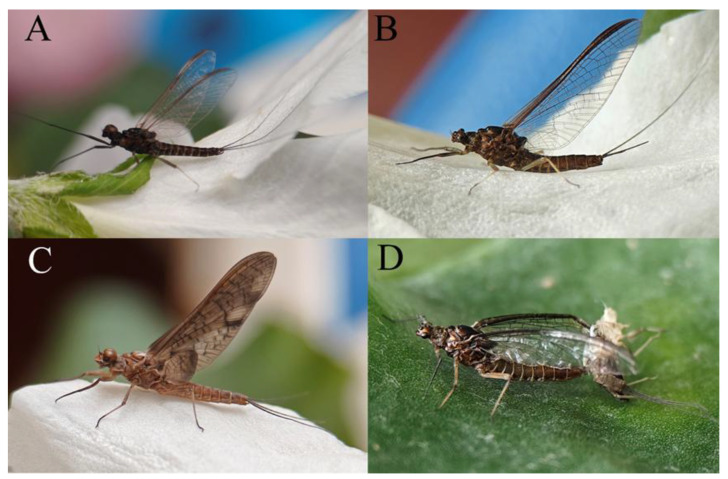
Winged stages of *Cincticostella yushui* **sp. nov.** (living): (**A**) male imago; (**B**) female imago; (**C**) male subimago; (**D**) female with subimago exuviae.

***Female subimago***. Similar to male except for usual sexual differences (Figure 67A–F and Figure 68D). Head width 2.04–2.15 mm, body length 11.35–12.61 mm (excluding tails), forewing length 13.88–15.05 mm, hindwing length 3.43–4.19 mm, cerci length 7.90–8.85 mm, middle caudal filament 7.80–9.16 mm. Length of femur:tibia:tarsus of foreleg (Figure 64F) = 1.9:1.9:1.0, tarsal segments from basal to apical = 1.0:1.4:1.1:1.0:1.7; femur: tibia: tarsus of midleg (Figure 64E) = 2.0:2.0:1.0, tarsal segments from basal to apical = 1.0:1.3:1.2:1.0:1.9; femur:tibia:tarsus of hindleg (Figure 64D) = 2.5:2.9:1.0, tarsal segments from basal to apical = 1.1:1.1:1.1:1.0:2.3.

**Figure 69:**
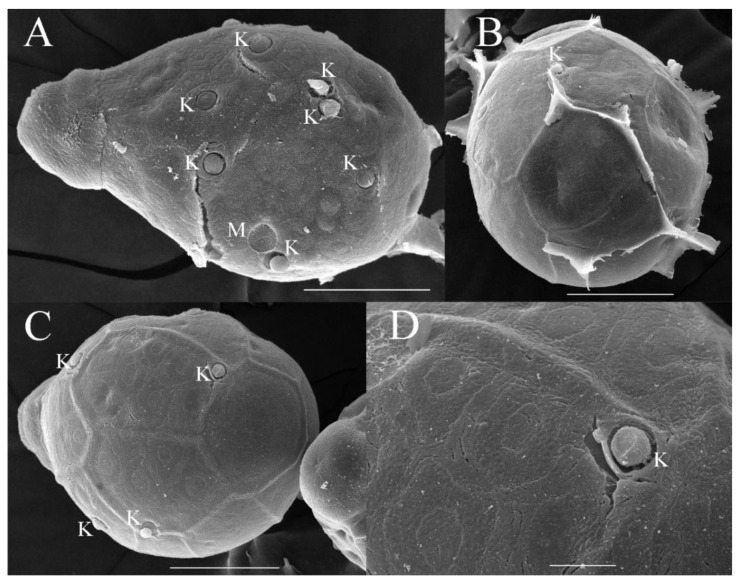
Egg of *Cincticostella yushui* **sp. nov.**: (**A**) lateral view with micropyle (M) and knob of attachment structure (K); (**B**) bottom view; (**C**) polar cap and bottom view; (**D**) attachment structure (K) enlarged. Scale bar: (**A**–**C**) = 0.05 mm; (**D**) = 0.01 mm.

***Eggs*.** dissected from female imago. Length 162–178 μm, width 100–113 μm. Ovoid with polar cap composed of dense filaments, each filament with intumescent terminal (Figure 69A). Chorion with irregular polygonal mesh, with big, stout strands (Figure 69A–C); mesh with one tubercle medially; micropyles distributed near equator; chorion with knobs of attachment structure except in subpolar areas (Figure 69A–C).

**Remarks.** The species *C. elongatula*, *C. funki*, *C. nigra*, *C. shinichii*, *C. xiazhi* **sp. nov.**, and *C. yushui* **sp. nov.** all have similar characteristics of the abdomen, with posteromedian projections of only terga V–IX being relatively well-developed; on other terga, these structures are otherwise small or absent [3,9]. We note that *C. xiazhi* **sp. nov.** and *C. yushui* **sp. nov.** have unique characters of the leg, with the tarsal claws of *C. xiazhi* **sp. nov.** having three denticles, *C. yushui* **sp. nov.** with four, *C. shinichii* with one, *C. funki* with two, and *C. elongatula* and *C. nigra* with more than five.

**Distribution**. Yunnan, China.

### 3.5. Remnant Mouthparts of Winged Stages

**Figure 70:**
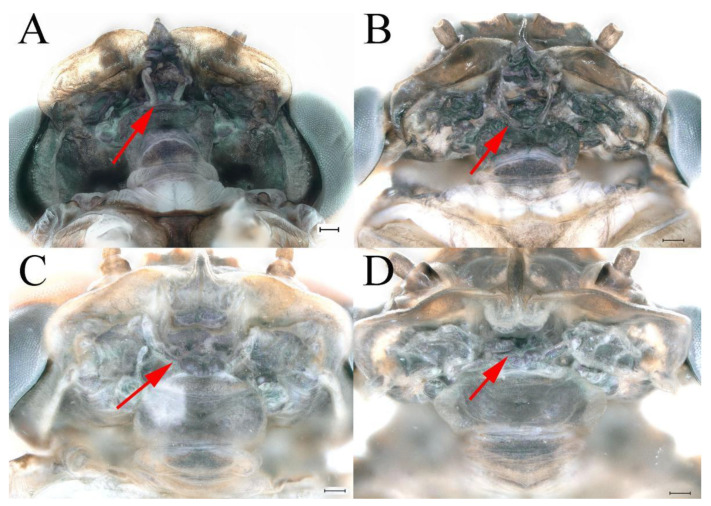
Persistent mouthparts of *Cincticostella wangi*, labium indicated by arrow: (**A**) male imago; (**B**) female imago; (**C**) male subimago; (**D**) female subimago. Scale bars: 0.1 mm.

**Figure 71:**
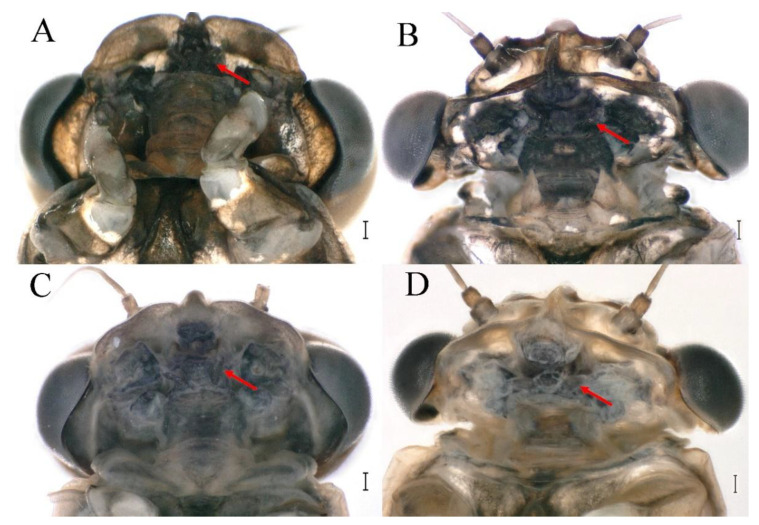
Persistent mouthparts of *Cincticostella funki*, labium indicated by arrow: (**A**) male imago; (**B**) female imago; (**C**) male subimago; (**D**) female subimago. Scale bars: 0.1 mm.

**Figure 72:**
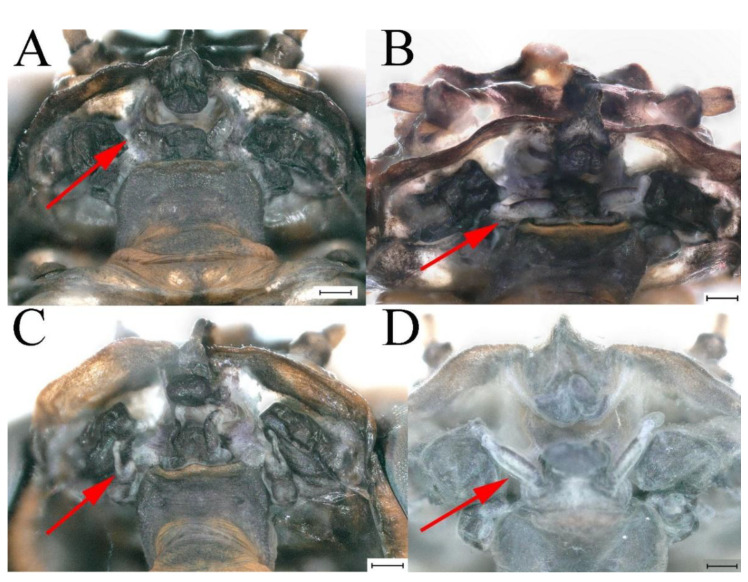
Persistent mouthparts of *Cincticostella xiazhi* **sp. nov.**, labium indicated by arrow: (**A**) male imago; (**B**) female imago; (**C**) male subimago; (**D**) female subimago. Scale bars: 0.1 mm.

**Figure 73:**
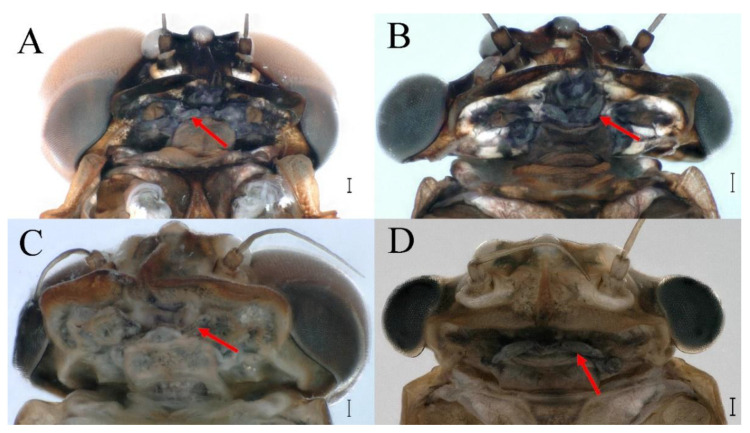
Persistent mouthparts of *Cincticostella yushui* **sp. nov.**, labium indicated by arrow: (**A**) male imago; (**B**) female imago; (**C**) male subimago; (**D**) female subimago. Scale bars: 0.1 mm.

The four species *C. wangi*, *C. funki*, *C. xiazhi* **sp. nov.** and *C. yushui* **sp. nov.** have vestigial mouthparts in the winged stages; in ventral view of the head, the labium is clearly visible (Figure 70A–D, Figure 71A–D, Figure 72A–D and Figure 73A–D indicated by red arrow).

### 3.6. Results of DNA Analysis

**Figure 74:**
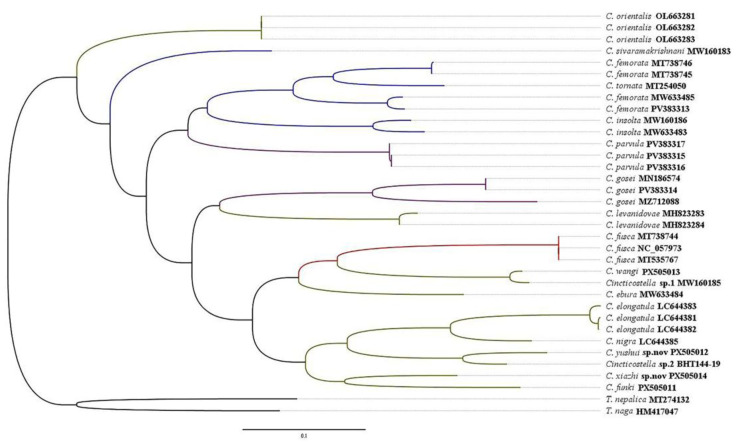
Our COI phylogenetic tree of some *Cincticostella* species. GenBank accession numbers after the species name. The color bars indicate the species complex of the genus *Cincticostella*: red = *C. jianchuan* complex, green = *C. nigra* complex, blue = *C. insolta* complex, and purple = *C. gosei* complex. *Torleya naga* and *T. nepalica* are outgroups. The scale bar indicates 10% sequence divergence.

Our preliminary phylogenetic tree reconstruction, based on COI data and using maximum likelihood method, recovered *Cincticostella* as a monophyletic lineage with high-probability branch support (Figure 74). Our reconstruction contained seventeen species of *Cincticostella*, and the interspecific genetic distances ranged from 15–28%. *Cincticostella funki*, *C. wangi*, *C. xiazhi* **sp. nov.**, and *C. yushui* **sp. nov.** differed from other species by a range of 18–26%, 20–28%, 18–27%, and 19–27% (Table 2). The COI sequences of *Cincticostella wangi* and *C. yushui* **sp. nov.** show a high degree of consistency (Figure 74) with two species from India that were provided by Auychinda et al. [6]. The interspecific genetic distances between *C. wangi and Cincticostella* sp.1 (MW160185) and *C. yushui* **sp. nov.** and *Cincticostella* sp.2 (BHT144-19) are only 0.09 and 0.02, respectively.

### 3.7. Ecology

**Figure 75:**
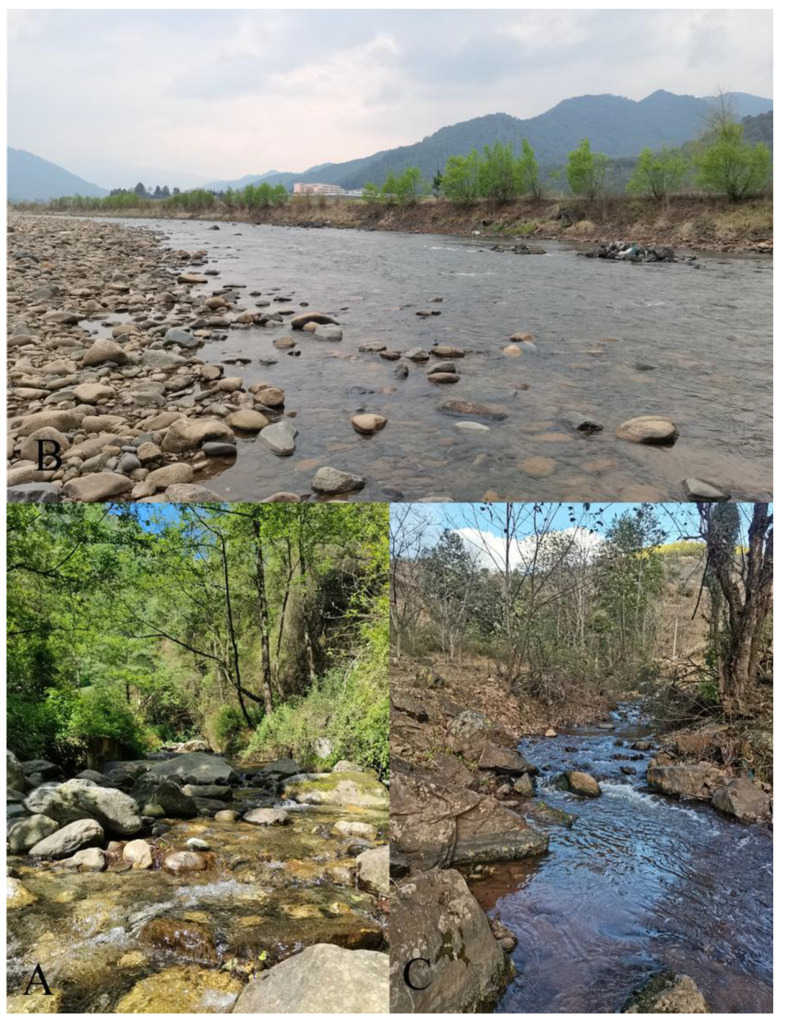
Habitats of Collection localities: (**A**) Qingbi Stream in Cangshan; (**B**) Longjiang River; (**C**) a Shunbi River feeder stream.

The nymphs of *C. wangi, C xiazhi* **sp. nov.**, and *C. yushui* **sp. nov.** prefer stream run and riffle habitats containing stones and litter (Figure 75A,C), but *C. funki* is adapted to run and riffle habitats without litter (Figure 75B). Sampling sites of *C. wangi* were located at medium to high altitudes (from 2200–3300 m a.s.l.); *C xiazhi* **sp. nov.** and *C. yushui* **sp. nov.** were located at medium altitudes (*C xiazhi* **sp. nov.** at 2200 m a.s.l. and *C. yushui* **sp. nov.** at 2284 m a.s.l.); and *C. funki* were located at low to medium altitudes (from 1172–1853 m a.s.l.). The streams and associated riparian zones of all sampling sites passed through relatively natural habitats or through traditional agricultural or village settings (Figure 75A–C). Thus, the presence of these species may indicate a healthy ecosystem.

In the laboratory, the mature nymphs of *C. wangi* and *C. xiazhi* **sp. nov.** quickly completed the molting process on the water surface from 6–8 a.m. The subimago stage of *C. wangi* persisted until the next day around noon, and *C. xiazhi* **sp. nov.** persisted until the next afternoon. In contrast, the mature nymphs of *C. funki* and *C. yushui* **sp. nov.** quickly completed the molting process on the water surface from 8–10 p.m., but the subimago stages remarkably persisted until noon, four days later.

## 4. Discussion

### 4.1. Plasticity and Stability of Morphological Characteristics in Cincticostella

After examining series of nymphal specimens of *C. funki, C wangi*, *C. xiazhi* **sp. nov.**, and *C. yushui* **sp. nov.**, we found generally that inconsistency in the number of denticles on claws of all legs is mainly caused by damage rather than genetics.

For some widely distributed species, some characteristics may exhibit plasticity, such as the total number of posteromedian projections on abdominal terga of nymph and the time of adult emergence. The least developed projections appear to be the most variable, with the location and quantity of relatively well-developed projections seeming to be relatively stable. *Cincticostella wangi* serves as a typical example.

### 4.2. Key to Final Nymphal Instars of the Cincticostella nigra Complex

Below, we present a key to final nymphal instars of all species in the *C. nigra* complex, in some cases based on relevant literature [2,3,4,14,15].

#### Key to Final Nymphal Instars of *C. nigra* Complex

1. Abdominal terga without paired posteromedian projections (e.g., Xie et al.: figure 14A,B) [15]………………………...…………………....……………....…......*Cincticostella szechuanensis*
1′. Abdominal terga with paired posteromedian projections…………………...…...............2
2. Each posteromedian projection relatively weakly developed (e.g., Figure 5A)…....….....3
2′. At least some abdominal terga with relatively well-developed posteromedian projections (e.g., Figure 22A).…………………………….…………….…………......…......................5
3. Labrum without obvious anteromedian emargination (e.g., Kang and Yang: figure 4B) [14], tarsal claw with two denticles (e.g., Kang and Yang: figure 4K) [14]..................................
..............................................…………………………….……........................*Cincticostella colossa*
3′. Labrum with deep anteromedian emargination (e.g., Figure 2B), tarsal claw with one denticle (e.g., Figure 4G–I)………………...........…………...……….….....................................4
4. Anterolateral projections of mesothorax poorly developed, projections on terga VI–VIII strongest (e.g., Martynov et al.: figures 5B,C and 8A–D) [3]................*Cincticostella corpulenta*
4′. Anterolateral projections of mesothorax well-developed, projections on terga V–VII strongest (e.g., Figure 3A and Figure 5A)…...….......………….......………...…....*Cincticostella wangi*
5. Anteriormost relatively well-developed posteromedian projections occur on tergum IV (e.g., Auychinda et al.: 1b) [4]…........…………............…..............................*Cincticostella ebura*
5′. Anteriormost relatively well-developed posteromedian projections occur on terga posterior to tergum IV (e.g., Figure 58A) …........…….....……............…..........................................6
6. Anteriormost relatively well-developed posteromedian projections on tergum VI; eastern Palearctic…........…………...........……….....................…...................*Cincticostella orientalis*
6′. Anteriormost relatively well-developed posteromedian projections on tergum V; Palearctic and Indomalayan….........…………............………...........….............…............................7
7. Posteromedian projections on terga V–VIII relatively well-developed, small on tergum IX; eastern Palearctic…........…………......................…..............…......*Cincticostella levanidovae*
7′. Posteromedian projections on terga V–IX relatively well-developed (e.g., Figure 22A,B); eastern Palearctic and Indomalayan.................................................….......................................8
8. Tergum X with pair of posteromedian projections (e.g., Martynov et al.: figure 4A,C) [3]; Indomalayan…........................................................................….................*Cincticostella changfai*
8′. Posterior margin of tergum X without paired projections (e.g., Figure 22A); Palearctic and Indomalayan............................................................................................…...........................9
9. Tarsal claw with one denticle (e.g., Martynov et al.: figure 18H) [3], labrum with anteromedian emargination relatively deep and wide (e.g., Martynov et al.: figure 17C) [3]; Indomalayan....….........................……….........................…..........................*Cincticostella shinichii*
9′. Tarsal claw with more than one denticle, labrumanteromedian emargination shallow and narrow (e.g., Figure 55C); eastern Palearctic and Indomalayan.….....…..............…....10
10. Tarsal claw with more than four denticles (e.g., Allen: figure 6) [2]; eastern Palearctic....….....…...........................….......................….........................………......…..........................11
10′. Tarsal claw with two to four denticles; Indomalayan....…...................................…......12
11. Dorsal surface of femora with short, dense clavate setae (e.g., Ishiwata: figure 50A) [9]…......…......…......…..........….......…………....................….................*Cincticostella elongatula*
11′. Dorsal surface of femora smooth, without short clavate setae (e.g., Allen: figure 10; Ishiwata: figure 52A) [2,9]...................................…..........….....................*Cincticostella nigra*
12. Tarsal claw with four denticles (e.g., Figure 57J–L); body yellow brown without a dorsal, median pale line....….....….....….....….…......….....…...............*Cincticostella yushui* **sp. nov.**
12′. Tarsal claw with two or three denticles; body black with a dorsal, median pale line....….....….....….....….................….....…........…………...........…….........….........................13
13. Tarsal claw with two denticles (e.g., Figure 21G–H); dorsal surface of middle and hind femora covered with numerous short, stout setae (Figure 21J)...…............*Cincticostella funki*
13′. Tarsal claw with three denticles (e.g., Figure 38D–F); dorsal surface of middle and hind femora, smooth, with only a few short, stout setae (Figure 38A–C,H).........…..........................…...................….....…........……….......*Cincticostella xiazhi* **sp. nov.**

### 4.3. Species Complexes and Distinguishing Nymphal Characters of Cincticostella

Our discussion and evaluation of species complexes follow the work of Martynov et al. [3], Auychinda et al. [4], Sun et al. [5], and the research findings of this paper. Sun et al. [5] proposed a *C. jianchun* complex separated from the *C. nigra* complex. Auychinda et al. [4], in 2025, found that their species, *C. parvula*, belongs to the *C. gosei* complex but had a maxillary palp, which changed the unique taxonomic characteristics (maxillary palp absent) of the *C. gosei* complex. The latest systematic structure of *Cincticostella* is shown in Table 3, and the distinguishing nymphal characters of *Cincticostella* species complexes are shown in Table 4. At present, the genus *Cincticostella* contains at least 27 species (one being a *nomen dubium*). Based on our evaluation, *C. xiazhi* **sp. nov.** and *C. yushui* **sp. nov.** belong to the *C. nigra* complex.

### 4.4. Distinguishing Winged Stages of Cincticostella

Our detailed research allows us to make some generalizations about the genus *Cincticostella*, based especially on the descriptions of the winged stages of *C. funki, C wangi*, *C. xiazhi* **sp. nov.**, and *C. yushui* **sp. nov.**, along with our color images. 

We found that forewings of subimagoes belonging to the *C. nigra* complex have consistent color characteristics among our specimens of different species from China. However, *C. nigra* complex species from Japan had a variety of color characteristics [9]. Pertaining to the Japanese species, the forewings of *C. nigra* and *C. orientalis* subimagoes are black, and *C. elongatula* and *C. levanidovae* are brown, with crossveins infuscated [9]. Moreover, we found that all of our Chinese *C. nigra* complex specimens consistently have the mesonotum of both the subimago and imago with three projections on the posterior margin, while *C. levanidovae* and *C. orientalis* from Japan have only the middle projection in the subimago stages (Ishiwata: figures 22 and 24 [9]) and no such projections in the imago stages (Ishiwata: figures 18 and 20 [9]). Maxillary palpi of *C. levanidovae* and *C. orientalis* are not more than the length of the galea-lacina (Ishiwata: figures 40 and 42 [9]), in contrast to other *C. nigra* complex species. The characteristics of the mesonota and maxillary palpi of *C. levanidovae* and *C. orientalis* are more typical of the *C. insolta* complex and the *C. gosei* complex. Also, our preliminary phylogenetic tree of COI showed that *C. levanidovae* and *C. orientalis* were more closely related to species of the *C. insolta* complex and *C. gosei* complex [4,6]. Therefore, the placement of *C. levanidovae* and *C. orientalis* into any species complex needs further evaluation. Furthermore, *C. szechuanensis*, with its unique nymphal abdominal characteristics, should be studied further to evaluate its taxonomic affinities.

Wing coloration may prove to be important to understanding the systematics of the genus *Cincticostella*. Forewings of subimagoes have different color characteristics among the *C*. *insolta* complex (Zheng and Zhou: figure 4B) [8], *C. nigra* complex (Figure 12D, Figure 14D, Figure 16C,D, Figure 28D, Figure 32D, Figure 33C,D, Figure 47D, Figure 49D, Figure 51C,D, Figure 65D, Figure 67D, and Figure 68C), and *C. jianchuan* complex (Sun et al.: figures 13A, 16A, and 17C,D [5]). The *C. gosei* complex (Zhang et al.: figure 3C,D) [12] is the same as the *C. insolta* complex in this regard. Hindwing rear portions that are nearly white are only found in the *C. jianchuan* complex (Sun et al.: figures 13B, 16B, and 17C,D) [5], except for *C. funki* (Figure 28E, Figure 32E and Figure 33C,D) in the *C. nigra* complex. The areas between C, Sc, and R1 of imagoes in the *C. insolta* complex (Zheng and Zhou: figure 4A) [8] and *C. gosei* complex (Zhang et al.: figure 3A,B) [12] and part of the *C. nigra* complex (Figure 7D, Figure 10D, Figure 25D, Figure 30D, Figure 41D and Figure 45A) are semihyaline, while this area may have colored cells in the *C. jianchuan* complex (Zhang et al.: figures 4A,B and 5A [13]; Sun et al.: figures 7E, 10D, and 17A,B [5]) and part of the *C. nigra* complex (Figure 60D and Figure 63D). The *C. jianchuan* complex male imagoes have the apical sclerite (Zhang et al.: figure 5C,E [12]; Sun et al.: figure 9C,F [5]) and one basal obvious bump (Zhang et al.: figure 5E [12]; Sun et al.: figure 9B,E [5]) on the ventral face of the penes, features also found in *C. yushui* **sp. nov.** (Figure 62C).

### 4.5. Research Prospects for Cincticostella

The interspecific genetic distances between *C. wangi* and *Cincticostella* sp.1 (MW160185) and *C. yushui* **sp. nov.** and *Cincticostella* sp.2 (BHT144-19) are very small. However, due to the lack of knowledge of their morphology and ecology, we refrain from formally assigning them a species identification and continue to follow the terminology of Auychinda et al. [6]. But we consider it highly likely that *Cincticostella* sp.1 and *Cinticostella* sp.2 are referable to *C. wangi* and *C. yushui* **sp. nov.**, respectively. As in this example, a preliminary phylogenetic tree based only on the COI gene is useful for forming species identification and delimitation hypotheses, but as a general rule, it is insufficient for evaluating species group and other classifications of the genus *Cincticostella* [4,6]. Thus, the groupings indicated in our preliminary phylogenetic tree based on COI alone (Figure 74) should not be taken to suggest systematic changes to *Cincticostella*. DNA barcoding cannot always distinguish very closely related species and may oversplit or undersplit species [30,31,32]. More comprehensive analysis using a variety of nuclear and other mitochondrial DNA sequences, possibly including complete mitochondrial genomes, remains necessary for the formation of robust phylogenetic hypotheses about *Cincticostella* and related genera. Once these additional data and a slightly wider scope of taxon sampling are available, a combined evidence approach should be feasible for proposing any revisions to classification. In the meantime, the fundamental research of species-level diversity in the genus should continue, especially in light of the discoveries detailed in this study.

The penes of *C. funki, C wangi*, *C. xiazhi* **sp. nov.**, and *C. yushui* **sp. nov.** have significant differences, such as the median convex lobes of the styliger plate (Figure 9C, Figure 27F, Figure 43C and Figure 62D,F) and the penis lobes themselves (Figure 9D–F, Figure 27A–C, Figure 43D–F and Figure 62A–C), suggesting considerable previously overlooked diversity in this complex. Therefore, we predict the discovery of several new or historically misunderstood species, especially in northwest, southern, and central China, as well as Vietnam and the India–Pakistan border regions. The improved knowledge brought about by the work detailed in this paper, and our yet unpublished work, allowed us to recognize some potential past errors in classification and taxonomy. Poorly known species*,* such as *Ephemerella svenhedini* Ulmer, 1936 in Gansu, China [33] and *Drunella soldani* Allen, 1986 in Vietnam [34], may eventually prove to be species of *Cincticostella*, based in part on their male genitalia and nymphal mouthparts, respectively. Jacobus and McCafferty [27] considered *E. svenhedini* to be a junior synonym of *D. submontana* (Brodsky, 1930) [35], and *D. soldani* was moved to the monospecific genus *Adoranexa* Jacobus & McCafferty, 2008 [1]. Moreover, *Ephemerella swatensis* (Ali, 1971) from Pakistan was considered to be a junior synonym of *C. levanidovae* by Jacobus and McCafferty (2003) [36]. On the one hand, *E. swatensis* (Ali: figures 17–23) [37] appears to have mesonotal anterolateral projections and forewing pads that do not match the emerging morphological characterization of *Cincticostella.* On the other hand, the legs of *E. swantensis* appear somewhat similar to the characterization of the *C. jianchuan* complex. Unfortunately, we have seen no bona fide specimens of *E. swatensis* and are basing our evaluation entirely on the published illustrations [37], which may be questionable.

As detailed in the introduction, more than half of *Cincticostella* species have been described since the genus-level classification was formally revised [1]. Moreover, considerable advances are being made in our knowledge of the winged stages and the acquisition of DNA barcodes, as demonstrated in this work. Thus, adjustment to some terminology used to describe various groups of *Cincticostella* species (e.g., species “group” rather than species complex) soon will be appropriate and also necessary to maintain consistent communication about diverse Ephemeroptera taxa. For example, species may first be considered part of species complexes; species complexes then are part of species groups; and species groups are the level below subgenus or genus [38]. We prefer to institute any such changes after additional clarifying work is published, hopefully in the near future.

## Figures and Tables

**Table 1:** Collection details of the sequenced specimens.

Species	Code	Collection Locality	Collector	Date	GenBank Accession Number
*Cincticostella funki*	CfLC01	Tengchong City	Yan-Chang Zi	4 February 2025	PX505011
*Cincticostella xiazhi* **sp. nov.**	CxQB01	Dali City	Xian-Fu Li	21 June 2024	PX505014
*Cincticostella wangi*	CwQB01	Dali City	Xian-Fu Li	12 May 2025	PX505013
*Cincticostella yushui* **sp. nov.**	CySB01	Yunlong County	Xian-Fu Li	22 February 2025	PX505012

**Table 2:** Pairwise genetic distances (COI) between species of *Cincticostella* using the Kimura 2-parameter.

Species	1	2	3	4	5	6	7	8	9	10	11	12	13	14	15
	K2P Genetic Distances														
1. *C. ebura*															
2. *C. elongatula*	0.25														
3. *C. femorata*	0.25	0.23													
4. *C. funki*	0.22	0.25	0.26												
5*. C. fusca*	0.22	0.24	0.25	0.25											
6. *C. gosei*	0.25	0.28	0.25	0.26	0.27										
7. *C. insolta*	0.23	0.25	0.23	0.24	0.25	0.25									
8. *C. levanidovae*	0.25	0.26	0.24	0.25	0.25	0.27	0.27								
9. *C. nigra*	0.24	0.15	0.25	0.22	0.24	0.26	0.27	0.23							
10. *C. orientalis*	0.28	0.26	0.25	0.25	0.24	0.27	0.25	0.24	0.26						
11. *C. parvula*	0.28	0.23	0.23	0.25	0.23	0.25	0.24	0.26	0.25	0.24					
12. *C. sivaramakrishnani*	0.23	0.26	0.22	0.25	0.25	0.25	0.23	0.24	0.27	0.21	0.23				
13. *C. tornata*	0.25	0.28	0.15	0.25	0.24	0.24	0.22	0.25	0.28	0.24	0.23	0.23			
14. *C. wangi*	0.20	0.22	0.25	0.22	0.24	0.27	0.27	0.24	0.22	0.26	0.28	0.23	0.26		
15. *C. xiazhi* **sp. nov.**	0.18	0.22	0.25	0.18	0.22	0.27	0.25	0.25	0.21	0.26	0.24	0.25	0.26	0.23	
16*. C. yushui* **sp. nov.**	0.22	0.22	0.26	0.21	0.27	0.24	0.24	0.25	0.22	0.24	0.25	0.23	0.27	0.23	0.19

**Table 3:** Systematic structure of *Cincticostella*.

*C. insolta* Complex	*C. nigra* Complex
*C. bifurcata* Xie, Jia, Chen, Jacobus & Zhou, 2009	*C. changfai* Martynov & Palatov, 2021
*C. braaschi* Jacobus & McCafferty, 2008	*C. colossa* Kang & Yang, 1995
*C. femorata* Tshernova, 1972	*C. corpulenta* Braasch, 1981
*C. insolta* Allen, 1971	*C. elongatula* McLachlan, 1875
*C. ranga* Selvakumar & Subramanian, 2019	*C. funki* Martynov, Selvakumar, Palatov & Vasanth, 2021
*C. richardi* Martynov & Palatov, 2019	*C. ebura* Auychinda, Sartori & Boonsoong, 2022
*C. sivaramakrishnani* Martynov & Palatov, 2019	*C. levanidovae* Tshernova, 1952 *
*C. tornata* Auychinda & Gattolliat, 2020	*C. nigra* Uéno, 1928
***C. gosei* complex**	*C. orientalis* Tshernova, 1952 *
*C. gosei* Allen, 1975	*C. shinichii* Martynov & Palatov, 2021
*C. parvula* Auychinda, Buchawongpiwat, Sartori & Boonsoong, 2025	*C. szechuanensis* Xie, Jia, Chen, Jacobus & Zhou, 2009 *
***C. jianchuan* complex**	*C. wangi* Selvakumar, Martynov & Subramanian, 2021
*C. fusca* Kang & Yang, 1995	*C. xiazhi* Zi, Li & Jacobus **sp. nov.**
*C. jianchuan* Sun, Tan, Li & Jacobus, 2024	*C. yushui* Zi, Li & Jacobus **sp. nov.**
	*Cincticostella* sp. A Martynov et al. [3]

* Doubtful placement in this complex.

**Table 4:** Distinguishing larval characters of *Cincticostella* species complexes.

	Characters	*C. insolta* Complex	*C. nigra* Complex	*C. gosei* Complex	*C. jianchuan* Complex
1	Two pairs of suboccipital tubercles	present	absent	absent	absent
2	Serration of margins of middle and hind femora	present	absent	absent	absent
3	Presence of numerous large, rounded scale sockets on body surface	present	absent	present	absent
4	Rate of anterolateral emargination of labrum	shallow	from shallow to deep	moderate	absent
5	Maxillary palp	reduced, articulations of segments not distinct, especially between segments I and II	mainly well-developed, articulations of all segments distinct	absent or reduced, articulations of segments not distinct, especially between segments I and II	mainly well-developed, articulations of all segments distinct
6	Segments I and II of labial palp	wide	wide	relatively narrow, elongated	wide
7	Stout setae on outer margin of fore femur	several stout setae only	numerous stout setae	several stout setae only	numerous stout setae
8	Stout setae on dorsal surface of middle and hind femora	absent or up to several stout setae in basal area	surface with numerous stout setae	absent	surface with numerous stout setae
9	Shape of hind femur	strongly or moderately widened	moderately widened	moderately widened	moderately widened
10	Stout setae on dorsal surface of abdominal terga and paired projections	absent	present	absent	present or absent
11	Body size	moderate	moderate	small	large
12	Mesothoracic anterolateral projections	not notched	not notched	not notched	notched
13	subapical bands of transverse setae	absent	present	present	absent

## Data Availability

All data are available in the paper.
